# Repositioning Antihistamine for Cancer Therapy: Clemizole as a Template for the Design of Liver Tissue-Targeting Epigenetic-Modifying Agents

**DOI:** 10.1021/acs.jmedchem.5c02018

**Published:** 2026-01-28

**Authors:** Dipak T. Walunj, Bocheng Wu, Jeremiah O. Olugbami, Alexis Johnston, Ryan Kern, Travis J. Nelson, Benjamin H. Peer, Justin Keener, Peixian He, Nathaniel A. Hathaway, Adegboyega K. Oyelere

**Affiliations:** 1 School of Chemistry and Biochemistry, Georgia Institute of Technology, Atlanta, Georgia 30332-0400, United States; 2 The University of North Carolina Eshelman School of Pharmacy, Chapel Hill, North Carolina 27599, United States; 3 Parker H. Petit Institute for Bioengineering and Bioscience, Georgia Institute of Technology, Atlanta, Georgia 30332-0400, United States

## Abstract

Histamine receptor H_1_ (HRH1) is upregulated within the tumor microenvironment, where it supports tumorigenesis by several mechanisms. Cationic amphiphilic drugs targeting HRH1 are currently under investigation for repurposing into cancer therapy. Herein, we showed that Clemizole, a first-generation HRH1 antagonist that selectively accumulates within the liver, could be used as a template to design small-molecule epigenetic modifiers targeting histone deacetylases (HDACs) and histone lysine demethylases (KDMs). The resulting HDACi and KDMi have midnanomolar to single-digit micromolar IC_50_s and potency enhancement of 15–105 folds relative to Clemizole. Several of these compounds elicited cancer cell line-dependent cytotoxicity. Representative lead KDMi, **Cle-C6K**, and **Cle-C8K** caused transcriptome-level perturbations favoring cell cycle inhibition and apoptosis. Moreover, **Cle-C8K** is nontoxic and selectively accumulated in the liver of C57BL/6 mice. Collectively, our data reveal that Clemizole could be repositioned to design liver tissue-accumulating epigenetic-modifying small molecules as potential targeted antiliver cancer agents.

## Graphical abstract

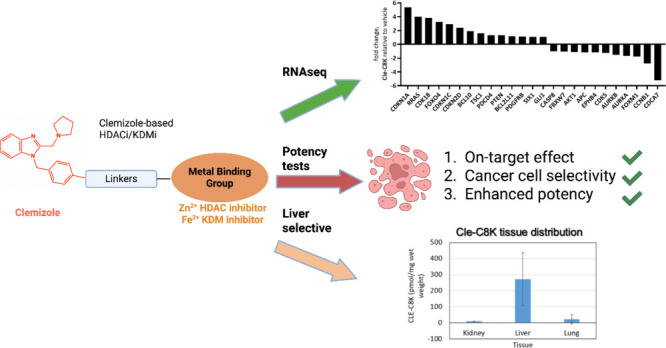

## 1 Introduction

Several of the current anticancer drugs are hampered by limitations ranging from off-target effects with the accompanying dose-limiting toxicities, to outright lack of therapeutic response. New agents with improved therapeutic indices are actively being sought in the quest for novel treatment modalities for cancers. Repurposing Food and Drug Administration (FDA)-approved drugs is a less risky, cost-effective approach to identifying novel oncology drugs with better safety profiles.^1–6^ Cationic amphiphilic drugs (CADs) targeting histamine receptor H_1_ (HRH1) are a class of benign drugs that are under investigation for repurposing into cancer therapy.

Recent observations have revealed that HRH1 and consequently histamine are upregulated within the tumor microenvironment where they promote tumorigenesis by several mechanisms including the induction of T cell dysfunction through activation of the tumor-associated macrophages toward immunosuppressive M2-like phenotype.^7^ Several CADs have demonstrated antiproliferative effects against multiple immunogenic and nonimmunogenic cancers in *in vitro* and *in vivo* settings.^8–12^ Sustained use of specific CADs such as desloratadine and loratadine have been linked with improved overall survival for several tumor types.^8,13–15^ Although there is a paucity of clinical trials evaluating the anticancer potentials of CADs,^16^ the current interest in the clinical trial of clemizole (Cle), a first generation HRH1 antagonist that selectively accumulates within the liver, for hepatic cancer therapy is a welcome development (NCT03069508).

In addition to acceptance and intellectual property barriers,^4,17^ a key challenge to direct drug repurposing for oncology applications is the low anticancer effects of the candidate drugs at the doses approved for the original indication(s).^3,4^ One strategy to overcome this challenge is to evaluate the candidate drugs as part of combination therapy regimen with other FDA-approved oncology drugs, with sensitization or improvement in treatment efficacy as a key end point.^7,18,19^ Another approach to overcome low potency is to use the approved nononcology drugs as templates for the synthesis of new analogs or designed multiple ligands with optimized anticancer activities.^20–25^

In this study, we disclosed that Cle could be repurposed as a template for the design of agents targeting Fe^2+^-dependent histone lysine demethylases (KDMs) and Zn^2+^-dependent histone deacetylases (HDACs), two epigenetic-modifying proteins that are being actively investigated as anticancer drug targets. We observed that a cohort of the resulting KDM and HDAC inhibitors elicit selective antiproliferative effects against triple negative breast cancer (TNBC) and liver cancer cell lines. Moreover, a representative compound, **Cle-C8K,** selectively accumulates in liver tissues.

## 2 Results and Discussion

### 2.1 Clemizole-Based KDMs and HDACs Inhibitors: Design Rationale and Synthesis

KDMs and HDACs are families of epigenetic proteins that are involved in the cellular regulation of chromatin structure and gene expression. The KDMs that have been identified to date are grouped into two classes, namely, flavin-dependent monoamine oxidases, and Fe^2+^- and α-ketoglutarate-dependent Jumonji C (JmjC) domain-containing proteins. While there are only two homologues of flavin-dependent KDMs – KDM1A (LSD1) and KDM1B (LSD2) – in the human genome, the Fe^2+^- and α-ketoglutarate-dependent KDMs are much larger, and subdivided into six groups (KDMs 2–7).^26–30^ KDMs 1A and 1B facilitate the demethylation of monomethylated and dimethylated histone lysine residues, while KDMs 2–7 could act as demethylases for monomethylated, dimethylated, and trimethylated histone lysine residues.^28,30,31^ On the other hand, 18 HDAC isoforms have been identified to date. These HDACs promote the deacetylation of their histone and nonhistone protein targets using Zn^2+^-dependent (classes I, II and IV HDACs) and NAD^+^-dependent (class III HDACs or Sirtuins) mechanisms.^32,33^

Upregulation of the activities of KDMs and HDACs sustains the viability of several cancer types. In fact, pharmacological inhibitions of the enzymatic activities of KDMs and HDACs have demonstrated promising antiproliferative and cancer cell apoptosis-inducing effects. Five HDAC inhibitors (HDACi) have been approved for hematological cancers while several KDM inhibitors (KDMi) are in clinical trials.^33–35^ With few exceptions,^23^ the current KDMi and HDACi are systemic agents that indiscriminately alter the epigenetic landscape within cells, resulting in dose limiting toxic side effects. Strategies that enable tissue selective delivery of KDMi and HDACi could furnish a new generation of this class of epigenetic-modifying agents with improved therapeutic indices.^33^

For several metal ion-dependent KDMs and HDACs whose structures have been solved, the organization of the active sites follow a similar topology in which the metal ions are situated at the base of a pocket lined by narrow tunnels that lead to much wider solvent exposed outer rims. We postulated that this similar active site topology makes KDMi fit the same common molecular architecture as HDACi, comprising of a metal binding group (MBG) for chelation of the active site metal ions, a linker moiety for optimal presentation of the MBG and a surface recognition cap group accommodated within the enzymes’ outer rim. We have confirmed our postulation with the discovery of deferiprone (DFP)-derived KDMi that inhibit a cohort of Fe^2+^- and α-ketoglutarate-dependent KDMs.^36^ Because of the relatively large size of the enzyme’s outer rim in which it is accommodated, the surface recognition cap group could tolerate diverse modifications. Accordingly, moieties that impart new properties, including binding to other cancer-relevant targets, and tumor and/or tissue selectivity, have been integrated into the surface recognition cap group.^20–22,37–40^

Based on this precedence, we envisioned that the integration of Cle into the surface recognition cap group could furnish KDMi and HDACi (Figure 1) which retain the liver tissue selective accumulation of the template Cle while eliciting enhanced potency. Such liver tissue accumulating epigenetic modifiers could be novel liver cancer therapeutic agents.

**Figure 1:**
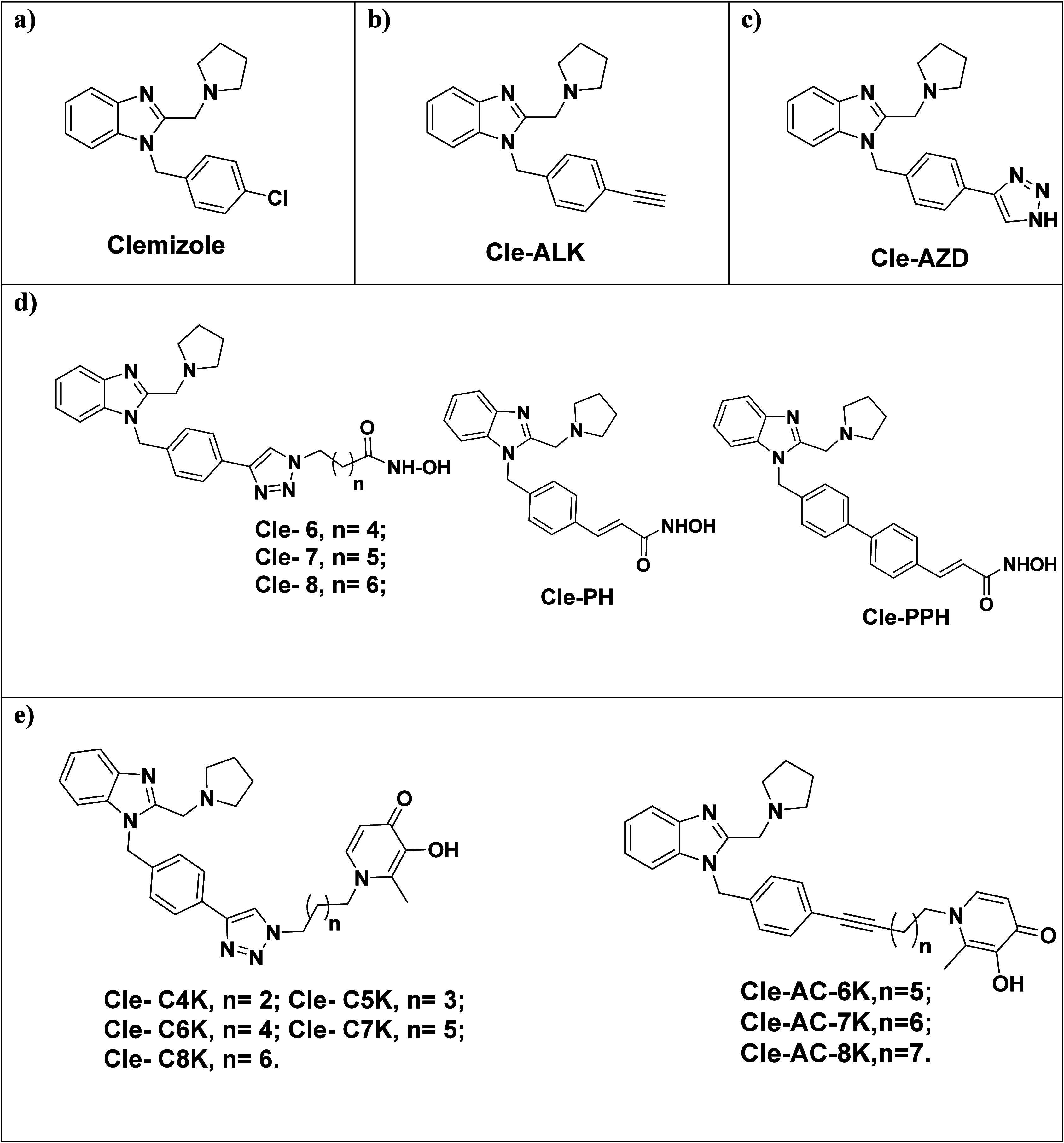
Design of the Cle-derived HDACi and KDMi. Structures of Cle (a), control compounds Cle-ALK (b), Cle-AZD (c), Cle-HDACi, (d) and Cle-KDMi (e).

In designing the desired Cle-HDACi and Cle-KDMi, Cle is integrated into the surface recognition cap groups of HDACi and KDMi by replacing its chloro substituent with the representative linker groups that connect to hydroxamate- and DFP-derived MBG, respectively. The choice of the linkers we used is informed by our previous studies where we identified the ideal and optimal linker lengths.^36,41^ To validate our design, we used molecular docking analysis (Autodock Vina)^42^ to evaluate the docked poses of representative HDACi compounds **Cle-6** and **Cle-PH** against HDAC2 (PDB: 3MAX) and HDAC6 (PDB: 5G0J) along with representative KDMi compounds **Cle-4CK**, **Cle-8CK**, and **Cle-AC-8K** against KDM6A (PDB: 3AVR) and KDM5B­(PDB: 6H4Z) as we have previously described.^22,36^ The quality of the docked outputs was evaluated based on orientation within the active sites and dock scores (Figure 2 and Suppl Info Table S1). We observed these compounds adopted poses in which their surface group (the Cle moiety) is oriented toward each enzyme’s outer rim while their MBGs chelate the metal ion at the active site of each enzyme (Figure 2A-D). In addition, we observed other interactions that may contribute to the accommodation of the compounds within the active sites of the enzymes.

**Figure 2:**
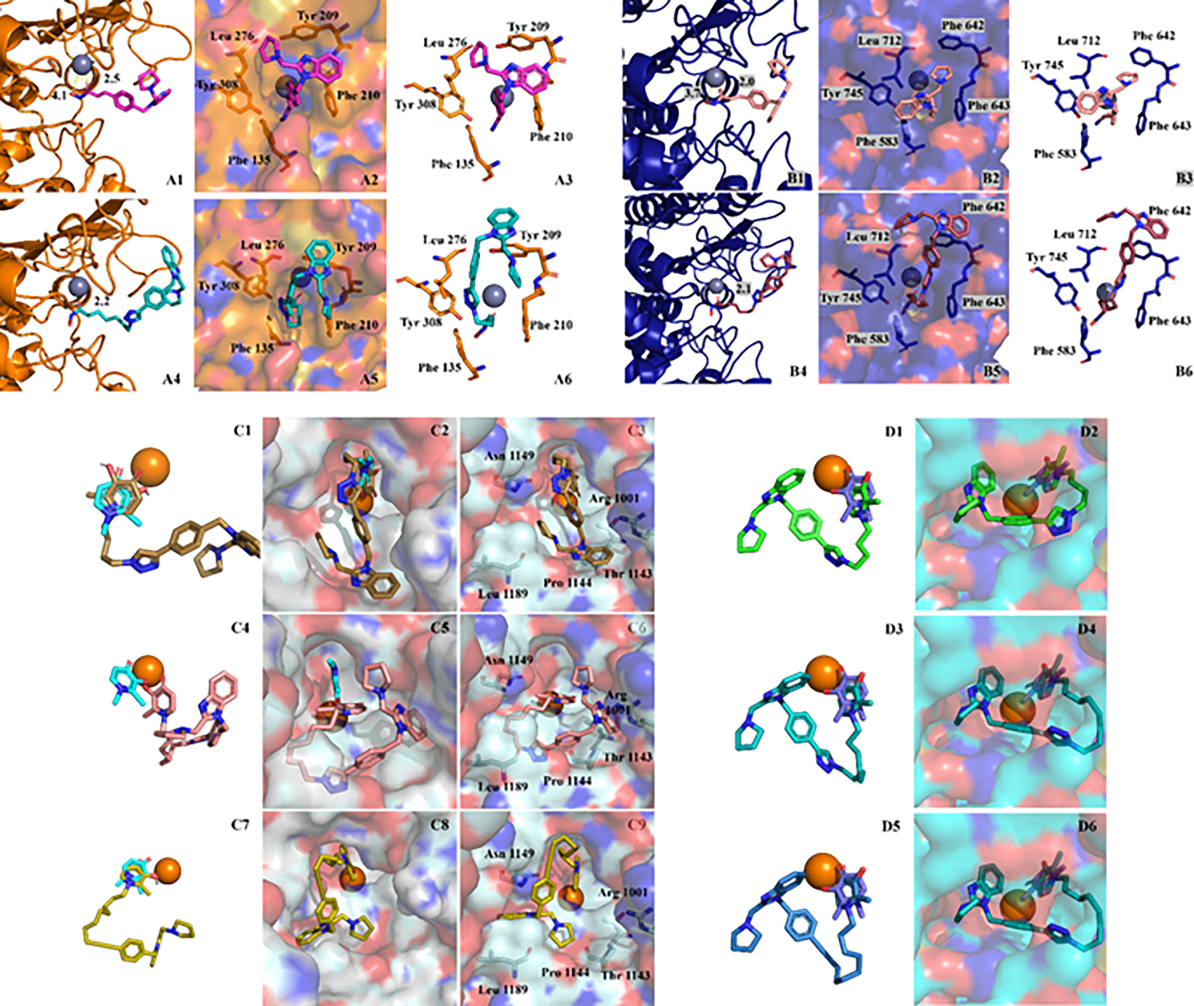
Representative docked poses of **Cle-C6** and **Cle-PH** within HDAC2 (PDB: 3MAX) (A) and HDAC6 (PDB: 5G0J) (B) active sites; and **Cle-4CK**, **Cle-8CK**, and **Cle-AC-8K** within KDM6A (PDB: 3AVR) (C) and KDM5B (PDB: 6H4Z) (D) active sites showing metal ion chelation and accommodation within the enzymes’ outer rims.

For docking to HDAC2, the Cle moiety of **Cle-C6** and **Cle-PH** seems to interact with PHE 210 and TYR 209 on the surface of the enzyme, while due to the shorter linker length, **Cle-PH** interacts with PHE 155 in the active site tunnel (Figure 2A). For docking to HDAC6, the Cle moiety of **Cle-C6** and **Cle-PH** seems to interact with PHE 642 and TYR 712 on the surface of the enzyme, again due to its shorter linker length, **Cle-PH** engages in π-stacking interactions with PHE 643 and PHE 583 in the active site tunnel (Figure 2B). The additional π-stacking interactions with the PHEs in the active site tunnels is reflected in the binding score as **Cle-PH** has binding scores of −8.4 and −10.4 kcal/mol for HDAC2 and HDAC6 respectively, while the binding scores for **Cle-C6** are −6.6 and −7.2 kcal/mol for HDAC2 and HDAC6.

For the docking with KDMs 5B and 6A, the KDMi adopt docked poses showing Fe chelation, however, it is less clear if their Cle moiety is interacting directly with the enzymes’ surface, although it is nevertheless tolerated (Figure 2C–D). Based on the results from the molecular docking analyses, it appears that the Cle moiety will be accommodated at the outer rims of HDACs 2 and 6, as well as KDM6A, and KDM5B, possibly through a combination of metal ion chelation provided by their MBGs and stabilizing interactions with the outer rim residues, particularly for the HDACi.

### Chemistry

The synthesis of the designed Cle-derived HDACi and KDMi went through the intermediacy of compounds **3** and **Cle-ALK** (**5**). Shown in Scheme 1 is the synthetic route to these common intermediates. Briefly, the reaction of benzene-1,2-diamine and ethyl 2-chloroacetate within 4 h in 5 M HCl followed by quenching with 1 M NH_4_OH resulted in the precipitation of 2-(chloromethyl)-1H-benzo­[d]­imidazole (**1**) which was isolated by filtration. The reaction of compound **1** with pyrrolidine in ethanol under refluxing conditions for 24 h furnished compound **2** which was subsequently reacted with bromobenzyl bromide in DMF to afford the requisite intermediate compound **3**. Sonogashira reaction^43^ of **3** with TMS-acetylene afforded compound **4** which was deprotected with TBAF to furnish the second requisite intermediate compound **5.**

**Scheme 1:**
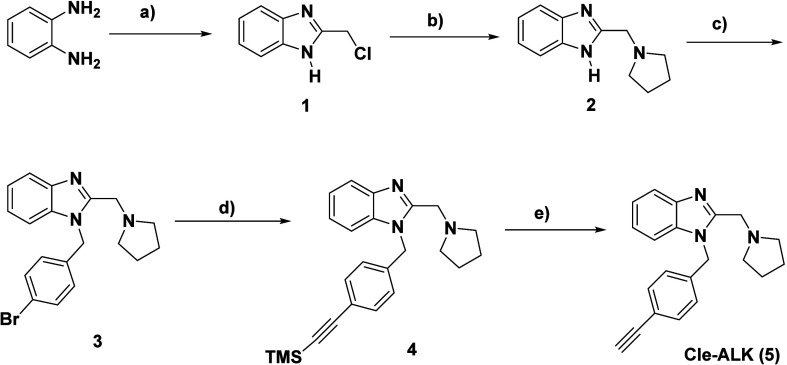
Synthesis of the Intermediate Compounds **3** and **Cle-ALK** (**5**)­^a^

a Reagents and conditions: (i) ethyl 2-chloroacetate, 5 M HCl, (ii) NH_4_OH; (b) pyrrolidine, ethanol, reflux, 24 h; (c) 4-bromobenzyl bromide, NaH, DMF, 0 °C to rt, 24 h, (d) TMS acetylene, Pd (PPh_3_)_4_, CuI, DIPEA, CH_3_CN, 83 ° C, 15 h; (e) TBAF, THF, 0°C to rt, 1 h.

The synthesis of all designed Cle-derived HDACi was accomplished as enumerated in Scheme 2. Cu (I)-mediated cycloaddition reaction of the previously disclosed *O*-trityl protected azido hydroxamates **6a**–**c** ^38^ with compound **5** furnished the protected penultimate intermediates **7a**–**c** which were deprotected with TFA to give the desired HDACi (**Cle-C6**, **Cle-C7**, and **Cle-C8**). The synthesis of the next compound begins with Heck coupling^44^ of *O*-trityl protected acrylyl hydroxamate **8** with compound **3** resulted in the protected intermediate compound **9** which was subjected to TFA deprotection to give the desired compound **Cle-PH**. Suzuki coupling of 4-hydroxyphenylboronic acid with **3** gave phenol compound **10** which was converted to triflate **11** by reaction with triflic anhydride. Heck coupling of *O*-trityl protected acrylyl hydroxamate with triflate **11** gave the protected intermediate compound **12** which was subjected to TFA deprotection to give the desired compound **Cle-PPH.**

**Scheme 2:**
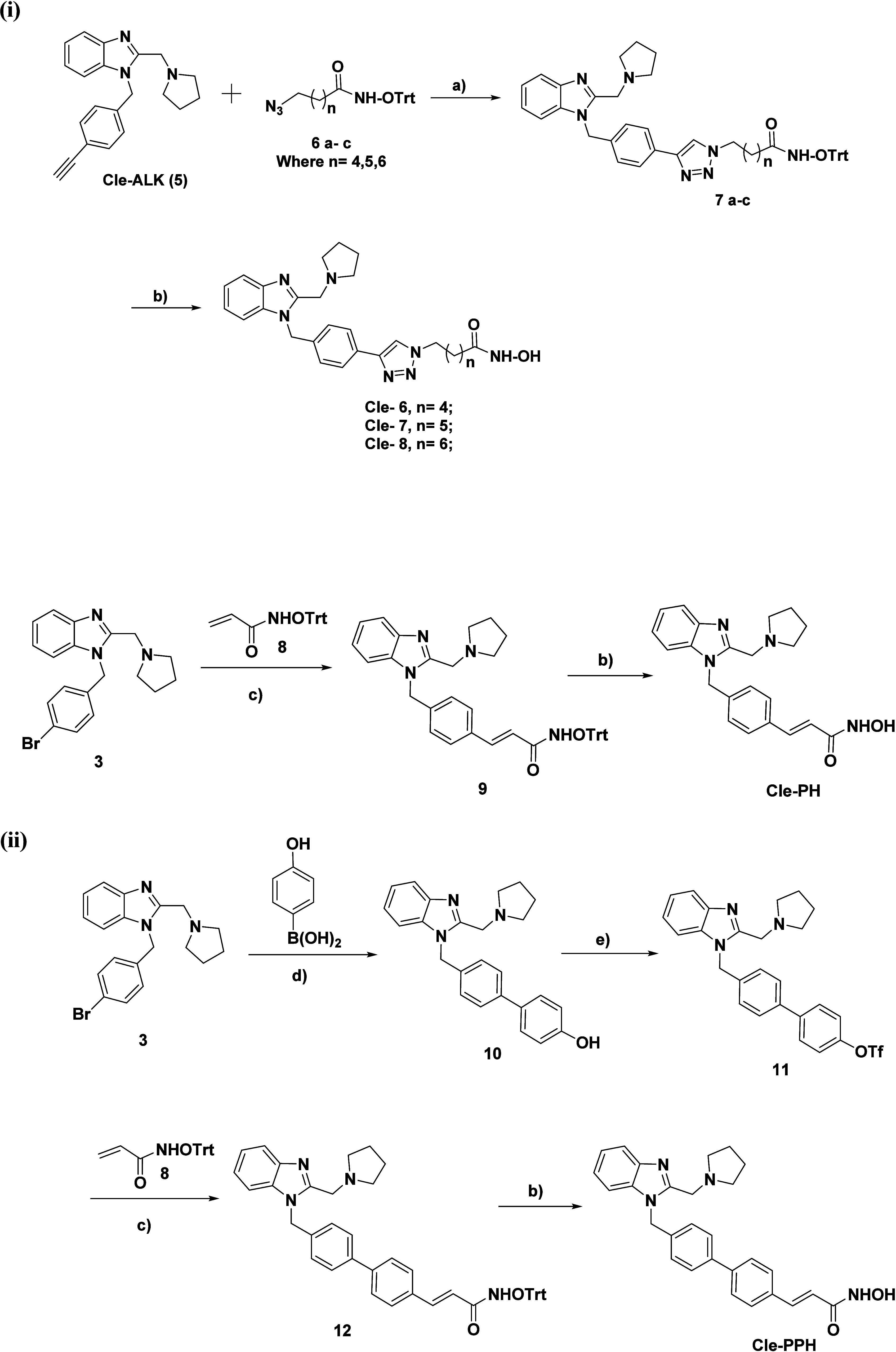
Synthesis of the designed Cle-derived HDACi^a^

a Reagents and conditions: (a) CuI, DIPEA, THF, rt, 15 h; (b) TFA, THF, rt, 1 h; (c) Pd­(PPh_3_)_4_, Cs_2_CO_3_, DMF, 120° C, 15 h; (d) Pd­(PPh_3_)_4_, Cs_2_CO_3_, 1,4 dioxane: H_2_O, 110°C, 4 h; (e) triflic anhydride, pyridine, CH_2_Cl_2_, 0°C to rt, 1 h.

The synthesis of the Cle derived KDMi is accomplished as shown in Scheme 3. Cu (I)-mediated cycloaddition reaction of benzyl protected azido maltols **13a**–**e** ^36^ with compound **5** furnished the protected penultimate intermediates **14a**–**e** which were deprotected with concentrated HCl to give the desired triazole series of Cle-KDMi (**Cle-C4K**, **Cle-C5K**, **Cle-C6K**, **Cle-C7K**, and **Cle-C8K**). The synthesis of the alkynyl KDMi begins with Sonogashira coupling of compound **3** with PMB protected alkynyl maltols **15 a-c** gave the protected intermediate compounds **16 a-c** which were deprotected by treatment with TFA to give the desired compounds **Cle-AC6K**, **Cle-AC7K**, and **Cle-AC8K.**

**Scheme 3:**
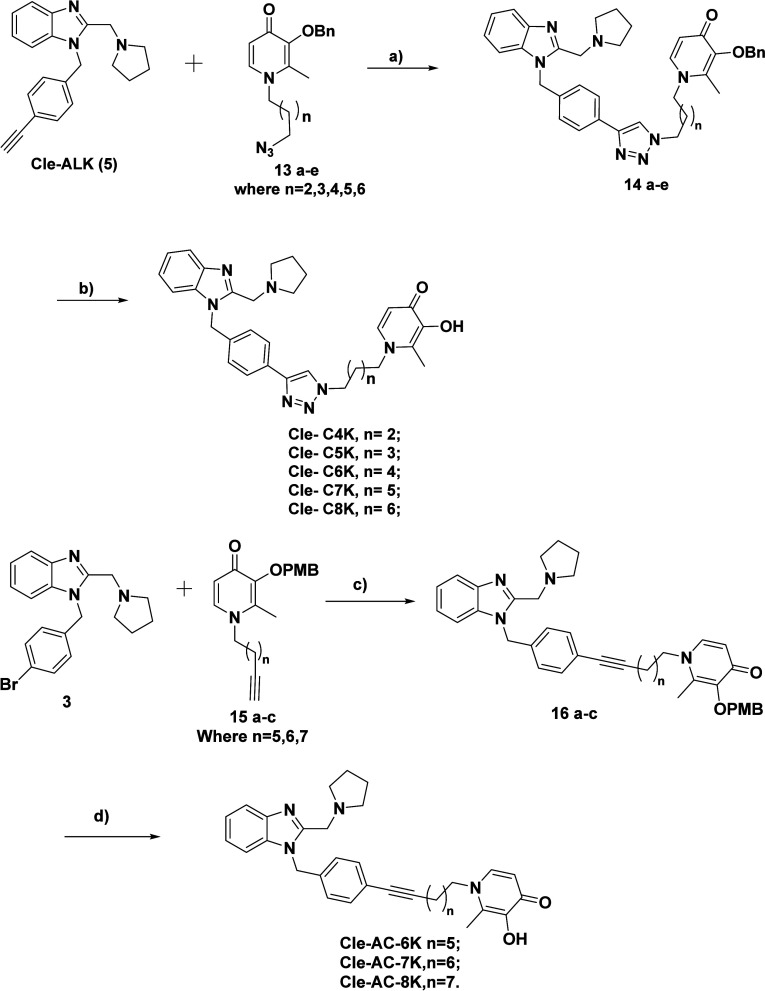
Synthesis of the designed Cle-derived KDMi^a^

a Reagents and conditions: (a) CuI, DIPEA, THF, rt, 15 h; (b) conc. HCl, rt, 24 h; (c) Pd­(PPh_3_)_4_, CuI, DIPEA, CH_3_CN, 83°C, 15 h. (d) TFA, DCM, 0°C to rt, 1 h.

### 2.2 Target Validation: HDAC Inhibition Study

The designed Cle-derived HDACi were screened against HDAC isoforms 1, 2, 6, and 8 in a cell-free assay (BPS Bioscience, San Diego, CA). We observed that these compounds elicited isoform-dependent HDAC inhibitory activities with IC_50_ values ranging from single digit nanomolar to high micromolar (Table 1). Specifically, these compounds inhibited HDAC8 the least, with high micromolar IC_50_s except **Cle-C7** which inhibited HDAC8 with a midnanomolar IC_50_. In contrast, they all inhibited HDAC6 most potently with the least potent compound, cinnamate **Cle-PPH**, having an IC_50_ value of 373 nM. Additionally, **Cle-PPH** showed very weak inhibition of class I HDAC isoforms 1 and 2, while a closely related compound, **Cle-PH**, is relatively more potent against HDACs 1 and 2. The methylene-linked compounds **Cle-C6**, **Cle-C7** and **Cle-C8** showed broad inhibition of HDACs 1 and 2 with the 6-methylene linker compound, **Cle-C7**, being the most potent. The pattern of the HDAC inhibition activity of the triazole-linked compounds **Cle-C6**, **Cle-C7**, and **Cle-C8**, against HDAC2 and HDAC6, is in close agreement with their dock scores (Supplementary Table S1). However, the trend of the HDAC inhibition activity of **Cle-PH** and **Cle-PPH** does not fit into a similar trend seen with the triazole-linked compounds, as they both have substantially lower docking scores for HDAC2 −8.9 and −9.4 kcal/mol and HDAC6 −10.4 and −7.2 kcal/mol respectively, while having higher IC_50_s, when compared to that of **Cle-C7**, against HDAC1, HDAC2, and HDAC6 (Table 1).

**Table 1:** Cell-Free HDAC Inhibitory Activities (nM) of the Cle-Derived HDACi

compounds	HDAC1	HDAC2	HDAC6	HDAC8
**Cle-C6**	219	475	2.33	1300
**Cle-C7**	43.3	137	1.53	339
**Cle-C8**	392	732	11.8	1520
**Cle-PH**	558	1333	20.2	1730
**Cle-PPH**	8130	12900	373	2940
TSA	3.56	10.1	1.70	620

Overall, this HDAC inhibitory data demonstrated that Cle is tolerated at the surface recognition cap groups of HDACi and possibly KDMi, by being oriented within the large outer rims of these enzymes.

To probe the validity of the HDAC cell-free inhibition data, we used Western blotting to investigate the effects of representative Cle-derived HDACi on the acetylation status of α-tubulin and histone H4 (H4), markers of intracellular inhibition of HDAC6 and class I HDACs 1–3,^45–47^ respectively, in Hep-G2 cells. For this experiment, we selected **Cle-C7**, **Cle-C8**, **Cle-PH**, and **Cle-PPH** as representative compounds, and used SAHA as a control HDACi and GAPDH levels to control for protein loading. As expected, across the various treatments of Hep-G2 cells for 4 h, in comparison with the untreated/DMSO negative control, there is no significant difference (*p* > 0.05) in the expression levels of α-tubulin. However, the levels of acetylated α-tubulin are highly/significantly (*p* < 0.05) upregulated, in comparison with the negative control. Specifically, compounds **Cle-C7**, **Cle-C8** and **Cle-PH** exhibit higher upregulation of acetylated α-tubulin when compared to **Cle-PPH**. This trend agrees with their cell-free HDAC inhibitory activities. Additionally, in comparison with negative control, these compounds upregulated the status of acetylated histone H4, following a trend that paralleled their class I HDACs inhibitory activities. Furthermore, all the Cle-based HDACi upregulated the expression levels of p21 (Figure 3; Supplementary Figures S1 and S2). These observations suggest that these compounds holistically elicit intracellular class I HDACs 1–3 and HDAC6 inhibition activities and potentially also influence Hep-G2 cell cycle regulatory activities.

**Figure 3:**
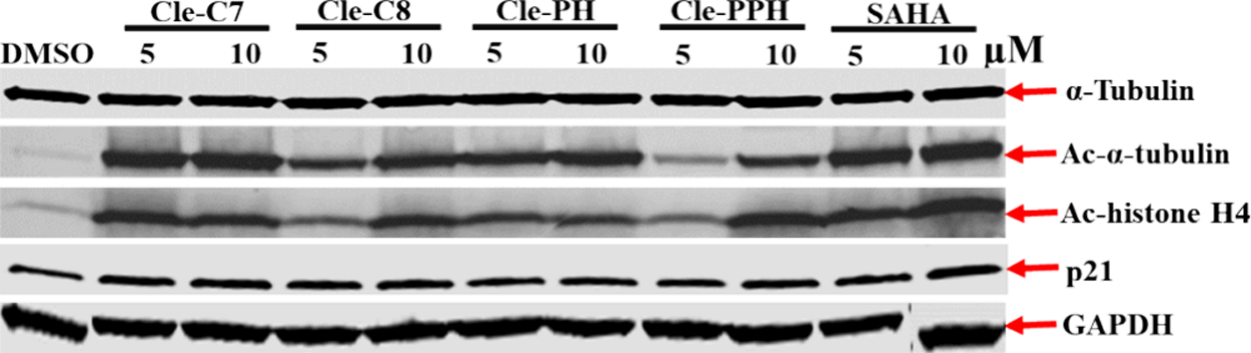
Western blot analysis of the effects of Cle-derived HDACi on α-tubulin and histone H4 acetylation status in Hep-G2 cells treated with 1% DMSO or 1% DMSO solutions of the test compounds: **Cle-C7** (5 and 10 μM), **Cle-C8** (5 and 10 μM), **Cle-PH** (5 and 10 μM), **Cle-PPH** (5 and 10 μM), and SAHA (5 and 10 μM), for 4 h. Representative gel bands are shown above.

### 2.3 Target Validation: KDM Inhibition Study

To investigate the effects of the Cle-derived KDMi on chromatin dynamics through intracellular KDM inhibition, we profiled them in a chromatin *in vivo* assay (CiA), which measures heterochromatin formation speed in mouse embryonic stem (mES) cells by leveraging chemical induced proximity (CIP) to recruit heterochromatin protein 1 (HP1) to a modified *Oct4* locus.^36^ Unlike cell-free assays, chromatin activity is a direct indicator of the demethylase activities of KDMs within the cell. As shown in Figure 4, all Cle-derived KDMi exhibited dose-dependent inhibition of HP1-induced heterochromatin formation within 48 h of exposure to CiA mES cells. Specifically, alkynyl compounds **Cle-AC6K**, **Cle-AC7K** and **Cle-AC8K** exhibited maximal inhibition of heterochromatin formation at high nanomolar concentrations. Among the triazolyl compounds, however, only **Cle-C7K** and **Cle-C8K** displayed maximum inhibition of heterochromatin formation at approximately 2.5 μM. In contrast, **Cle-C6K** inhibited heterochromatin formation by about 70% at 2.5 μM, while **Cle-C5K** and **Cle-C4K**, compounds with relatively shorter methylene linkers, showed less than 50% inhibition at this concentration with **Cle-C4K** being least effective, exhibiting an inhibitory effect that is weaker than that of a previously disclosed control compound **VK-II-100** (Figure S3). This data strongly suggests that the Cle-derived KDMi possess intracellular KDM inhibitory activity that is linker-length dependent, an observation that is also in agreement with our *in silico* predictions; thus, validating our design.

**Figure 4:**
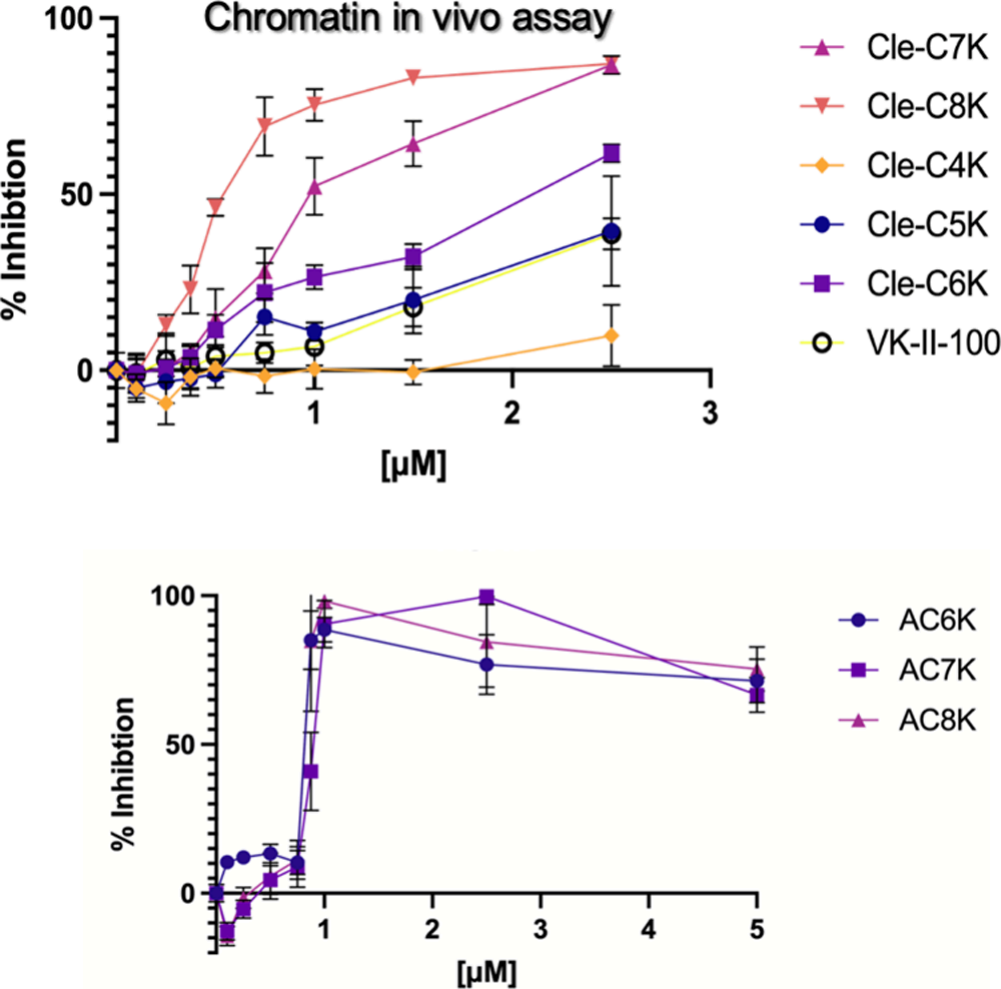
Chromatin *in vivo* assay (CiA) in mES cells treated with the test compounds for 48 h revealed dose dependent inhibition of HP1-induced heterochromatin formation by **Cle-C4K**, **Cle-C5K**, **Cle-C5K**, **Cle-C7K**, **Cle-C8K**, and **VK-II-100** (a) and **Cle-AC6K**, **Cle-AC7K**, and **Cle-AC8K** (b). Error bars represent the standard deviation of three biological replicates.

### 2.4 Cle-Derived HDACi and KDMi Are Cytotoxic to Cancer Cells

To investigate the effects of the Cle-derived HDACi and KDMi on cell proliferation, we screened them against a panel of cancer cells and a nontransformed cell line, using SAHA (approved HDACi), Cle and Cle analogs **Cle-AZD** and **Cle-ALK** as controls. The cancer cell lines that we selected are liver cancer cell lines Hep-G2, Huh7 and SK-HEP-1; lung cancer cell line A549; breast cancer cell lines MDA-MB-231, MCF-7; and prostate cancer cell lines DU145 and LNCaP while Vero, a kidney epithelial cell line, is included as the nontransformed cell line. We selected these cancer cell lines primarily based on the established roles of epigenetic dysfunctions in their sustenance and progression.^36,48–56^ Cells were treated with 0.1% DMSO solution of each compound for 72 h and cell viability was determined using MTS assay as we described previously.^21,22,36^

We observed that Cle and the control compounds **Cle-AZD** and **Cle-ALK** have nearly identical antiproliferative effects with high micromolar IC_50_s against the tested cells (Table 2). This data demonstrates that the replacement of the Cle’s chlorine group with a triazolyl or terminal alkynyl group neither compromised nor enhanced the anticancer activity of the resulting Cle analogs **Cle-AZD** and **Cle-ALK**. Interestingly, the Cle-derived HDACi displayed enhanced cytotoxicity with compound- and cell line-dependent potency (Table 2). In general, the TNBC cell line, MDA-MB-231, is most sensitive to these Cle-derived HDACi with single digit micromolar IC_50_s and potency enhancement of 15–61 folds relative to Cle. Furthermore, the methylene linker compounds **Cle-C6**, **Cle-C7** and **Cle-C8** are less toxic to A549, MCF-7, DU145 (except **Cle-C7**), Huh7, SK-HEP-1, LNCaP and Vero cells. Among these compounds, **Cle-C7** is the most selectively cytotoxic, inhibiting the proliferation of Hep-G2, MDA-MB-231, and DU145 with 4.1 μM, 1.8 μM and 5.0 μM IC_50_, respectively. Despite their considerably weaker HDAC inhibitory activities, **Cle-PH** and **Cle-PPH** are broadly toxic to the cell lines tested, including the nontransformed Vero cells (Table 2). This data suggests that **Cle-PH** and **Cle-PPH** may derive their cell cytotoxicity from the perturbation of additional cellular target(s).

**Table 2:** Anti-Proliferative Activity of Cle-Derived HDACi (IC_50_ in μM)^a^

HDACi	Hep-G2	A549	MDA-MB-231	MCF-7	VERO	Huh7	SK-HEP-1	DU145	LNCaP
Cle-C6	24.5 ± 0.4	65	6.34	NI	NI	NI	NI	33.13	65.7
Cle-C7	4.1 ± 0.4	11.41	1.8 ± 0.5	83.0 ± 16.8	NI	77.4 ± 5.6	30.3 ± 3.9	5.02	15.7
Cle-C8	14.3 ± 3.6	NI	5.9 ± 0.1	NI	NI	NI	78.7 ± 19.1	25.6 ± 5.7	46.2 ± 1.0
Cle-PH	4.8 ± 0.2	21.4	7.2 ± 0.6	19.1	5.6 ± 1.3	10.23	14.90	12.11	3.2
Cle-PPH	5.0 ± 0.1	12.87	3.9 ± 0.5	4.8 ± 3.2	4.1 ± 1.1	1.0 ± 0.02	3.2 ± 0.5	12.8 ± 0.8	3.2
Clemizole	38.0 ± 10.0	NT	108.9	68.9 ± 11.0	112.6 ± 22.3	27.8 ± 7.1	41.7	117.9	54.3 ± 9.2
Cle-AZD	44.8	NT	>100	NT	>100	63.0	>100	NT	NI
Cle-ALK	NT	NT	52.5 ± 3.0	76.5 ± 20	64.5 ± 13.2	NT	NT	NT	61.05

a NT = Not tested; NI = less than 50% inhibition at 100 μM.

Among the tested cell lines, the KDMi compounds are least cytotoxic to A549, MCF-7, Vero, DU145 and to a lesser extent, LNCaP cells. In similar manner to the Cle-derived HDACi, these KDMi compounds are potently cytotoxic to the MDA-MB-231 cells. Unlike the Cle-derived HDACi, however, the KDMi are broadly cytotoxic to all liver cancer cell lines that we tested. The exceptions are **Cle-C4K** and **Cle-C5K**, and **Cle-C7K**, which showed moderate cytotoxicity against Hep-G2 and SK-HEP-1 cells respectively (Table 3). Among these Cle-derived KDMi, **Cle-C7K**, **Cle-C8K**, **Cle-AC6K**, **Cle-AC7K** and **Cle-AC8K** are the most potent, with potency enhancement as high as 86- and 330-fold relative to Cle for **Cle-AC8K** against Hep-G2 and MDA-MB-231, respectively. In addition, across almost all the cell lines that we considered here, the alkynyl-based Cle-KDMi are more cytotoxic than their triazolyl-based counterparts. Summarily, this data demonstrates the potential utility of the Cle-derived KDMi in TNBC and liver cancer therapy.

**Table 3:** Anti-Proliferative Activity of Cle-Derived KDMi (IC_50_ in μM)

KDMi	Hep-G2	A549	MDA-MB-231	MCF-7	VERO	Huh7	SK-HEP-1	DU145	LNCaP
Cle-C4K	28.4 ± 2.0	>100	7.9 ± 1.0	70.9 ± 8.9	72.2 ± 3.5	4.1 ± 0.03	4.0	31.5 ± 0.8	20.2 ± 1.8
Cle-C5K	19.2 ± 1.9	>100	4.0 ± 1.2	43.9 ± 10.8	36.0 ± 2.8	9.1 ± 1.8	8.72	16.5 ± 2.4	12.3 ± 3.5
Cle-C6K	8.5 ± 0.9	64.1	1.7 ± 0.2	59.3 ± 6.6	22.4 ± 0.4	2.01 ± 0.01	8.74	12.2 ± 2.0	6.0 ± 0.6
Cle-C7K	3.2 ± 0.7	NT	1.5 ± 0.05	39.1 ± 6.7	9.6 ± 1.4	2.5 ± 0.05	25.40	12.8 ± 2.3	4.0 ± 1.5
Cle-C8K	6.1 ± 0.5	NT	0.95 ± 0.1	28.4 ± 0.3	7.9 ± 1.0	0.36 ± 0.1	8.0 ± 2.7	6.4 ± 0.03	4.0 ± 0.8
Cle-AC6K	0.36 ± 0.1	NT	0.65 ± 0.1	49.5 ± 0.1	5.85	0.7 ± 0.2	2.8 ± 0.1	2.2 ± 0.9	4.0 ± 2.0
Cle-AC7K	0.46 ± 0.3	NT	1.0 ± 0.1	47.2 ± 1.0	5.71	1.2 ± 0.1	3.0 ± 1.0	3.0 ± 1.4	3.3 ± 0.1
Cle-AC8K	0.44 ± 0.1	NT	0.33 ± 0.1	47.5 ± 2.1	2.01	0.65 ± 0.3	2.4 ± 0.4	3.7 ± 0.1	2.1 ± 0.1

### 2.5 Effects of Representative Cle-Derived HDACi and KDMi on Cell Cycle Progression

Based on their performance in the target validation and cell cytotoxicity assays, we selected **Cle-C7** (HDACi) and **Cle-C8K** and **Cle-AC8K** (KDMi) as candidates to investigate the effects of these Cle-derived compounds on cell cycle progression. We used Hep-G2 cells for this study. SAHA, an HDACi known to induce significant G2/M phase arrest in lung cancer,^57^ ovarian cancer^58^ and prostate cancer^59,60^ cells; and DFP, a pan-KDMi, were used as positive controls.^36^ In comparison with untreated control cells (Figure 5a), treatment of Hep-G2 cells with Cle (25 μM) resulted in a nonsignificant upregulation in cell population in S-phase (Figure 5b); however, on increasing the concentration of Cle to 50 μM, we observed an accumulation of cells in the G0/G1 phase of the cell cycle with an accompanying increase in apoptotic cell population (based on the < G1 population of cells; Figure 5c). Similarly, on increasing the concentration of **Cle-C7** from 5 to 10 μM, there is a remarkable increase in cell population in G2/M phase with a significant increase in apoptotic cells (Figure 5d-e). Furthermore, in agreement with previous reports, we observed that SAHA induced an upregulation of Hep-G2 cell population in G2/M phase after 24 h of treatment (Figure 5f; Supplementary Figure S4). These results demonstrate a similarity in the mode of action of **Cle-C7** and SAHA on cell cycle arrest and induction of apoptosis. Additionally, it appears that, while the Cle moiety of these compounds plays a role in their effects on cell cycle regulation, their HDAC inhibition activities play a more prominent role.

**Figure 5:**
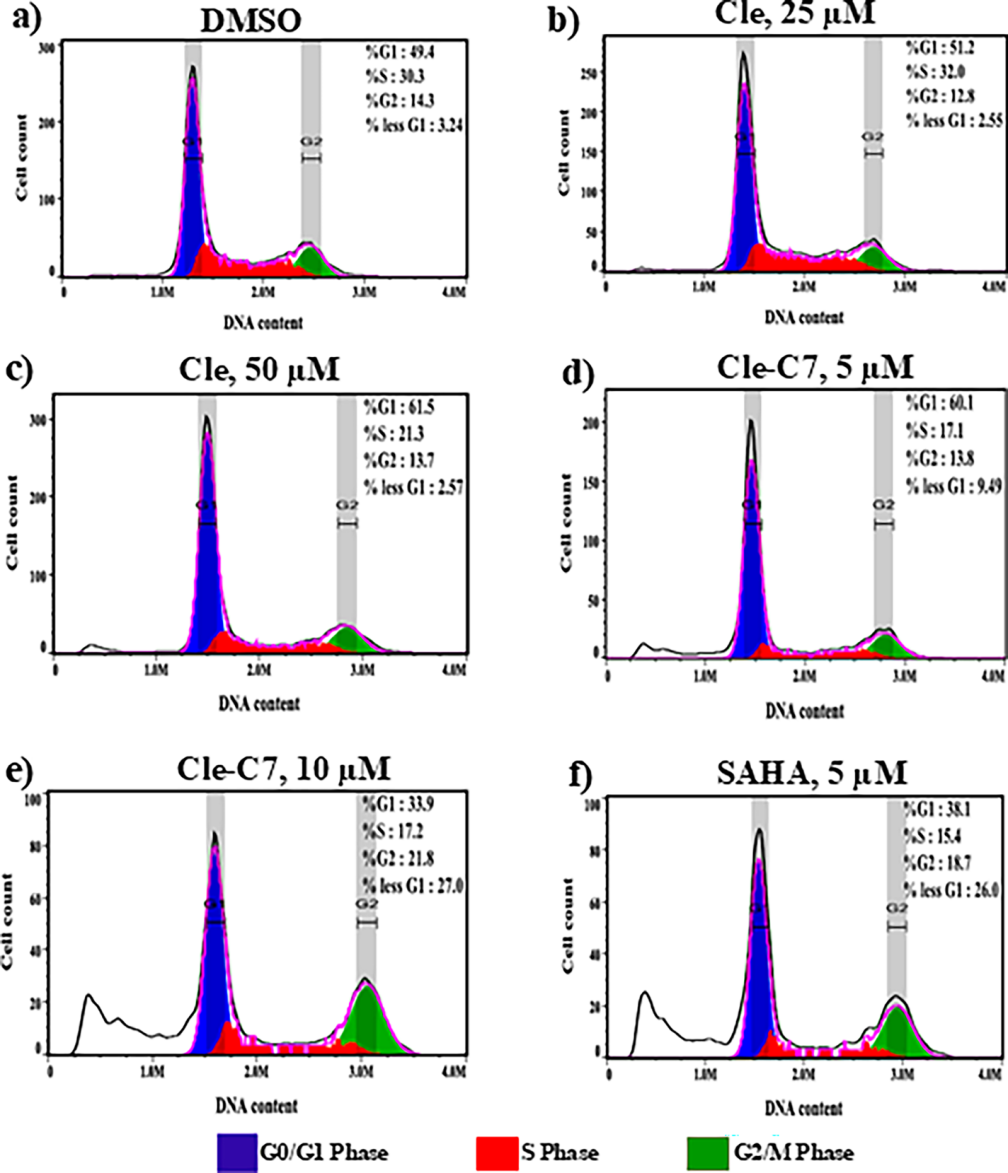
Effects of Cle-based HDACi on cell cycle progression in Hep-G2 cell line treated for 24 h. Histograms of negative control (a) and cells treated with the indicated compounds showing the distribution of cell populations in <G1, G0/G1, S, and G2/M phases of the cell cycle (b–f). These Cle-based HDACi induced apoptosis and an increase in cell population in G2/M-phases.

For the Cle-based KDMi, **Cle-C8K** and **Cle-AC8K** significantly (*p* < 0.05) triggered increase in S-phase cell population at both their IC_50_ and 2 × IC_50_ concentrations after 72 h of treatment. Also, these compounds demonstrated apoptosis-inducing effects (based on the < G1 population of cells) at both their respective concentrations of treatment, with **Cle-C8K** being more effective than **Cle-AC8K** (Figure 6a-e; Supplementary Figure S5). The enhanced apoptosis-inducing capability of **Cle-C8K** could be due to its higher capacity to induce S-phase cell cycle arrest relative to **Cle-AC8K**.^61^ DFP, the pan-KDMi from which the KDM inhibition moiety of these compounds was derived, has a similar effect on cell cycle progression (Figure 6f-g), inhibiting cell cycle progression in the S-phase in line with a previous report.^62^ Overall, these results established similarities between the Cle-based KDMi and DFP in terms of modulation of the cell cycle phases.

**Figure 6:**
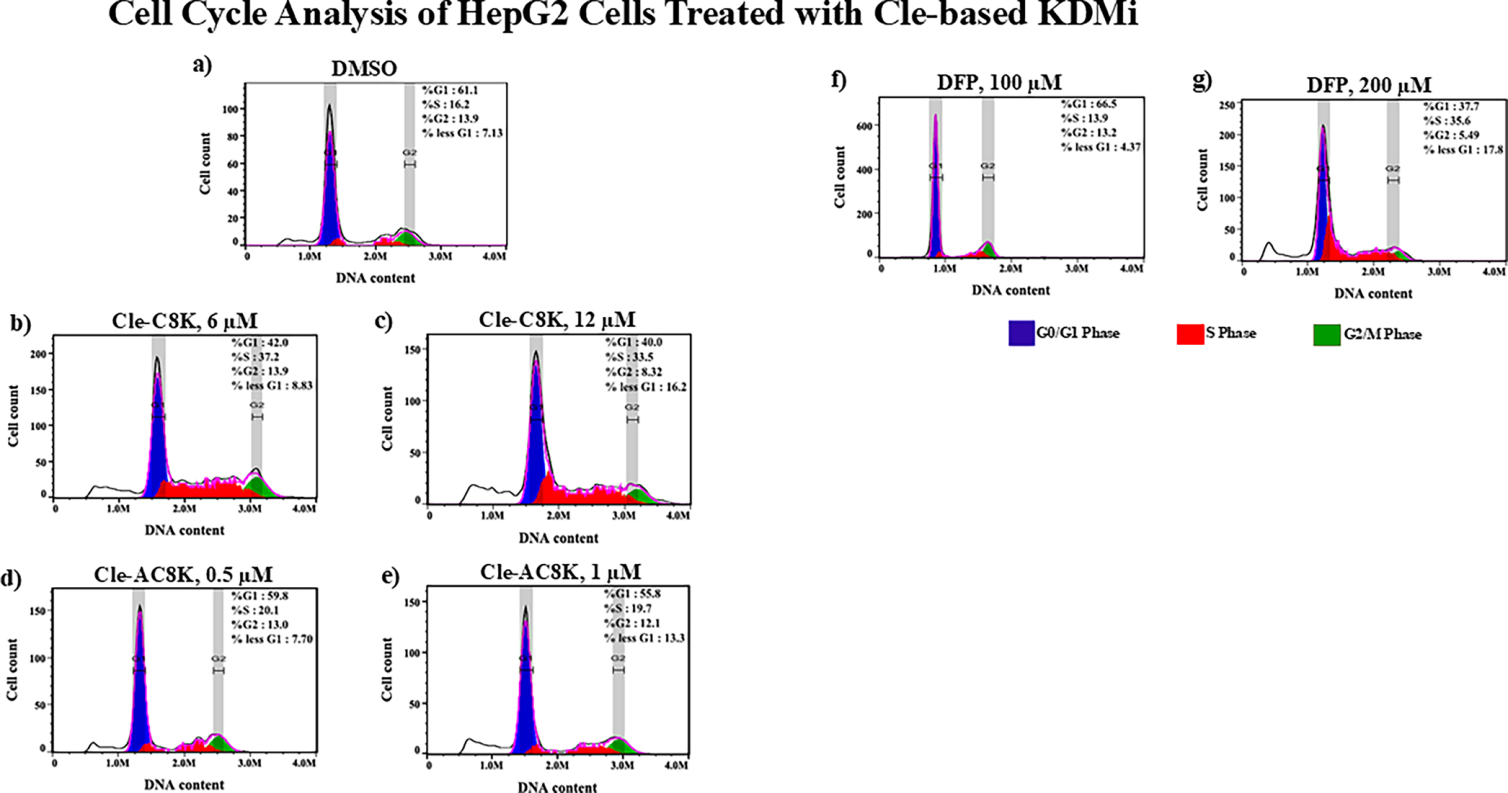
Effects of Cle-based KDMi and DFP on cell cycle progression in Hep-G2 cell line treated for 72 h. Histograms of negative control (a) and cells treated with the indicated compounds showing the distribution of cell populations in <G1, G0/G1, S, and G2/M phases of the cell cycle (b–g). These Cle-based KDMi and DFP induced apoptosis and cell cycle arrest in the S-phase.

### 2.7 Cle-Derived KDMi Modulate Cell Proliferation and Apoptosis-Associated Pathways

To further establish the mechanism(s) of the cytotoxic activity of the Cle-based KDMi, we used immunoblot assays to determine the effects of **Cle-C8K** and **Cle-AC8K** on the expression levels of selected target proteins/enzymes which are critical for the viabilities of several cancer cell lines, including Hep-G2 cells. Specifically, we probed for forkhead box protein M1 (FOXM1), cellular inhibitor of apoptosis protein 1 (cIAP1), cellular inhibitor of apoptosis protein 2 (cIAP2), X-linked inhibitor of apoptosis protein (XIAP), extracellular signal-regulated kinases 1/2 (ERK 1/2), Aurora kinase A (AURKA), cyclin-dependent kinase inhibitor 1 (CDKN1A/p21), androgen receptor (AR) and phosphorylated p38 mitogen-activated protein kinase (p-p38 MAPK) (Figure 7; Supplementary Figures S6 - 7). While the effect of treatment of Hep-G2 cells is not significant on the expression status of FOXMI, we observed a tendency for the downregulation of this signaling molecule by the Cle-based KDMi. Immunoblot results indicate that apoptosis-related pathways involving cIAP1 and XIAP are significantly downregulated, although we observed a compensatory response due to the upregulation of cIAP2.^63^ However, the downregulation of XIAP, being the most vital member of the IAPs, may be sufficient to nullify the compensatory effects of cIAP2.^64^ Similarly, the significant reductions in the expression status of cell proliferative targets, such as AR and ERK1/2,^65,66^ are further indicators of the pathways targeted by these Cle-based KDMi to inhibit the proliferation of HepG2 cells. The upregulation in AURKA may also be considered as a compensatory mechanism, to inhibit apoptosis considering the downregulation in the status of XIAP.^67^ Interestingly, we observed compound-dependent effects on the expression status of p21. Within the period of treatment, **Cle-AC8K** did not perturb p21 expression while **Cle-C8K** and DFP slightly downregulated p21 levels. The downregulation of p21 by **Cle-C8K** and DFP may distinctly contribute to the mechanisms of apoptosis induction by these compounds as indicated by the increase in apoptotic cell population, relative to **Cle-AC8K**, based on the cell cycle data in Figure 6.^68^ Moreover, there are several reports on the induction of apoptosis due to KDM inhibition.^69–71^ Finally, our immunoblot results also indicate an upregulation in p-p38 MAPK levels, which may be associated with the role of this signaling molecule in adaptation to an acute cellular stress by facilitating cell cycle arrest.^72^ From these results, it seems that **Cle-C8K** acts faster than **Cle-AC8K**; hence, the disparities observed in the various protein expression levels.

**Figure 7:**
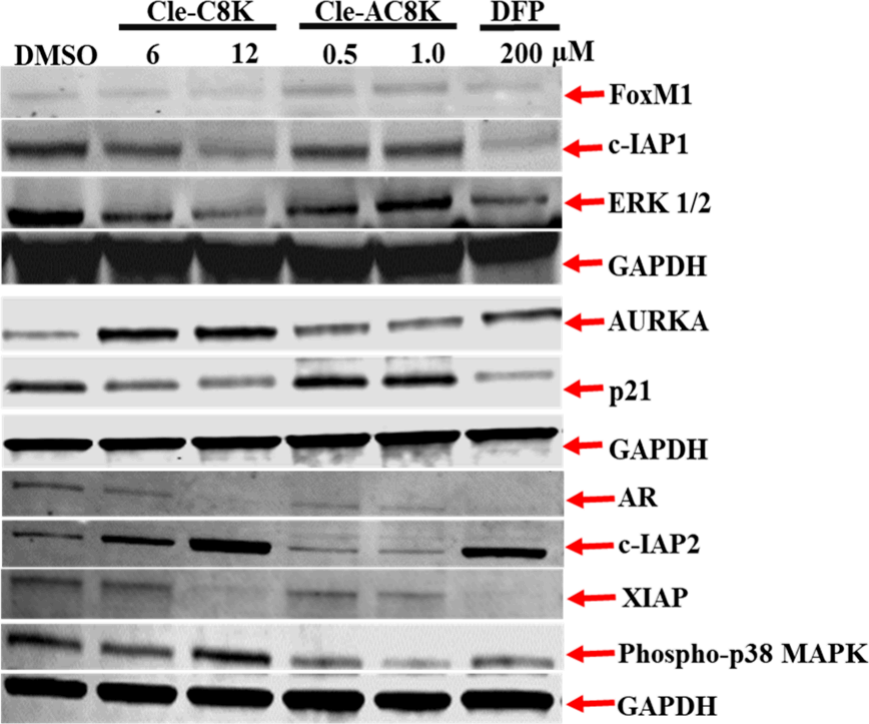
Western blot analysis of the expression of proteins involved in cell proliferation, cell motility, apoptosis and cell cycle regulation in Hep-G2 cells treated with **Cle-C8K**, **Cle-AC8K**, and DFP. Cells were treated with the tested agents for 72 h and total cell extracts were subsequently analyzed by immunoblot analysis. Representative gel bands are shown above. The respective densitometric quantifications are presented in the Supporting Information, Figures S6 and S7.

### 2.8 Analysis of the Effects of **Cle-C8K** on the Transcriptome in Huh7 Cells

RNA-sequencing analysis was used to further probe the transcriptome-level effects of **Cle-C8K** in Huh7 cells. Hallmark gene set enrichment analysis (GSEA) was conducted first using the human molecular signature database (MSigDB) hallmark collection composed of 50 gene sets. Gene sets determined to be significant (p-value <0.05, false discovery rate (FDR) < 0.25) were identified (Figure 8a).^73^ **Cle-C8K** at IC_50_ (0.5 μM), and 2 × IC_50_ (1 μM) negatively enriched G2M checkpoint (NES = −3.6, −3.3, respectively) (Figure 8b). Negative enrichment of the G2M checkpoint supports **Cle-C8K** induction of S phase arrest, in agreement with the cell cycle progression data in Figure 6. MYC targets v1 and v2 were negatively enriched by **Cle-C8K** at 0.5 μM (NES = −4.2, −3.7), and 1 μM (NES = −4.0, −3.6) (Figures 8c-d). E2F targets were negatively enriched by **Cle-C8K** at 0.5 μM (NES = −3.8), and 1 μM (NES = −4.1) (Figure 8e) while hypoxia was positively enriched by **Cle-C8K** at 0.5 μM (NES = +2.7) and 1 μM (NES = +3.3) (Figure 8f). Within the hypoxia gene set, tumor suppressors CDKN1A (log2 fold change +1.9, 0.5 μM and +2.4, 1 μM), FOXO3 (log2 fold change +1.0, 0.5 μM and +1.7, 1 μM), DDIT4 (log2 fold change +1.9, 0.5 μM and +2.4, 1 μM), and ZFP36 (log2 fold change +1.6, 0.5 μM and +2.1, 1 μM), and pro-apoptotic factor BNIP3L (log2 fold change +1.7, 0.5 μM and +2.2, 1 μM)^74–76^ were all upregulated by **Cle-C8K** (Supplementary Figure S8). A closely related compound, **Cle-C6K**, has a similar effect on this gene set (Supplementary Figure S9).

**Figure 8:**
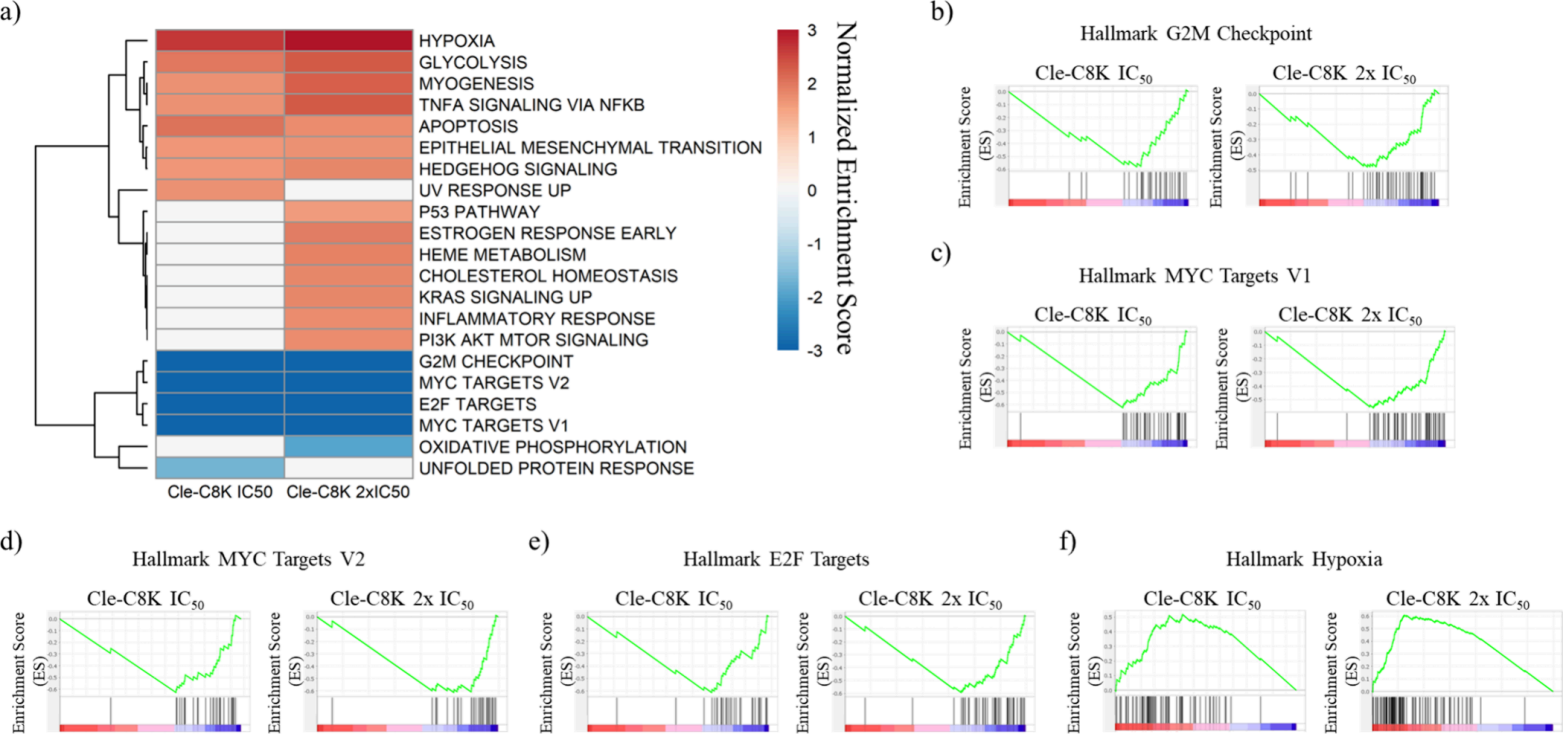
Hallmark gene set enrichment analysis (GSEA) in Huh7 cells treated with **Cle-C8K**. (a) Heatmap of normalized enrichment scores (NES) of significantly enriched hallmark gene sets resulting from treatment with **Cle-C8K** at 0.5 μM (IC_50_) and 1 μM (2 × IC_50_) (*p* < 0.05, FDR < 0.25). (b) G2M checkpoint enrichment plot for **Cle-C8K** at 0.5 μM (NES = −3.6) and 1 μM (NES = −3.3). (c) MYC targets v1 enrichment plot for **Cle-C8K** at 0.5 μM (NES = −4.2) and 1 μM (NES = −4.0). (d) MYC targets v2 enrichment plot for **Cle-C8K** at 0.5 μM (NES = −3.7) and 1 μM (NES = −3.6). (e) E2F targets enrichment plot for **Cle-C8K** at 0.5 μM (NES = −3.8) and 1 μM (NES = −4.1). (f) Hypoxia enrichment plot for **Cle-C8K** at 0.5 μM (NES = +2.7) and 1 μM (NES = +3.3).

The effects of **Cle-C8K** treatment on MSigDB gene ontology biological process (GOBP) gene sets were also analyzed and significant gene set perturbations (*p* < 0.01, FDR < 0.05) were identified (Supplementary Figure S10a).^77^ Gene sets encompassing ribosomal and RNA-related processes were negatively enriched by **Cle-C8K** at 0.5 μM and 1 μM including tRNA metabolic process, NES −3.3 (1 μM) (Figure 9a). **Cle-C6K** has a similar effect except that **Cle-C8K** significantly enriched more gene sets. Also, only **Cle-C6K** negatively enriched the regulation of post-translational protein modification, NES −2.2 (2.5 μM) and −2.1 (5 μM) (Supplementary Figure S10b). Among the chromosomal and DNA-related gene sets negatively enriched by **Cle-C8K** at 1 μM were DNA replication (NES = −3.1) and DNA recombination (NES = −3.3) (Figure 9b). Additionally, **Cle-C8K** positively enriched the response to oxygen levels gene set (NES = +2.8) in a concentration-dependent manner (Figure 9c).

**Figure 9:**
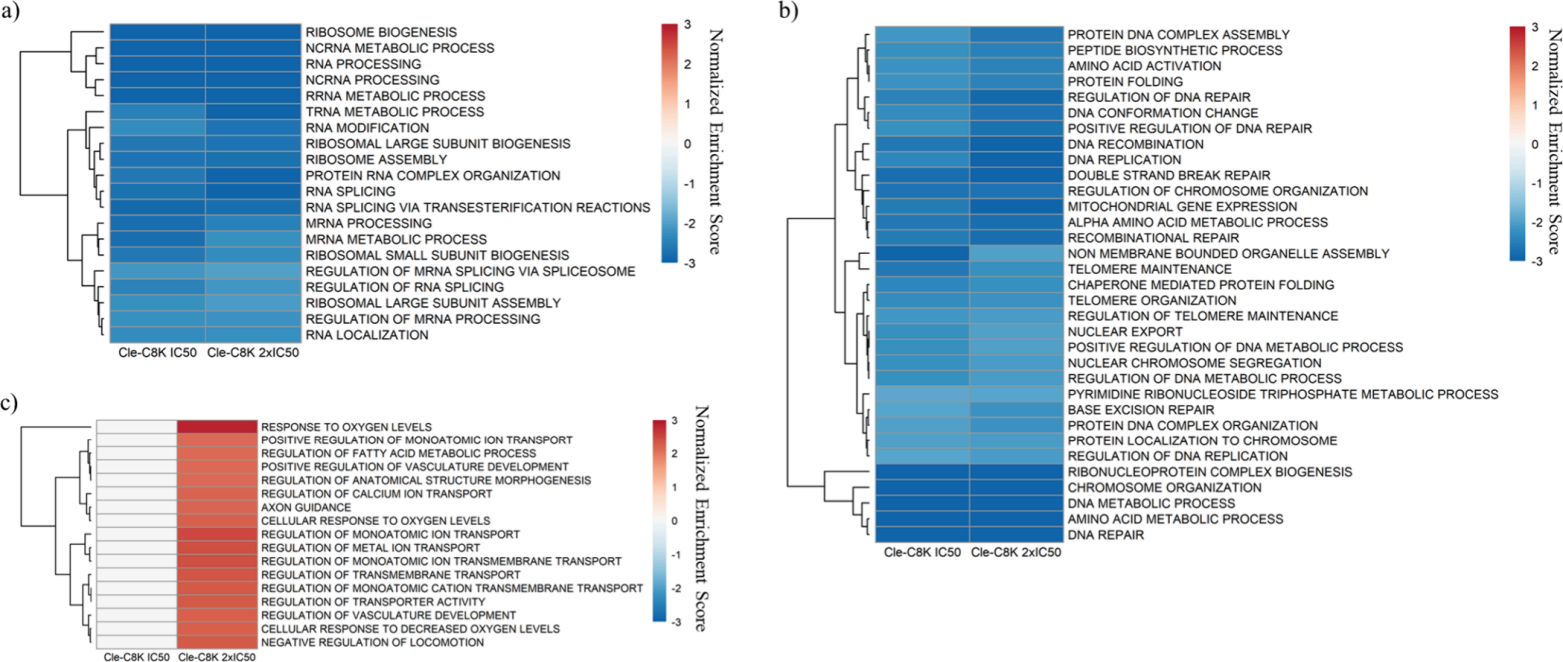
Gene Ontology biological processes (GOBP) gene set enrichment analysis (GSEA) in Huh7 cells treated with **Cle-C8K**. (a) Heatmap of normalized enrichment scores (NES) of ribosomal and RNA-related process gene sets significantly and negatively enriched by **Cle-C8K** treatment (*p* < 0.01, FDR < 0.05). (b) NES heatmap of chromosomal, DNA-related, and protein-related process gene sets significantly and negatively enriched by **Cle-C8K** at IC_50_ (0.5 μM) and 2 × IC_50_ (1 μM). (c) NES heatmap of gene sets significantly and positively enriched by **Cle-C8K** at 1 μM.

Moreover, **Cle-C8K** effect on a standard gene set including of oncogenes and cell cycle inhibitors revealed downregulation of oncogenes CDCA7 (log2 fold change −2.4, at 1 μM) and CCNB1 (log2 fold change −1.5, at 1 μM) (Figure 10). Cell cycle inhibitors CDKN1A (p21) (log2 fold change +2.4, at 1 μM), CDKN1C (log2 fold change +1.5, at 1 μM), and CDKN2D (log2 fold change +1.3, at 1 μM) were upregulated along with tumor suppressor FOXO4 (log2 fold change +1.7, at 1 μM) (Figure 10). **Cle-C6K** elicited a similar effect on these genes (Supplementary Figure S11).

**Figure 10:**
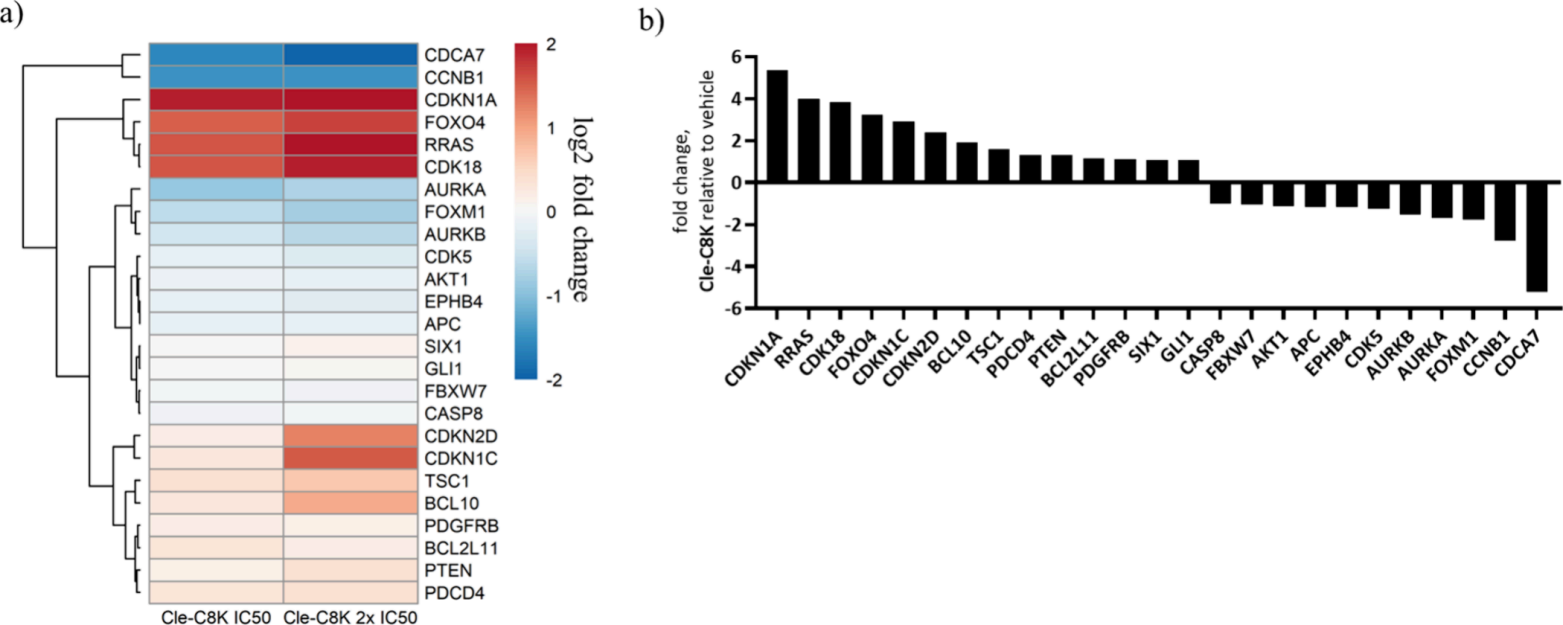
Effects of **Cle-C8K** on the expression of standard gene set of selected oncogenes, tumor suppressors, and cell cycle inhibitors. (a) Log2 fold change heatmap of genes implicated in the KDM inhibition activity displaying upregulation of CDKN1A (p21) by **Cle-C8K** at both IC_50_ (0.5 μM) and 2 × IC_50_ (1 μM) (log2 fold change = +1.9, 0.5 μM, and +2.4, 1 μM), downregulation of CCNB1 (log2 fold change = −1.5, 0.5 μM, and −1.5, 1 μM). b) Fold change bar graph of **Cle-C8K** at 1 μM relative to DMSO.

Lastly, although the **Cle-C8K**-induced upregulation of the p21 RNA does not agree with the slight downregulation of the p21 protein that we noticed in Western blotting (Figure 7), we nevertheless analyzed the RNA seq data for the effect of **Cle-C8K** on the other genes that we probed for in the Western blot data in Figure 7. We observed that similar to the Western blot results, **Cle-C8K** at 1 μM (2 × IC_50_) led to a 5.4-fold increase in cIAP2 expression (log2 fold change +3.8), and a decrease in XIAP (2.5-fold), AR (2.5-fold), ERK1 (2-fold) and ERK2 (3.3-fold) expression when normalized to GAPDH (log2 fold change +1.4) (Supplementary Figure S12). The effects of **Cle-C6K** on these genes are not as prominent (Supplementary Figure S13).

Collectively, this RNA seq data revealed that both **Cle-C6K** and **Cle-C8K** caused perturbations to the transcriptome that tilt the balance in favor of cycle progression inhibition and increased apoptosis, resulting in cancer cell death.

### 2.9 Maximum Dose Determination and Analysis of the Tissue Distribution of **Cle-C8K**

One of the appeals for repurposing Cle for liver cancer therapy is its demonstrated selective accumulation within the liver tissue. Although the foregoing data revealed that the Cle-derived epigenetic modifiers herein described, especially the Cle-KDMi, potently inhibit the proliferation of liver cancer cells, the retention of the liver tissue selective accumulation property of their parent Cle template could positively impact therapeutic potential. To preliminarily elucidate their tissue distribution, we selected **Cle-C8K**, being one of the Cle-KDMi that is cytotoxic to all liver cancer cells that we tested, as an exemplifying compound. To identify an optimum dose for the tissue distribution study, we first determined the maximum tolerated dose (MTD) of **Cle-C8K** using healthy C57BL/6 mice. MTD measurement was performed using male and female mice dosed at 25 mg/kg, 50 mg/kg and 100 mg/kg (n = 4 per group) via intraperitoneal (IP) route, continuously for 7 days as we described before.^23^ Based on body weight as an indicator of toxicity, we observed that **Cle-C8K** showed no adverse effect at all tested doses as it did not cause any significant weight loss (Figure 11a). Based on this data, we selected the 50 mg/kg dose to preliminarily investigate the tissue distribution of **Cle-C8K**. After 8 h of the last injection, the 50 mg/kg dose cohort mice were sacrificed and selected organs – liver, lungs, kidney, and plasma – were harvested. The levels of **Cle-C8K** in these organs were measured using mass spectrometric analysis. We observed that the level of **Cle-C8K** in the plasma is below the detection limit of our instrument while it is accumulated approximately 12- and 32-fold higher in the liver relative to the lung and the kidney respectively (Figure 11b, Supplementary Figure S14a-c). This preliminary result suggests that **Cle-C8K** is a relative nontoxic, liver tissue accumulating KDMi that potently inhibits the proliferation of liver cancer cells.

**Figure 11:**
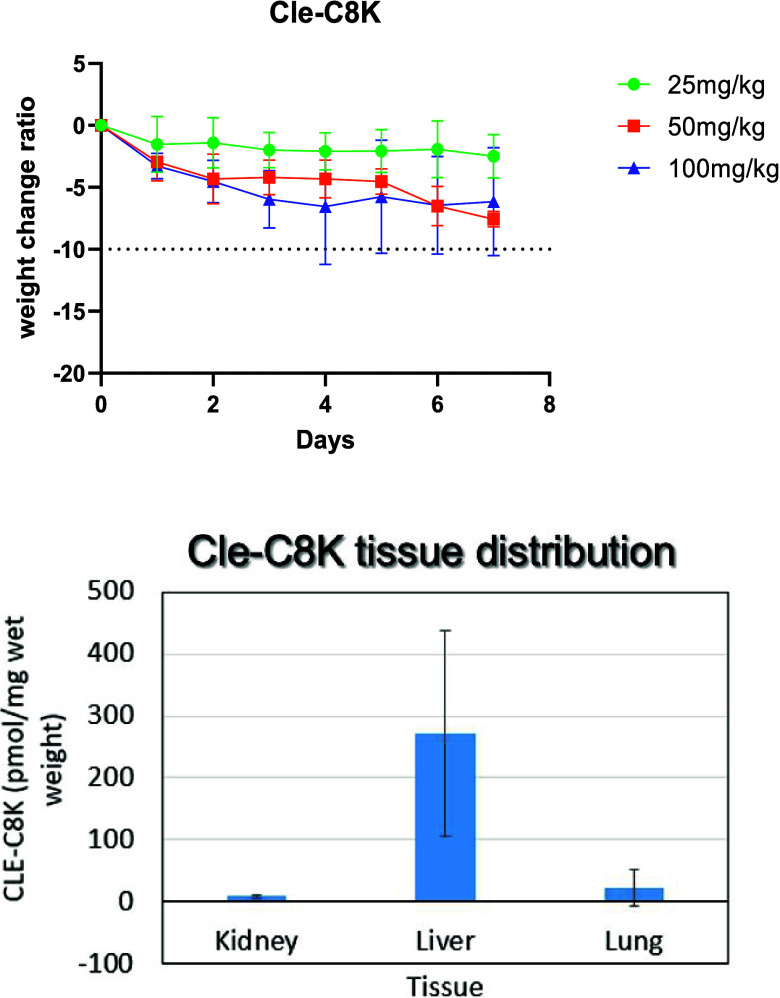
Analysis of the toxicity (MTD) and tissue distribution of **Cle-C8K** in C57BL/6 mice. (a) Effects of **Cle-C8K** on the body weights of the test animals at the stated doses after IP injection. (b) Analysis of tissue distribution of **Cle-C8K** at 50 mg/kg dosage in the kidney, liver, or lung of C57BL/6 mice (*n* = 4). Note that the plasma level of **Cle-C8K** is below the detection limit of our instrument.

## 3 Conclusions

We showed herein that Cle, a first generation HRH1 antagonist that selectively accumulates within the liver, could be used as a template to design small molecule epigenetic modifiers targeting HDACs and KDMs. The resulting HDACi and KDMi have midnanomolar to single digit micromolar IC_50_s and potency enhancement of 15–105 folds relative to Cle. Moreover, a cohort of these compounds elicited cancer cell line-dependent cytotoxicity. Representative lead KDMi, **Cle-C6K** and **Cle-C8K** caused transcriptome level perturbations that favored cell cycle progression inhibition and increased apoptosis. Moreover, we found that **Cle-C8K** is nontoxic and selectively accumulated in the liver of C57BL/6 mice. The potential antiliver cancer activity of **Cle-C8K** warrants further evaluation in *in vivo* models.

## 4 Materials and General Methods

Anhydrous solvents and reagents, high performance liquid chromatography (HPLC) grade or American Chemical Society (ACS) grade solvents were purchased from Sigma-Aldrich (St. Louis, MO, USA), Acros, VWR International (Radnor, PA, USA), or Thermo Fisher Scientific (Waltham, MA, USA) and were used without further purification. Analytical TLC was conducted using Analtech silica gel plates (60 F254), while purification was achieved using Analtech preparative TLC plates (UV 254, 2000 μm). Spot visualization was facilitated using UV light in conjunction with anisaldehyde/iodine stain. All reported yields are unoptimized. For column chromatography, 200–400 Mesh silica gel was utilized. HPLC analyses of products were carried out using Phenomenex Luna 91 5 μm C8(2) 100 Å LC column (4.6 × 250 mm) using Agilent 1260 Infinity II HPLC system. Water (solvent A) and MeCN (solvent B) containing 0.1% TFA were used as the mobile phase at a flow rate of 0.5 mL·min-1. The elution gradient profile was 95% A for the first 5 min, followed by linear gradient to 100% solvent B from 5 to 18 min, during which 100% B was reached; linear gradient to 5% solvent B from 22 to 28 min during which the mixture was returned to A, followed by 2 min (from 28 to 30 min) of 100%. The detection wavelength is at 254 nm and sample concentrations were 250 μM – 1 mM, injecting 30 μL. HPLC revealed that the target compounds have ≥ 95% purity. Nuclear magnetic resonance (NMR) spectra were acquired using Varian-Gemini 400 MHz, Bruker 500 MHz, or 700 MHz magnetic resonance spectrometers. ^1^H NMR spectra were referenced in parts per million (ppm) relative to the residual peaks of CHCl_3_ (7.24 ppm) in CDCl_3_, CHD_2_OD (4.78 ppm) in CD_3_OD, or DMSO-*d5* (2.49 ppm) in DMSO-*d6*. Similarly, ^13^C spectra were referenced relative to the central peak of the CDCl_3_ triplet (77.0 ppm), CD_3_OD septet (49.3 ppm), or DMSO-*d6* septet (39.7 ppm), employing complete heterodecoupling. Data from ‘fid’ files were processed using MestReNova LITE (version 5.2.5–5780) software. High-resolution mass spectra were recorded at the mass spectrometry facility of the Georgia Institute of Technology in Atlanta.

### Cell Culture

Cell lines Huh-7, Hep-G2, SK-HEP-1, DU145 were cultured in EMEM (Corning) with 10% FBS. MCF-7 was cultured in 10% FBS MEM without phenol red; MDA-MB-231, A549, and VERO cells were cultured in DMEM (Corning). LNCaP cells were cultured using RPMI-1640 supplemented with 10% FBS. mES CiA cells were cultured in DMEM (Corning) with 15% FBS supplemented with 100 units/mL penicillin/streptomycin, nonessential amino acids (NEAA; Gibco 11140–050), 10 mM HEPES buffer (Corning, 25–060-CI), 55 μM β-mercaptoethanol, leukemia inhibitory factor (LIF), 7.5 μg/mL blasticidin (InvivoGen, ant-bl-1), and 1.5 μg/mL puromycin (InvivoGen, ant-pr-1).

### MTS Assay

Cells were plated into a 96-well plate at a density of 5000 cells/100uL and allowed to adhere for 24 h prior to treatment. Subsequently, cells were exposed to varying concentrations of drugs for 72 h. All drug solutions were prepared in DMSO/DMEM, maintaining a DMSO concentration of 1%. The impact of these compounds on cell viability was assessed using the MTS assay, employing CellTiter 96 Aqueous One Solution and Cell-Titer 96 Non-Radioactive Cell Proliferation Assays from Promega (Madison, WI), following the manufacturer’s instructions. IC_50_ values were determined using Prism GraphPad 8.

### *In Vitro* HDAC Inhibition Assay

*In vitro* HDAC inhibition assay was performed through contractual agreement with BPS Bioscience.

### Chromatin *In Vivo* Assay (CiA)

For all experiments, mouse embryonic stem cells (mES) containing the CiA components were generated as previously described.^78–80^ Briefly, cells containing a replacement of a single Oct4 allele with a green fluorescent protein (GFP) gene were infected with lentivirus to stably integrate plasmids N118 (LV EF-1α-Gal-FKBPx1-HA-PGK-Blast) and N163 (nLV EF-1α-HP1α (CS)-Frbx2­(Frb+FrbWobb)-V5-PGK-Puro). mES cells where then were seeded into gelatin coated 96 well plates at 10,000 cells/well. Twenty-four h after seeding, the media was changed and replaced with media only (positive control), media with 6 nM rapamycin (negative control), or media with 6 nM rapamycin and a varying concentration of a compound of interest (0.1–7.5 μM for **Cle-C4K** through **Cle-C8K** and **VK-II-100**; 0.1–5 μM for **Cle-AC6K** through **Cle-AC8K**). Each condition was done in three biological triplicates. Rapamycin was obtained from LC Laboratories (Woburn, Massachusetts). After 48 h of exposure, cells were washed with PBS, collected using 0.25% trypsin-EDTA, quenched with growth media, and transferred to a nontissue culture treated U-bottom 96-well plate before being analyzed with an Attune NxT Acoustic Focusing Flow Cytometer with an autosampler (ThermoFisher). GFP signal was measured with a 488 nM laser with a 530/30 filter; autofluorescence was also measured with a 637 nM laser with a 670/14 filter. Collected mES cells were gated to include only live, single cell populations displaying no autofluorescence. A bifurcating gate was applied to generate a GFP positive (%GFP+) and GFP negative population for each sample, represented as a percentage of cells. Mean %GFP+ were calculated for the negative and positive control populations. Then, a percent inhibition value was calculated for each sample well and the negative control wells; % inhibition is defined as $\frac{1-\left(y-x\right)}{y-z}$ where x = the %GFP+ of the sample, y = the mean %GFP+ of the positive control wells, and z = the mean %GFP+ of the negative control wells.

### Western Blot

Cells were seeded into 6-well plates at a density of 1 × 10^6^ cells per well in culture media and allowed to incubate for 24 h. Various concentrations of SAHA and Cle compounds solutions in DMSO were added to the cell culture media to achieve a final DMSO concentration of 0.1%. Following treatment for 4 h, cells were washed with cold PBS and lysed using RIPA buffer (110 μL) supplemented with phosphatase inhibitor and protease inhibitor on ice for 15 min. The lysates were then centrifuged at 21000 xg for 15 min, and the supernatants were collected. Total protein concentration was determined using a BCA protein assay kit. Based on the protein concentration, the lysates were normalized to achieve equal protein concentrations, and 20–40 μg of each lysate was loaded onto TGX MIDI 4–15% gels and electrophoresed at 150 V for 64 min. Subsequently, the gel was transferred onto Turbo PDVF membranes, blocked with 5% BSA, and incubated overnight with specific antibodies at 4 °C. The following day, the membrane was washed with TBS-T, incubated with secondary antibody, and bands were quantified using the Odyssey CLx Image system.

### Cell Cycle Analysis

This analysis was carried adapting the manufacturer’s instructions (Canvax Biotech, S.L., Spain) with some modifications. Briefly, cells (1 × 10^6^ cells/well in 2 mL of medium) were seeded in six-well plates and allowed to attach for 24 h before treatment for another 24 h. Subsequently, cells were dissociated from the substrate using a cell scrapper, and the cell suspension was centrifuged at 500 xg for 5 min at 4 °C with the supernatant aspirated and discarded. Cells were then washed in 1 mL ice cold PBS and centrifuged again with the supernatant aspirated and discarded. Next, nucleic acid labeling was initiated by initially fixing the cells with 1 mL ice cold 70% ethanol added dropwise to the cell pellet while vortexing and the samples were stored on ice for at least 30 min. Thereafter, without disrupting the pellet, ethanol was carefully removed by centrifugation at 2500 xg for 5 min at 4 °C, and the cells were subsequently washed in 1 mL PBS. Again, PBS was removed by centrifugation at 2500 xg for 5 min and then the cells were resuspended in 200 μL of the staining solution (freshly prepared by mixing PBS with propidium iodide and RNase A in a ratio of 50:1:1, respectively) which should be protected from exposure to light. In preparation for flow cytometry analysis (using CytoFLEX S Flow Cytometer), the tubes containing the stained cells were incubated in the dark for 30 min at 37 °C and finally placed on ice before analysis.

### MTD and Tissue Distribution

All animal experiments were conducted with strict adherence to ethical guidelines and in accordance with the approved protocol by Georgia Institute of Technology Institutional Animal Care and Use Committee (IACUC Protocol ID: A100135). Mice (C57BL/6 strain) were injected with vector (dimethylacetamide (DMA)/Cremophor RH 40 (CRH)/Water (10% /20% /70%)) or vector solution of **Cle-C8K** at different concentrations using i.p. injection. Every dosage group has 4 mice. Each mouse was monitored and weighed daily for 7 days. Eight hours after the last dose, mice in the 50 mg/kg group were sacrificed, blood and key organs (kidney, liver, and lung) were collected; the organs were washed with PBS, and frozen in liquid nitrogen. The organs were processed and analyzed at the Georgia Institute of Technology mass spectrometry facility. Briefly, the frozen samples were thawed and weighed. Tissue samples were vortexed, homogenized with Tissuelyzer at least 2 × 5 min, centrifuged at 21,100x g for 5 min. The supernatant was transferred to new tube and diluted with isopropanol (IPA) at 10X vol (Note: plasma and liver samples were diluted with IPA at 3X and 100X vol, respectively). After vortex, 150 μL of diluted supernatant was transferred to LC vial. Sample (2 μL) was injected and HPLC was run BEH C18 column (150 × 2.1 mm, 1.7-μm particle size) using mobile phase A: H_2_O, 0.1% FA, mobile phase B: 20% ACN, 80% IPA, 0.1% Fa at column temperature of 60 °C. MS and targeted MS^2^ (MS/MS transition 594.35 → 393.24) were recorded in positive mode. The levels of **Cle-C8K** in each sample were quantified based on a preconstructed calibration curve (Supplementary Figure S14)

### Synthesis Procedure and Characterizations

#### Compound **2**: 2-(Pyrrolidin-1-ylmethyl)-1*H*-benzo­[*d*]­imidazole

A mixture of benzene-1,2-diamine (50 g, 0.59 mol) and ethyl 2-chloroacetate (72 g, 0.6 mol) in 5 M HCl (100 mL) was stirred at room temperature (rt) for 4 h during which the mixture turned yellow. Then, 1 M NH_4_OH solution in water was slowly added to the solution until white precipitates formed. The suspension was stirred for 1h at rt and filtered. The white precipitate was washed with water (2 × 50 mL) and cold ethanol (3 × 10 mL) to give the desired intermediate 2-(chloro-1-ylmethyl)-1*H*-benzo­[*d*]­imidazole **1**. The white powder was dried *in vacuo* overnight (48 g, 48%).

Compound **1** (10 g, 60 mmol) and pyrrolidine (7 g, 98.5 mmol) were dissolved in ethanol (50 mL), and the mixture was heated under reflux for 24 h. Solvent was evaporated off using a rotary evaporator, the resulting dark paste was dissolved in dichloromethane (DCM or CH_2_Cl_2_) and applied to a top of a silica gel chromatography column. The compound was flushed through with ethyl acetate:methanol:triethylamine (10:1:0.1) to give the desired compound **2** as a yellow powder (3.5 g, 36%).^1^H NMR (700 MHz, CDCl_3_) δ 7.53 (s, 1H), 7.20 (dd, *J* = 6.2, 3.3 Hz, 2H), 3.93 (s, 2H), 2.64 (s, 4H), 1.78 (s, 4H). ^13^C NMR (176 MHz, CDCl_3_) δ 152.9, 122.3, 54.4, 53.9, 23.7.

#### Compound **3**: 1-(4-Bromobenzyl)-2-(pyrrolidin-1-ylmethyl)-1*H*-benzo­[*d*]­imidazole

A solution of compound **2** (1 g, 1 equiv) in DMF (10 mL) was treated with NaH (0.24 g, 2 equiv) at 0 °C to rt for 30 min. 4-Bromobenzyl bromide (1.49 g, 1.2 equiv) was added and stirring continued at rt for about 20 h. After completion of the reaction as indicated by TLC, the reaction was quenched with water (50 mL) and extracted with CH_2_Cl_2_ (3 × 30 mL). The combined organic layer was dried over Na_2_SO_4_ and then filtered. The solvent was removed using a rotary evaporator, and the crude was purified by silica gel chromatography, eluting with DCM/Methanol = 15:1 to give compound **3** as a light-yellow powder (1.24 g, 67%). ^1^H NMR (700 MHz, CDCl_3_) δ 7.76 (d, *J* = 7.9 Hz, 1H), 7.41 (d, *J* = 8.9 Hz, 2H), 7.26 (d, *J* = 8.6 Hz, 1H), 7.22–7.17 (m, 2H), 6.97 (d, *J* = 8.5 Hz, 2H), 5.52 (s, 2H), 3.86 (s, 2H), 2.53 (s, 4H), 1.73 (s, 4H). ^13^C NMR (176 MHz, CDCl_3_) δ 152.5, 142.4, 135.8, 122.9, 122.2, 119.9, 109.6, 54.1, 53.2, 46.8, 23.6. HRMS (EI) *m*/*z* Calcd for C_19_H_20_BrN_3_ [M + H] ^+^: 369.0841, found 369.0849

#### Compound **5**: 1-(4-ethynylbenzyl)-2-(pyrrolidin-1-ylmethyl)-1*H*-benzo­[*d*]­imidazole

Compound **3** (0.5 g, 1 equiv), and TMS acetylene (0.186 mL, 2 equiv) were dissolved in dry acetonitrile (9 mL) under argon. Subsequently, Pd­(PPh_3_)_4_ (95 mg, 0.1 equiv), and CuI (31 mg, 0.06 equiv) were added, followed by Hunig’s base (1 mL). The reaction mixture was heated at 83 °C overnight. The reaction mixture was quenched with water (10 mL) and extracted with CH_2_Cl_2_ (3 × 20 mL) and washed with NH_4_OH/NH_4_Cl 1:1 (10 mL), the two layers were separated, and the combined organic layer was washed sequentially with conc. NH_4_OH/NH_4_Cl 1:1 (2 × 10 mL), brine (30 mL), dried over Na_2_SO_4_, and then filtered. The solvent was removed using a rotary evaporator, and the crude material was purified using preparative TLC, eluting with CH_2_Cl_2_: MeOH (10:1), v/v; to afford intermediate product as compound **4** (0.46 g, 88%).

The intermediate compound **4** (0.460 g) was dissolved in 10 mL THF at 0 °C. TBAF (0.62 g, 2 equiv) was added, and the reaction mixture was stirred at rt for 1 h. The completion of the reaction was indicated by TLC. The reaction mixture was quenched with water (20 mL) and extracted with CH_2_Cl_2_ (3 × 20 mL). The organic phases were combined, dried over Na_2_SO_4_, and then filtered. The solvent was removed using a rotary evaporator, and the crude product was purified using column chromatography: MeOH (10:2), v/v] to afford a compound **5** as a yellow solid (0.34 g, 90% yield). ^1^H NMR (700 MHz, CDCl_3_) δ 7.79 (d, *J* = 8.0 Hz, 1H), 7.43 (d, *J* = 6.5 Hz, 2H), 7.28–7.25 (m, 1H), 7.23 (d, *J* = 14.1 Hz, 2H), 7.06 (d, *J* = 8.5 Hz, 2H), 5.58 (s, 2H), 3.87 (s, 2H), 3.08 (s, 1H), 2.56 (s, 4H), 1.73 (s, 4H). ^13^C NMR (176 MHz, CDCl_3_) δ 152.5, 142.4, 135.9, 126.5, 122.8, 122.1, 121.5, 109.6, 83.1, 77.6, 54.2, 53.2, 47.1, 23.7. HRMS (EI) *m*/*z* Calcd for C_21_H_21_N_3_ [M + H] ^+^: 315.1735, found 315.1732.

#### Compound Cle-C6: 6-(4-(4-((2-(Pyrrolidin-1-ylmethyl)-1*H*-benzo­[*d*]­imidazol-1-yl)­methyl)­phenyl)-1*H*-1,2,3-triazol-1-yl)-*N*-(trityloxy)­hexanamic Acid

To a solution of compound **5** (25 mg, 0.08 mmol) in THF (2 mL) was added 6-azido-N-(trityloxy)­hexanamide (50 mg, 0.12 mmol). Subsequently, CuI (1.5 mg, 0.1 equiv) was added, and the mixture was purged with argon gas for 5 min while stirring. Hunig’s base (0.2 mL) was added and the mixture first turned to green then yellow after stirring at rt overnight. The reaction was quenched with sat. NH_4_OH:NH_4_Cl (1:4) (15 mL) and extracted with DCM (2 × 10 mL). The DCM layer was dried with Na_2_SO_4_, solvent was evaporated off and the crude was purified using preparative TLC, eluting with ethyl acetate: hexane (7:3). The intermediate was deprotected by adding the 10% TFA in THF (2 mL) solution and stir reaction mixture 30 min at rt and the residue dried in vacuo to furnish Cle-C6 as a yellow foam (12 mg, 31%). ^1^H NMR (500 MHz, CD_3_OD) δ 8.32 (s, 1H), 7.80 (d, *J* = 8.1 Hz, 2H), 7.75 (s, 1H), 7.50 (s, 1H), 7.32 (s, 2H), 7.21 (d, *J* = 7.9 Hz, 2H), 5.59 (s, 2H), 4.79 (s, 2H), 4.42 (s, 2H), 3.59 (s, 4H), 2.13 (s, 5H), 1.95 (s, 2H), 1.65 (s, 2H), 1.35 (s, 2H). ^13^C NMR (176 MHz, CD_3_OD) δ 148.0, 147.0, 143.3, 137.1, 136.8, 134.1, 131.7, 128.3, 127.3, 125.1, 124.1, 122.4, 120.5, 111.8, 56.5, 51.6, 51.2, 47.7, 30.8, 26.8, 25.9, 24.1. HPLC retention time = 15.566 min. HRMS (ESI) *m*/*z* Calcd for C_27_H_34_O_2_N_7_ [M + H] ^+^: 488.2768, found 488.2754.

#### Compound Cle-C7: 7-(4-(4-((2-(Pyrrolidin-1-ylmethyl)-1*H*-benzo­[*d*]­imidazol-1-yl)­methyl)­phenyl)-1*H*-1,2,3-triazol-1-yl)-*N*-(trityloxy)­heptanamic Acid

The reaction of compound **5** (25 mg, 0.08 mmol), 7-azido-N-(trityloxy)­heptanamide (50 mg, 0.12 mmol), CuI (1.5 mg, 0.1 equiv) and Hunig’s base (0.2 mL) in THF (2 mL) as described for **Cle-C6** furnished **Cle-C7** as a yellow foam (15 mg, 38%).^1^H NMR (700 MHz, CDCl_3_) δ 7.73 (d, *J* = 7.5 Hz, 2H), 7.69 (d, *J* = 8.3 Hz, 2H), 7.23–7.19 (m, 2H), 7.17 (t, *J* = 8.1 Hz, 1H), 7.09 (d, *J* = 8.3 Hz, 2H), 5.53 (s, 2H), 4.23 (t, *J* = 7.1 Hz, 2H), 3.90 (s, 2H), 2.58 (s, 4H), 2.06 (s, 2H), 1.76 (t, *J* = 7.2 Hz, 2H), 1.72 (d, *J* = 3.5 Hz, 4H), 1.56–1.47 (m, 2H), 1.26–1.13 (m, 4H). ^13^C NMR (176 MHz, CDCl_3_) δ 170.9, 152, 147.1, 142, 136.4, 135.7, 130.2, 127.2, 126.1, 123.1, 122.4, 120, 119.6, 110, 54.2, 52.7, 50.1, 47.2, 32.6, 29.9, 28.0, 25.7, 25.1, 23.7. HPLC retention time = 15.743 min. HRMS (ESI) *m*/*z* Calcd for C_28_H_36_O_2_N_7_ [M + H] ^+^: 502.2925, found 502.2910.

#### Compound Cle-C8: 8-(4-(4-((2-(Pyrrolidin-1-ylmethyl)-1*H*-benzo­[*d*]­imidazol-1-yl)­methyl)­phenyl)-1*H*-1,2,3-triazol-1-yl)-*N*-(trityloxy)­octanamide

The reaction of compound **5** (25 mg, 0.08 mmol), 8-azido-N-(trityloxy)­octanamide (50 mg, 0.11 mmol), CuI (1.5 mg, 0.1 equiv) and Hunig’s base (0.2 mL) in THF (2 mL) as described for **Cle-C6** furnished **Cle-C8** as a yellow foam (14 mg, 27%). ^1^H NMR (500 MHz, CD_3_OD) δ 8.31 (s, 1H), 7.79 (d, *J* = 8.1 Hz, 2H), 7.75 (s, 1H), 7.51 (s, 1H), 7.32 (s, 2H), 7.21 (d, *J* = 7.9 Hz, 2H), 5.60 (s, 2H), 4.81 (s, 2H), 4.40 (s, 2H), 3.59 (s, 4H), 2.14 (s, 4H), 2.07 (s, 2H), 1.88 (s, 2H), 1.55 (s, 2H), 1.30 (s, 6H). ^13^C NMR (176 MHz, CD_3_OD) δ 161.4, 148, 146.9, 143.1, 137, 136.7, 131.7, 128.3, 127.3, 125.1, 124.2, 122.4, 120.4, 111.9, 56.5, 51.4, 47.7, 31.1, 29.8, 29.5, 27.2, 26.5, 24.1. HPLC retention time = 17.154 min. HRMS (ESI) *m*/*z* Calcd for C_29_H_38_O_2_N_7_ [M + H] ^+^: 516.3081, found 516.3066.

#### Compound Cle-PH: ((*E*)-*N*-Hydroxy-3-(4-((2-(pyrrolidin-1-ylmethyl)-1*H*-benzo­[*d*]­imidazol-1-yl)­methyl)­phenyl)­acrylamide)

Compound **3** (50 mg, 0.14 mmol) and N-(trityloxy) acrylamide (53 mg, 0.16 mmol) were dissolved in dry DMF (1 mL) under an argon atmosphere. Subsequently, tetrakis (triphenylphosphine) palladium (0) (5 mg, 5% equiv) and cesium carbonate (88 mg, 2 equiv) were added. The mixture was then heated to 120 °C for 15 h. After the reaction was completed, reaction mixture diluted with DCM (30 mL) and filtered through Celite, and solvent was removed under reduced pressure. Once the solvent was fully evaporated, 50 mL of water was added, and the product was extracted with DCM (3 × 30 mL). The organic layer was washed with brine, dried over anhydrous Na_2_SO_4_, and concentrated under reduced pressure to yield the crude product. The crude product was purified using preparatory TLC, eluting with ethyl acetate and hexane (1:1) to give the protected hydroxamate intermediate as a yellow solid. This intermediate was then deprotected using 10% trifluoroacetic acid (TFA) in THF (0.5 mL). The crude material obtained was further purified by silica gel column chromatography, using a mixture of methanol and dichloromethane (1:12, v/v) as the eluent to give **Cle-PH** as a yellow solid (12 mg, 86%). yield after two steps). ^1^H NMR (700 MHz, CD_3_OD) δ 7.67 (d, *J* = 13.3 Hz, 1H), 7.52 (d, *J* = 8.2 Hz, 2H), 7.50 (s, 1H), 7.39 (d, *J* = 7.9 Hz, 1H), 7.27–7.24 (m, 2H), 7.15 (d, *J* = 8.3 Hz, 2H), 6.47 (d, *J* = 16.0 Hz, 1H), 5.64 (s, 2H), 4.02 (s, 2H), 2.71 (s, 4H), 1.30 (s, 4H).^13^C NMR (176 MHz, CD_3_OD) δ 147.0, 143.3, 140.6, 138.8, 136.8, 136.2, 130.4, 129.2, 128.1, 125.1, 124.1, 120.5, 119.1, 111.8, 56.5, 51.6, 47.6, 24.1. HPLC retention time = 16.653 min. HRMS (ESI) *m*/*z* Calcd for C_22_H_25_O_2_N_4_ [M + H] ^+^: 377.1972, found 377.1972.

#### Compound Cle-PPH: (*E*)-*N*-Hydroxy-3-(4′-((2-(pyrrolidin-1-ylmethyl)-1*H*-benzo­[*d*]­imidazol-1-yl)­methyl)-[1,1′-biphenyl]-4-yl)­acrylamide

##### 4.8.8.1 Step-1: Suzuki Coupling

Compound **3** (50 mg, 0.14 mmol, 1 equiv) and (4-hydroxyphenyl) boronic acid (22 mg, 0.16 mmol, 1.2 equiv) were dissolved in 1,4-dioxane (1.8 mL). Cesium carbonate (80 mg, dissolved in 200 μL of water) was gently added to the mixture. The system was then flushed with argon for 5 to 10 min. The reaction mixture was heated to 120 °C for 15 h. After completion, the reaction was filtered through Celite, and the solvent was removed under reduced pressure. After complete evaporation of the solvent, water (50 mL) was added, and the crude was extracted with DCM (2 × 30 mL). The organic layer was washed with brine, dried over Na_2_SO_4_ (anh.), and concentrated under reduced pressure to give a residue which was purified using column chromatography, eluting with a mixture of EtOAc and hexane (1:1) to afford intermediate compound **10** as a yellow solid (36.5 mg, 74% yield).

##### 4.8.8.2 Step-II: Triflate Formation

The intermediate compound **10** was dissolved in anhydrous DCM (5 mL) under an argon atmosphere. Pyridine (11.5 μL, 0.18 mmol, 1.5 equiv) was added, and the mixture was stirred in an ice bath for 10 min. After this, triflic anhydride (24 μL, 1.5 equiv) was added, and the ice bath was removed, allowing the reaction to come to rt. The reaction was considered complete after 1 h, as confirmed by TLC analysis. Subsequently, the reaction was quenched with a saturated solution of NaHCO_3_ (10 mL). The mixture was then extracted with DCM (3 × 30 mL), and the organic layer was washed with brine, dried over Na_2_SO_4_ (anh.), and concentrated under reduced pressure to give the crude triflate intermediate **11** (38 mg, 77% yield).

##### 4.8.8.3 Step-III: Heck Coupling and Trityl Group Deprotection

The triflate intermediate **11** and N-(trityloxy) acrylamide **8** (29 mg, 1.2 equiv) were dissolved in dry DMF (1 mL) under an argon atmosphere. Subsequently, tetrakis (triphenylphosphine) palladium (0) (5 mg, 5% equiv) and cesium carbonate (57 mg, 0.17 mmol, 2 equiv) were added. The mixture was then heated to 120 °C for 15 h. After the reaction was completed, reaction mixture diluted with DCM (30 mL) and filtered through Celite, and the solvent was removed under reduced pressure. Once the solvent was fully evaporated, 50 mL of water was added, and the crude was extracted with DCM (3 × 30 mL). The organic layer was washed with brine, dried over anhydrous Na_2_SO_4_ and concentrated under reduced pressure to yield the crude product. The crude product was purified using preparatory TLC, eluting with ethyl acetate and hexane (1:1) to furnish the protected hydroxamate intermediate **12** as a yellow solid. This intermediate was then deprotected using 10% trifluoroacetic acid (TFA) in THF (0.5 mL). The crude material obtained was further purified by silica gel column chromatography, eluting with methanol and DCM (1:12, v/v) to afford **Cle-PPH** as a yellow solid (28 mg, 84% after two steps). ^1^H NMR (500 MHz, CD_3_OD) δ 7.75 (d, *J* = 4.0 Hz, 2H), 7.68–7.45 (m, 7H), 7.32 (d, *J* = 7.6 Hz, 2H), 7.20 (d, *J* = 14.5 Hz, 2H), 6.48 (d, J = 20.0 Hz, 1H), 5.61 (s, 2H), 4.80 (s, 2H), 3.59 (s, 4H), 2.14 (s, 4H).^13^C NMR (176 MHz, CD_3_OD) δ 161.7, 147.0, 142.7, 141.3, 136.8, 136.7, 135.4, 129.4, 128.6, 128.3, 128.2, 125.1, 124.1, 120.6, 111.9, 56.5, 51.5, 47.7, 24.1. HPLC retention time = 16.908 min. HRMS (ESI) *m*/*z* Calcd for C_28_H_29_O_2_N_4_ [M + H] ^+^: 453.2285, found 453.2274.

#### Compound Cle-C4K: 1-(4-(4-(4-((1*H*-Benzo­[*d*]­imidazol-1-yl)­methyl)­phenyl)-1*H*-1,2,3-triazol-1-yl)­butyl)-3-hydroxy-2-methylpyridin-4­(1*H*)-one

Compound **5** (25 mg, 0.079 mmol, 1 equiv) and 1-(4-azidobutyl)-3-(benzyloxy)-2-methylpyridin-4­(1H)-one (50 mg, 0.16 mmol, 2 equiv) were dissolved in THF (2 mL). Then CuI (1.5 mg, 0.1 equiv) was added with stirring and the mixture was purged with argon gas for 5 min, followed by addition of Hunig’s base (0.2 mL). The solution turned green then yellow overnight. The reaction was quenched with sat. NH_4_OH:NH_4_Cl = 1:4 solution (15 mL) and extracted with DCM (2 × 10 mL). Then the organic layer was dried over Na_2_SO_4_, solvent was evaporated, and the crude was purified on preparative TLC eluting with ethyl acetate: hexane = 1:1. The intermediate was deprotected by adding the concentrated HCl (1 mL) into its THF (1 mL) solution and stirred overnight. Solvent was evaporated *in vacuo* and the desired product was obtained as yellow foam (29.5 mg, 68%). ^1^H NMR (700 MHz, CD_3_OD) δ 8.27 (bs, 1H), 7.74 (d, *J* = 8.0 Hz, 2H), 7.65 (d, *J* = 8.0 Hz, 1H), 7.53 (s, 1H), 7.36 (d, *J* = 7.9 Hz, 1H), 7.25–7.14 (m, 4H), 6.34 (d, 1H), 5.62 (s, 2H), 4.45 (s, 2H), 4.02 (s, 2H), 3.89 (s, 2H), 2.53 (s, 4H), 2.35 (s, 3H), 1.96 (s, 2H), 1.69 (m, 6H). ^13^C NMR (176 MHz, CD_3_OD) δ 173.1, 156.4, 150.9, 149.8, 145.2, 141.3, 140.8, 139.4, 135.1, 133.4, 131.0, 129.5, 126.1, 122.2, 115.2, 114.1. 57.6, 56.9, 55.8, 53.2, 50.6, 31.1, 30.5, 27.0, 14.4. HPLC retention time = 15.686 min. HRMS (ESI) *m*/*z* Calcd for C_31_H_35_O_2_N_7_ [M + H] ^+^: 538.2925, found 538.2924.

#### Compound Cle-C5K: 1-(5-(4-(4-((1*H*-Benzo­[*d*]­imidazol-1-yl)­methyl)­phenyl)-1*H*-1,2,3-triazol-1-yl)­pentyl)-3-hydroxy-2-methylpyridin-4­(1*H*)-one

Compound **5** (25 mg, 0.08 mmol, 1 equiv) and 1-(5-azidopentyl)-3-(benzyloxy)-2-methylpyridin-4­(1H)-one (50 mg, 0.16 mmol, 2 equiv) were dissolved in THF (2 mL). Then CuI (1.5 mg, 0.1 equiv) was added with stirring and the mixture was purged with argon gas for 5 min, followed by addition of Hunig’s base (0.2 mL). The solution turned green then yellow overnight. The reaction was quenched with sat. NH_4_OH:NH_4_Cl = 1:4 solution (15 mL) and extracted with DCM (2 × 10 mL). Then the organic layer was dried over Na_2_SO_4_, solvent was evaporated, and the crude was purified on preparative TLC eluting with ethyl acetate: hexane = 1:1. The intermediate was deprotected by adding the concentrated HCl (1 mL) into its THF (1 mL) solution and stirred overnight. Solvent was evaporated *in vacuo* and the desired product was obtained as yellow foam (23 mg, 52%). ^1^H NMR (700 MHz, CD_3_OD) δ 8.28 (s, 1H), 7.75 (d, *J* = 8.2 Hz, 2H), 7.66 (d, *J* = 7.6 Hz, 1H), 7.54 (d, *J* = 7.3 Hz, 1H), 7.39 (s, 1H), 7.22 (dd, *J* = 15.0, 7.6 Hz, 4H), 6.34 (d, *J* = 6.9 Hz, 1H), 5.64 (s, 2H), 4.43 (s, 2H), 3.98 (s, 2H), 3.93 (s, 2H), 2.59 (s, 4H), 2.38 (s, 3H), 1.97 (m, 2H), 1.73 (m, 6H), 1.36 (m, *J* = 8.9, 8.3 Hz, 2H). ^13^C NMR (176 MHz, CD_3_OD) δ 170.5, 148.3, 147.3, 138.7, 138.2, 132.6, 131.2, 128.4, 126.9, 124.3, 123.5, 122.3, 112.5, 71.5, 61.5, 55, 54.8, 51.1, 48.1, 40.4, 31, 30.6, 24.5, 24.2, 20.8, 14.4, 11.8. HPLC retention time = 15.312 min. HRMS (ESI) *m*/*z* Calcd for C_32_H_37_O_2_N_7_ [M + H] ^+^: 552.30814, found 552.30838.

#### Compound Cle-C6K: 1-(6-(4-(4-((1*H*-Benzo­[*d*]­imidazol-1-yl)­methyl)­phenyl)-1*H*-1,2,3-triazol-1-yl)­hexyl)-3-hydroxy-2-methylpyridin-4­(1*H*)-one

Compound **5** (25 mg, 0.08 mmol, 1 equiv) and 11-(6-azidohexyl)-3-(benzyloxy)-2-methylpyridin-4­(1H)-one (50 mg, 0.16 mmol, 2 equiv) were dissolved in THF (2 mL). Then CuI (1.5 mg, 0.1 equiv) was added with stirring and the mixture was purged with argon gas for 5 min, followed by addition of Hunig’s base (0.2 mL). The solution turned green then yellow overnight. The reaction was quenched with sat. NH_4_OH:NH_4_Cl = 1:4 solution (15 mL) and extracted with DCM (2 × 10 mL). Then the organic layer was dried over Na_2_SO_4_, solvent was evaporated, and the crude was purified on preparative TLC eluting with ethyl acetate: hexane = 3:7. The intermediate was deprotected by adding the concentrated HCl (1 mL) into its THF (1 mL) solution and stirred overnight. Solvent was evaporated *in vacuo* and the desired product was obtained as yellow foam (18.3 mg, 41%). ^1^H NMR (700 MHz, CD_3_OD) δ 8.29 (s, 1H), 7.76 (s, 2H), 7.66 (d, *J* = 8.0 Hz, 1H), 7.57 (s, 1H), 7.40 (s, 1H), 7.22 (s, 4H), 6.36 (d, *J* = 6.9 Hz, 1H), 5.65 (s, 2H), 4.42 (s, 2H), 3.98 (s, 4H), 2.64 (s, 4H), 2.39 (s, 3H), 1.95 (s, 2H), 1.73 (s, 4H), 1.38 (s, 4H). ^13^C NMR (176 MHz, CD_3_OD) δ 170.4, 148.3, 147.2, 138.7, 138.2, 132.6, 131.2, 128.4, 126.9, 124.3, 123.6, 122.3, 119.7, 112.6, 111.6, 61.5, 54.8, 51.2, 48.1, 40.4, 31.5, 31, 27, 26.6, 24.4, 20.8, 14.4, 11.8. HPLC retention time = 15.707 min. HRMS (ESI) *m*/*z* Calcd for C_33_H_39_O_2_N_7_ [M + H] ^+^: 566.32380, found 566.32374.

#### Compound Cle-C7K: 1-(7-(4-(4-((1*H*-Benzo­[*d*]­imidazol-1-yl)­methyl)­phenyl)-1*H*-1,2,3-triazol-1-yl)­heptyl)-3-hydroxy-2-methylpyridin-4­(1*H*)-one

Compound **5** (25 mg, 0.08 mmol, 1 equiv) and 1-(7-azidoheptyl)-3-(benzyloxy)-2-methylpyridin-4­(1H)-one (50 mg, 0.16 mmol, 2 equiv) were dissolved in THF (2 mL). Then CuI (1.5 mg, 0.1 equiv) was added with stirring and the mixture was purged with argon gas for 5 min, followed by addition of Hunig’s base (0.2 mL). The solution turned green then yellow overnight. The reaction was quenched with sat. NH_4_OH:NH_4_Cl = 1:4 solution (15 mL) and extracted with DCM (2 × 10 mL). Then the organic layer was dried over Na_2_SO_4_, solvent was evaporated, and the crude was purified on preparative TLC eluting with ethyl acetate: hexane = 3:7. The intermediate was deprotected by adding the concentrated HCl (1 mL) into its THF (1 mL) solution and stirred overnight. Solvent was evaporated *in vacuo* and the desired product was obtained as yellow foam (22.3 mg, 48%). ^1^H NMR (700 MHz, CDCl_3_) δ 7.77 (d, *J* = 8.0 Hz, 3H), 7.30–7.08 (m, 5H), 6.34 (s, 1H), 5.60 (s, 2H), 4.37 (s, 1H), 3.88 (s, 2H), 3.85–3.69 (m, 2H), 2.57 (s, 4H), 2.34 (s, 3H), 1.92 (s, 2H), 1.73 (s, 4H), 1.67 (s, 2H), 1.33 (s, 6H). ^13^C NMR (176 MHz, CDCl_3_) δ 169.4, 152.5, 147.2, 146.3, 142.3, 136.6, 130, 127.8, 127.1, 126, 122.7, 122, 119.6, 111, 109.7, 54.2, 53.8, 53.2, 50.2, 47.1, 30.7, 30.1, 29.7, 28.4, 26.1, 23.6, 11.8. HPLC retention time = 15.954 min. HRMS (ESI) *m*/*z* Calcd for C_34_H_41_O_2_N_7_ [M + H] ^+^: 580.33945, found 580.33966.

#### Compound Cle-C8K: 1-(8-(4-(4-((1*H*-Benzo­[*d*]­imidazol-1-yl)­methyl)­phenyl)-1*H*-1,2,3-triazol-1-yl)­octyl)-3-hydroxy-2-methylpyridin-4­(1*H*)-one

Compound **5** (25 mg, 0.08 mmol, 1 equiv) and 1-(8-azidooctyl)-3-(benzyloxy)-2-methylpyridin-4­(1H)-one (50 mg, 0.16 mmol, 2 equiv) were dissolved in THF (2 mL). Then CuI (1.5 mg, 0.1 equiv) was added with stirring and the mixture was purged with argon gas for 5 min, followed by addition of Hunig’s base (0.2 mL). The solution turned green then yellow overnight. The reaction was quenched with sat. NH_4_OH:NH_4_Cl = 1:4 solution (15 mL) and extracted with DCM (2 × 10 mL). Then the organic layer was dried over Na_2_SO_4_, solvent was evaporated, and the crude was purified on preparative TLC eluting with ethyl acetate: hexane = 3:7. The intermediate was deprotected by adding the concentrated HCl (1 mL) into its THF (1 mL) solution and stirred overnight. Solvent was evaporated *in vacuo* and the desired product was obtained as yellow foam (19.5 mg, 41%). ^1^H NMR (700 MHz, CD_3_OD) δ 7.90 (s, 1H), 7.75 (s, 2H), 7.65 (s, 1H), 7.55 (s, 1H), 7.38 (s, 1H), 7.21 (d, *J* = 15.5 Hz, 4H), 6.36 (s, 1H), 5.64 (s, 2H), 4.40 (s, 2H), 3.94 (m, 4H), 2.58 (s, 4H), 2.38 (s, 3H), 1.93 (s, 2H), 1.71 (m, 6H), 1.30 (s, 8H). ^13^C NMR (176 MHz, CD_3_OD) δ 170.4, 153.7, 148.2, 147.3, 142.6, 138.7, 138.2, 136.8, 132.6, 131.2, 128.4, 126.9, 124.3, 123.5, 122.3, 119.7, 112.5, 111.6, 55.1, 53.2, 51.4, 48.1, 31.6, 31, 27.1, 24.4, 11.8. HPLC retention time = 16.230 min. HRMS (ESI) *m*/*z* Calcd for C_35_H_43_O_2_N_7_ [M + H] ^+^: 594.35510, found 594.35562.

#### Cle-AC6K: 3-Hydroxy-2-methyl-1-(8-(4-((2-(pyrrolidin-1-ylmethyl)-1*H*-benzo­[d]­imidazol-1-yl)­methyl)­phenyl)­oct-7-yn-1-yl)­pyridin-4­(1H)-one

Compound **3** (104 mg, 1 equiv) and 3-((4-methoxybenzyl)­oxy)-2-methyl-1-(oct-7-yn-1-yl)­pyridin-4­(1H)-one **(15 a)** (100 mg, 1 equiv) was dissolved in dry acetonitrile (5 mL) under argon. Subsequently, Pd­(PPh_3_)_4_ (10 mg 0.05 equiv) and CuI (3 mg, 0.06 equiv) were added, followed by Hunig’s base (0.5 mL). The reaction mixture was heated at 75 °C overnight. The reaction mixture was quenched with water (10 mL) and extracted with CH_2_Cl_2_ (3 × 20 mL) and conc. NH_4_OH/NH_4_Cl 1:1 (10 mL), the two layers separated, and the organic layer was washed sequentially with conc. NH_4_OH/NH_4_Cl 1:1 (2 × 10 mL), brine (30 mL) and dried over Na_2_SO_4,_ and then filtered. The solvent was removed using a rotary evaporator, and the crude material was purified using preparative TLC, eluting with CH_2_Cl_2_: MeOH (10:1), v/v; to afford intermediate product. The intermediate was deprotected by stirring in 10% TFA in CH_2_Cl_2_ (2 mL) at rt for 1h. The crude material was purified on silica gel column chromatography, eluting with MeOH: CH_2_Cl_2_ (2:10), v/v, to afford **Cle-AC6K** as light brown solid (43 mg, 30% after two steps). ^1^H NMR (700 MHz, CD_3_OD) δ 7.64 (d, *J* = 7.3 Hz, 1H), 7.56 (d, *J* = 7.0 Hz, 1H), 7.34 (d, *J* = 7.1 Hz, 1H), 7.25 (dd, *J* = 16.8, 7.0 Hz, 5H), 7.04 (d, *J* = 7.8 Hz, 2H), 6.36 (d, *J* = 6.9 Hz, 1H), 5.59 (s, 2H), 4.01 (s, 2H), 3.89 (s, 1H), 2.55 (s, 4H), 2.37 (m, 5H), 1.75 (m, 6H), 1.56 (s, 2H), 1.45 (s, 2H), 1.37 (s, 2H). ^13^C NMR (176 MHz, CD_3_OD) δ 170.4, 153.7, 142.6, 136.8, 127.7, 124.8, 123.5, 119.7, 112.5, 111.5, 91, 81.2, 55.2, 55, 53.1, 51, 48, 31.7, 29.5, 27.1, 24.1, 19.8, 11.8. HPLC retention time = 16.629 min. HRMS (ESI) *m*/*z* Calcd for C_33_H_39_N_4_O_2_ [M + H] ^+^: 523.3075, found 523.3098.

#### Cle-AC7K: 3-Hydroxy-2-methyl-1-(9-(4-((2-(pyrrolidin-1-ylmethyl)-1*H*-benzo­[d]­imidazol-1-yl)­methyl)­phenyl)­non-8-yn-1-yl)­pyridin-4­(1H)-one

**Compound 3** (104 mg, 1 equiv) and 3-((4-methoxybenzyl)­oxy)-2-methyl-1-(non-8-yn-1-yl)­pyridin-4­(1H)-one **(15 b)** (100 mg, 1 equiv) was dissolved in dry acetonitrile (5 mL) under argon. Subsequently, Pd­(PPh_3_)_4_ (9 mg 0.05 equiv) and CuI (3 mg, 0.06 equiv) were added, followed by Hunig’s base (0.5 mL). The reaction mixture was heated at 75 °C overnight. The reaction mixture was quenched with water (10 mL) and extracted with CH_2_Cl_2_ (3 × 20 mL) and conc. NH_4_OH/NH_4_Cl 1:1 (10 mL), the two layers separated, and the organic layer was washed sequentially with conc. NH_4_OH/NH_4_Cl 1:1 (2 × 10 mL), brine (30 mL) and dried over Na_2_SO_4,_ and then filtered. The solvent was removed using a rotary evaporator, and the crude material was purified using preparative TLC, eluting with CH_2_Cl_2_: MeOH (10:1), v/v; to afford intermediate product. The intermediate was deprotected by stirring in 10% TFA in CH_2_Cl_2_ (2 mL) solution for 1h at rt. The crude material was purified on silica gel chromatography, eluting with MeOH: CH_2_Cl_2_ (2:10), v/v, to afford **Cle-AC7K** as light brown solid (38 mg, 26% after two steps). ^1^H NMR (700 MHz, CDCl_3_) δ 7.76 (s, 1H), 7.30 (d, *J* = 8.0 Hz, 2H), 7.23 (d, *J* = 35.1 Hz, 5H), 7.01 (s, 2H), 6.36 (s, 1H), 5.55 (s, 2H), 3.85 (s, 4H), 2.53 (s, 4H), 2.38 (d, *J* = 7.4 Hz, 5H), 1.73 (s, 7H), 1.59 (s, 2H), 1.49 (s, 3H), 1.39 (s, 2H). ^13^C NMR (176 MHz, CDCl_3_) δ 169.5, 152.5, 146.3, 142.3, 136.8, 136.3, 135.9, 131.9, 127.7, 126.5, 126, 123, 122.8, 122.1, 119.9, 111.0, 109.7, 90.2, 54.2, 53.9, 53.2, 47.1, 30.9, 29.7, 28.4, 26, 23.6, 19.3, 11. 9. HPLC retention time = 16.793 min. HRMS (ESI) *m*/*z* Calcd for C_34_H_41_N_4_O_2_ [M + H] ^+^: 537.3231, found 537.3281.

#### Cle-AC8K: 3-Hydroxy-2-methyl-1-(10-(4-((2-(pyrrolidin-1-ylmethyl)-1*H*-benzo­[d]­imidazol-1-yl)­methyl)­phenyl)­dec-9-yn-1-yl)­pyridin-4­(1*H*)-one

**Compound 3** (116 mg, 1 equiv) and 1-(dec-9-yn-1-yl)-3-((4-methoxybenzyl)­oxy)-2-methylpyridin-4­(1H)-one **(15c)** (100 mg 1 equiv) was dissolved in dry acetonitrile (5 mL) under argon. Subsequently, Pd­(PPh_3_)_4_ (10 mg 0.05 equiv) and CuI (3 mg, 0.06 equiv) were added, followed by Hunig’s base (0.5 mL). The reaction mixture was heated at 75 °C overnight. The reaction mixture was quenched with water (10 mL) and extracted with CH_2_Cl_2_ (3 × 20 mL) and conc. NH_4_OH/NH_4_Cl 1:1 (10 mL), the two layers separated, and the organic layer was washed sequentially with conc. NH_4_OH/NH_4_Cl 1:1 (2 × 10 mL), brine (30 mL) and dried over Na_2_SO_4,_ and then filtered. The solvent was removed using a rotary evaporator, and the crude material was purified using preparative TLC, eluting with CH_2_Cl_2_: MeOH (10:1), v/v; to afford intermediate product. The intermediate was deprotected by stirring in 10% TFA in CH_2_Cl_2_ (2 mL) for 1h at rt. The crude material was purified on silica gel column chromatography, eluting with MeOH: CH_2_Cl_2_ (2:10), v/v, to afford **Cle-AC8K** as light brown solid (46 mg, 31% after two steps). ^1^H NMR (700 MHz, CDCl_3_) δ 7.76 (d, *J* = 7.9 Hz, 1H), 7.30 (d, *J* = 8.2 Hz, 2H), 7.21 (s, 3H), 7.01 (d, *J* = 8.1 Hz, 2H), 6.37 (d, *J* = 6.8 Hz, 1H), 5.55 (s, 2H), 3.85 (s, 2H), 3.82 (t, *J* = 6.1 Hz, 2H), 2.54 (s, 4H), 2.37 (d, *J* = 6.0 Hz, 5H), 1.73 (s, 6H), 1.34 (s, 6H). ^13^C NMR (176 MHz, CDCl_3_) δ 169.5, 152.5, 146.3, 142.4, 136.8, 136.1, 135.9, 131.7, 129.4, 127.7, 126.3, 123.4, 122.8, 122.6, 122.1, 119.8, 111.1, 109.7, 90.7, 54.2, 54, 53.2, 47.1, 31, 29.1, 29, 28.7, 28.6, 26.4, 23.7, 19.4, 11.9. HPLC retention time = 17.429 min. HRMS (ESI) *m*/*z* Calcd for C_35_H_43_N_4_O_2_ [M + H] ^+^: 551.3388, found 551.3359.

## Supplementary Material







## Notes

Docking scores, full Western blot gels and gel quantification, RNaseq data, **Cle-C8K** tissue distribution calibration plot, ^1^H- and ^13^C NMR spectra, HPLC chromatograms (PDF)
Docking PDBs (ZIP)
Molecular string file for the target compounds (CSV)

## Abbreviations

AR: androgen receptor
AURKA: Aurora kinase A
CADs: cationic amphiphilic drugs
CiA: chromatin in vivo assay
cIAP1: cellular inhibitor of apoptosis protein 1
cIAP2: cellular inhibitor of apoptosis protein 2
CIP: chemical induced proximity
Cle: Clemizole
CDKN1A/p21: cyclin-dependent kinase inhibitor 1
DFP: deferiprone
ERK 1/2: extracellular signal-regulated kinases 1/2
FOXM1: forkhead box protein M1
GOBP: gene ontology biological process
GSEA: gene set enrichment analysis
HRH1: histamine receptor H1
HDACs: histone deacetylases
KDMs: histone lysine demethylases
IP: intraperitoneal
JmjC: Jumonji C
MTD: maximum tolerated dose
MBG: metal binding group
mES: mouse embryonic stem
p-p38 MAPK: phosphorylated p38 mitogen-activated protein kinase
TNBC: triple negative breast cancer
XIAP: X-linked inhibitor of apoptosis protein
