# Supplementary material for: Repositioning Antihistamine for Cancer Therapy: Clemizole as a Template for the Design of Liver Tissue-Targeting Epigenetic-Modifying Agents
Source: J Med Chem. 2026 Jan 28;69(3):2238–57. doi: 10.1021/acs.jmedchem.5c02018 (PMC12910654; doi:10.1021/acs.jmedchem.5c02018)

Repositioning Antihistamine for Cancer Therapy: Clemizole as a Template for the Design of Liver Tissue-Targeting Epigenetic-Modifying Agents.

Dipak T. Walunj[1¶], Bocheng Wu[1¶], Jeremiah O. Olugbami[1¶], Alexis Johnston[1], Ryan Kern,[1] Travis J. Nelson[2], Benjamin H. Peer[1], Justin Keener,[1] Peixian He,[1] Nathaniel A Hathaway[2], Adegboyega K. Oyelere[1, 3*]

[1]School of Chemistry and Biochemistry, Georgia Institute of Technology, Atlanta, GA 30332-0400, USA; [2]The University of North Carolina Eshelman School of Pharmacy, Chapel Hill, NC, 27599, USA; [3]Parker H. Petit Institute for Bioengineering and Bioscience, Georgia Institute of Technology, Atlanta, GA 30332-0400, USA.

[*]Address correspondence to: Adegboyega K. Oyelere, PhD at email: aoyelere@gatech.edu or phone: 404-894-4047.

[¶]These authors contributed equally to the manuscript.

Keywords: Antihistamine; Histone deacetylase; Histone lysine demethylase; Clemizole; Deferiprone; RNA seq; Hepatocellular carcinoma.

Supplementary Information

Sr. No.	Table of Contents	Page No.
1	Table S1. Dock scores of the designed Cle-based HDACi and KDMi	S3
2	Figure S1. Representative full gels (a-b) of Hep-G2 cells treated with Cle-based HDACi	S4
3	Figure S2. Western blot analysis of histone H4 and α-tubulin acetylation status	S5
4	Figure S3: Structure of the previously disclosed compound VK-II-100, and TSA and SAHA.	S5
5	Figure S4. Effects of Cle-based HDACi on cell cycle progression in Hep-G2 cells	S6
6	Figure S5. Effects of Cle-based KDMi on cell cycle progression in Hep-G2 cells	S7
7	Figure S6: Representative full gels of Hep-G2 cells treated with Cle-based KDMi	S8
8	Figure S7. Densitometric quantifications (a - i) of Hep-G2 cells treated with Cle-based KDMi	S9-S11
9	Figure S8. Effects of Cle-C8K in Huh7 cells on the expression of selected genes from the hallmark hypoxia gene set	S11
9	Figure S9. Effects of Cle-C6K in Huh7 cells on the expression of selected genes from the hallmark hypoxia gene set	S12
10	Figure S10. Gene Ontology Biological Processes (GOBP) Gene Set Enrichment Analysis (GSEA) in Huh7 cells treated with Cle-C8K and Cle-C6K.	S13
11	Figure S11. Effects of Cle-C6K on the expression of standard gene set of selected oncogenes, tumor suppressors, and cell cycle inhibitors.	S14
12	Figure S12. Effects of Cle-C8K on the expression of genes probed for in the Western blot in Figure 7	S15
13	Figure S13. Effects of Cle-C6K on the expression of genes probed for in the Western blot in Figure 7	S15
14	Figure S14. Calibration plot for the Cle-C8K standard, tissue distribution and statistical analysis of the MTD data.	S15-S16
15	^{1}H and ^{13}C NMR Spectra	S17-S33
16	HPLC-UV Tracers	S34-S36

Enzyme	Compound name	Binding affinity (kcal/mol)
HDAC2 (PDB: 3MAX)	**Cle-C6**	-6.6
	**Cle-C7**	-7.9
	**Cle-C8**	-5.8
	**Cle-PH**	-8.9
	**Cle-PPH**	-9.4
HDAC6 (PDB: 5G0J)	**Cle-C6**	-7.2
	**Cle-C7**	-7.8
	**Cle-C8**	-7.6
	**Cle-PH**	-10.4
	**Cle-PPH**	-7.2
KDM6A (PDB: 3AVR)	**Cle-C4K**	-9.4
	**Cle-C5K**	-9.0
	**Cle-C6K**	-8.2
	**Cle-C7K**	-8.8
	**Cle-C8K**	-8.4
	**Cle-AC-6K**	-7.3
	**Cle-AC-7K**	-7.6
	**Cle-AC-8K**	-8.1
KDM5B (PDB: 6H4Z)	**Cle-C4K**	-10.8
	**Cle-C5K**	-11.0
	**Cle-C6K**	-10.8
	**Cle-C7K**	-11.1
	**Cle-C8K**	-11.4
	**Cle-AC-6K**	-11.0
	**Cle-AC-7K**	-10.4
	**Cle-AC-8K**	-10.6

Supplementary Table S1. Dock scores of the designed Cle-based HDACi and KDMi. Selected docked outputs were based on orientations that maintained optimal chelation with the enzymes' active site metal ions.

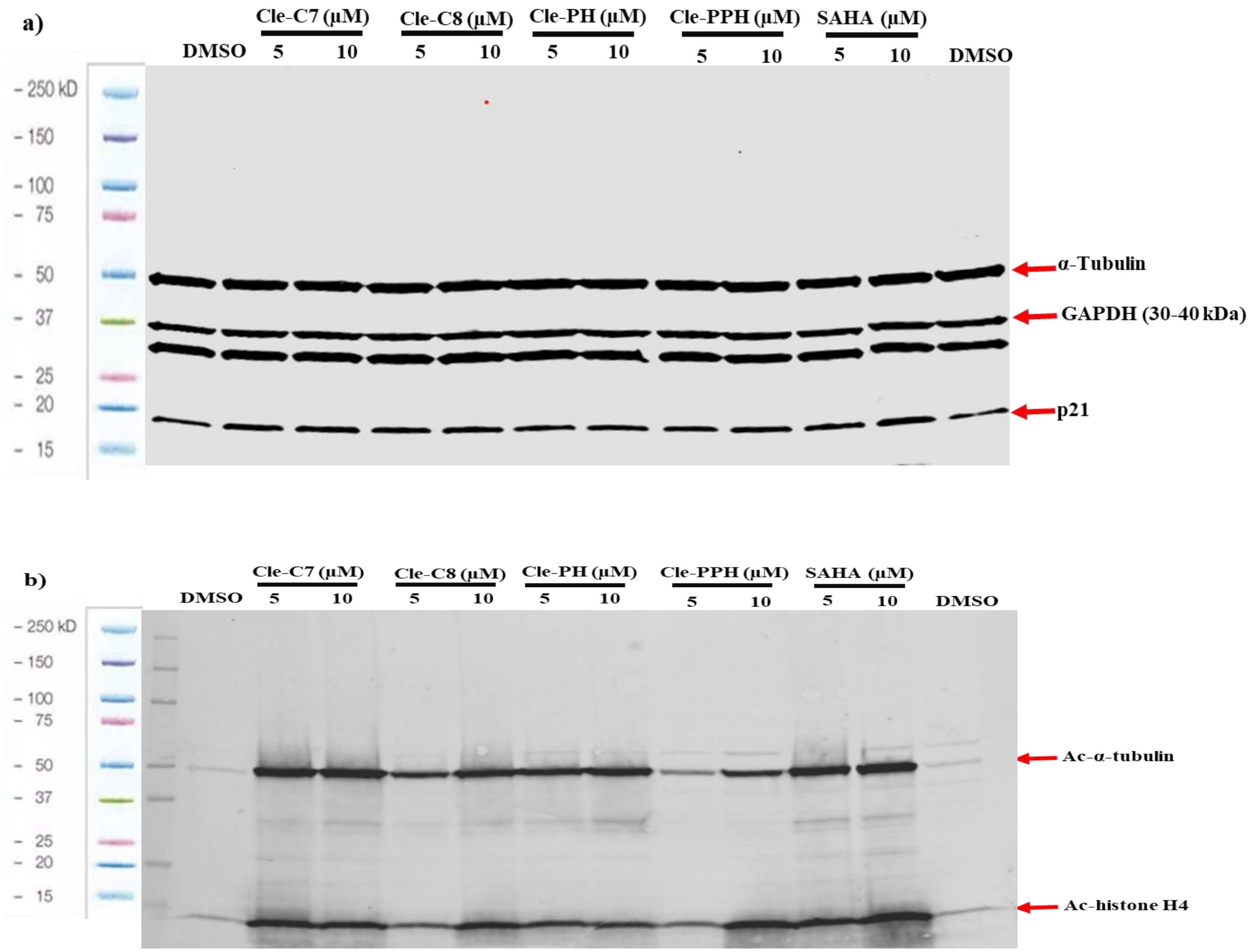

Figure S1: Representative full gels (a-b) of Hep-G2 cells treated with Cle-based HDACi for 4 h as indicated above.

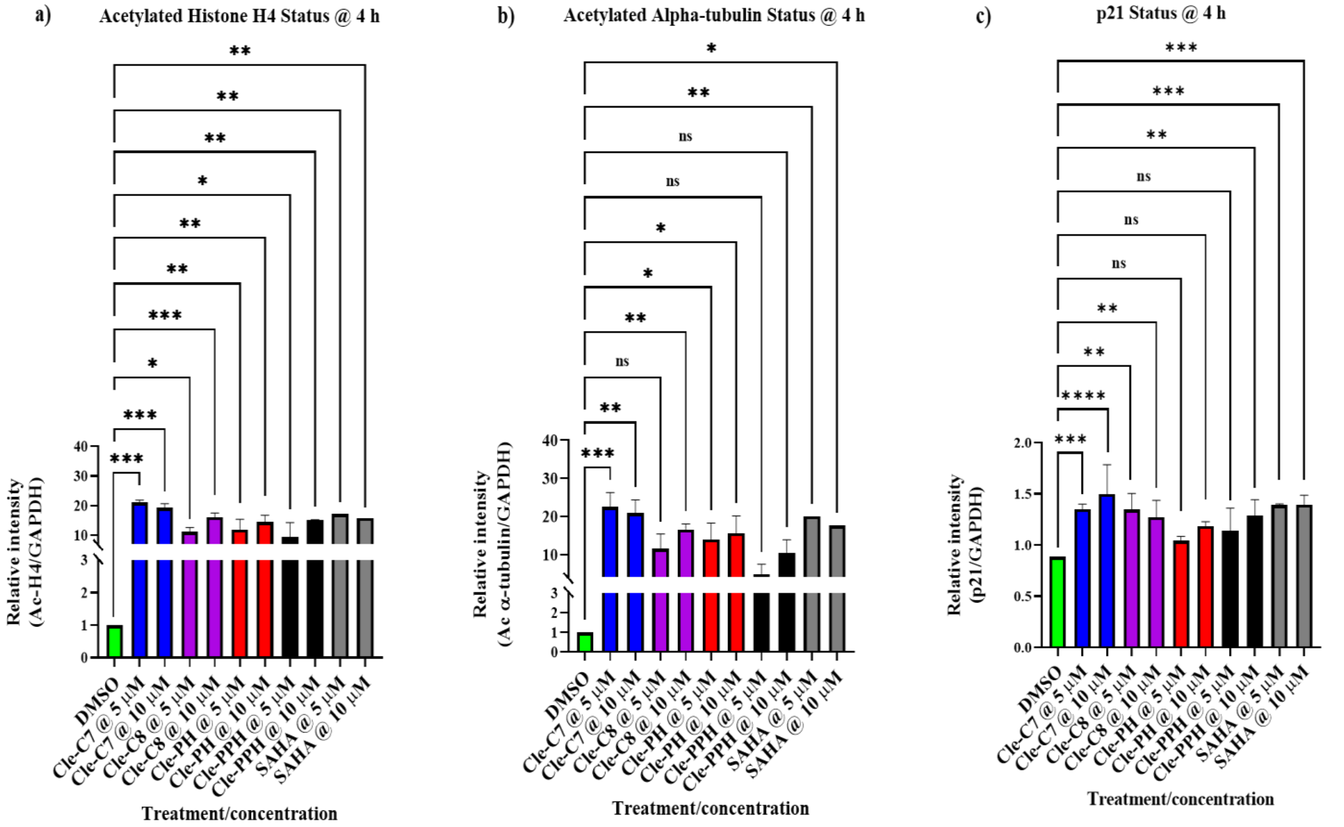

Figure S2. Western blot analysis of histone H4 and α-tubulin acetylation status. Hep-G2 cells were treated with 1% DMSO or 1% DMSO solutions of the test compounds:– **Cle-C7** (5 and 10 μM), **Cle-C8** (5 and 10 μM), **Cle-PH** (5 and 10 μM), **Cle-PPH** (5 and 10 μM), and SAHA (5 and 10 μM) – for 4 h. Densitometric quantifications (a, b, and c) of the immunoblot obtained. The quantifications are based on at least two independent experiments. (Bars show means plus standard deviations; *P < 0.05; **P < 0.001; ***P < 0.0001).

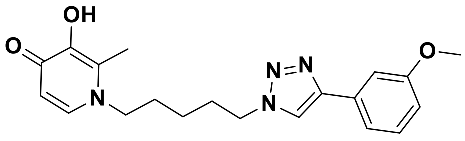

VK-II-100

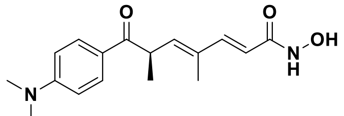

Trichostatin A (TSA)

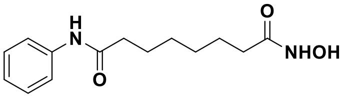

SAHA

Figure S3: Structure of the previously disclosed compound **VK-II-100**, and TSA and SAHA.

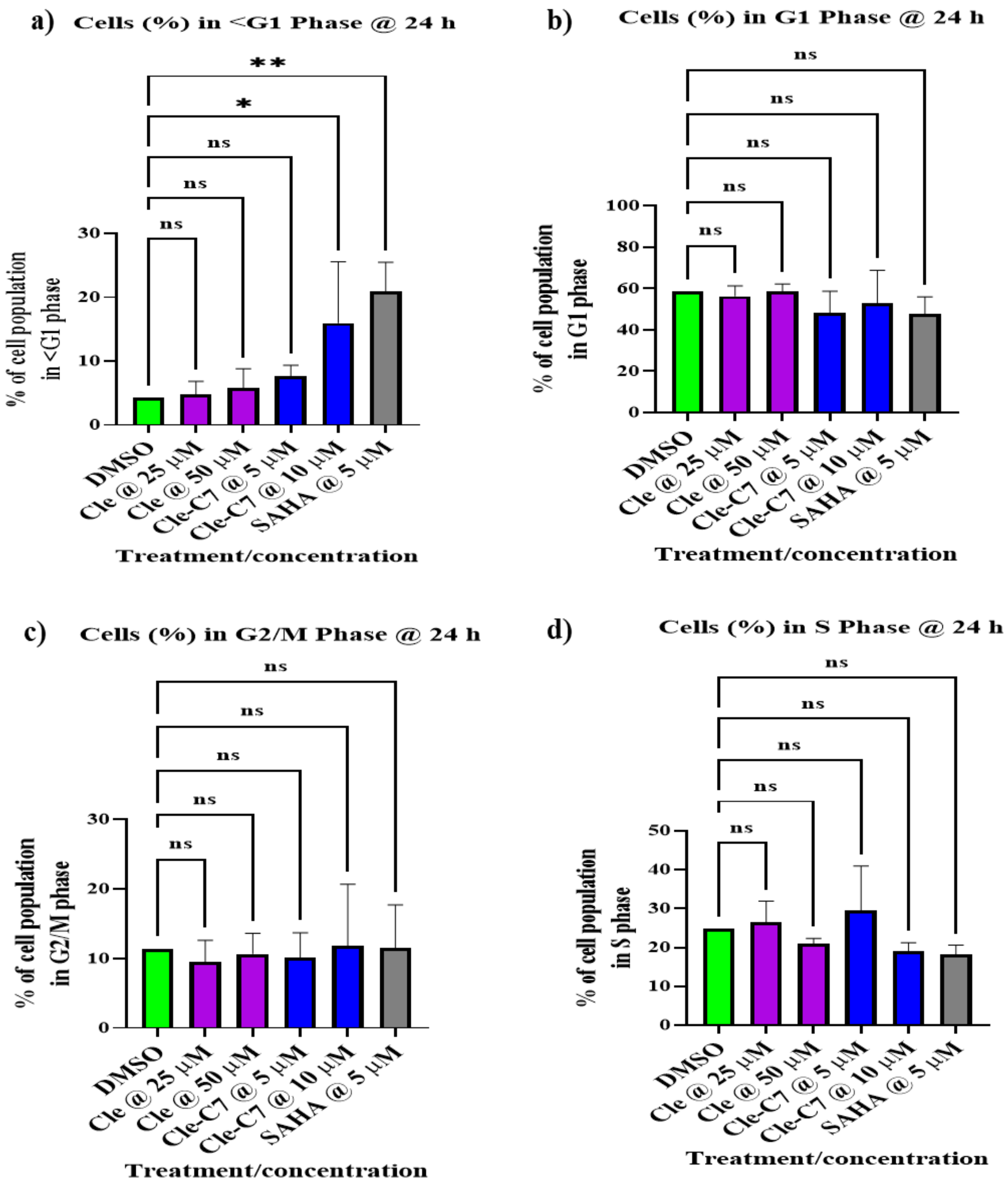

Figure S4. Effects of Cle-based HDACi on cell cycle progression in Hep-G2 cells treated for 24 h. Panels a-d show bar charts representing the distinct cell cycle phases and the attendant effects of various treatments on each of the phases. Cle-based HDACi induced apoptosis, and cell cycle arrest at both S- and G2/M-phases. The quantifications are based on at least two independent experiments. (Bars show means plus standard deviations; $*P < 0.05$; $**P < 0.001$).

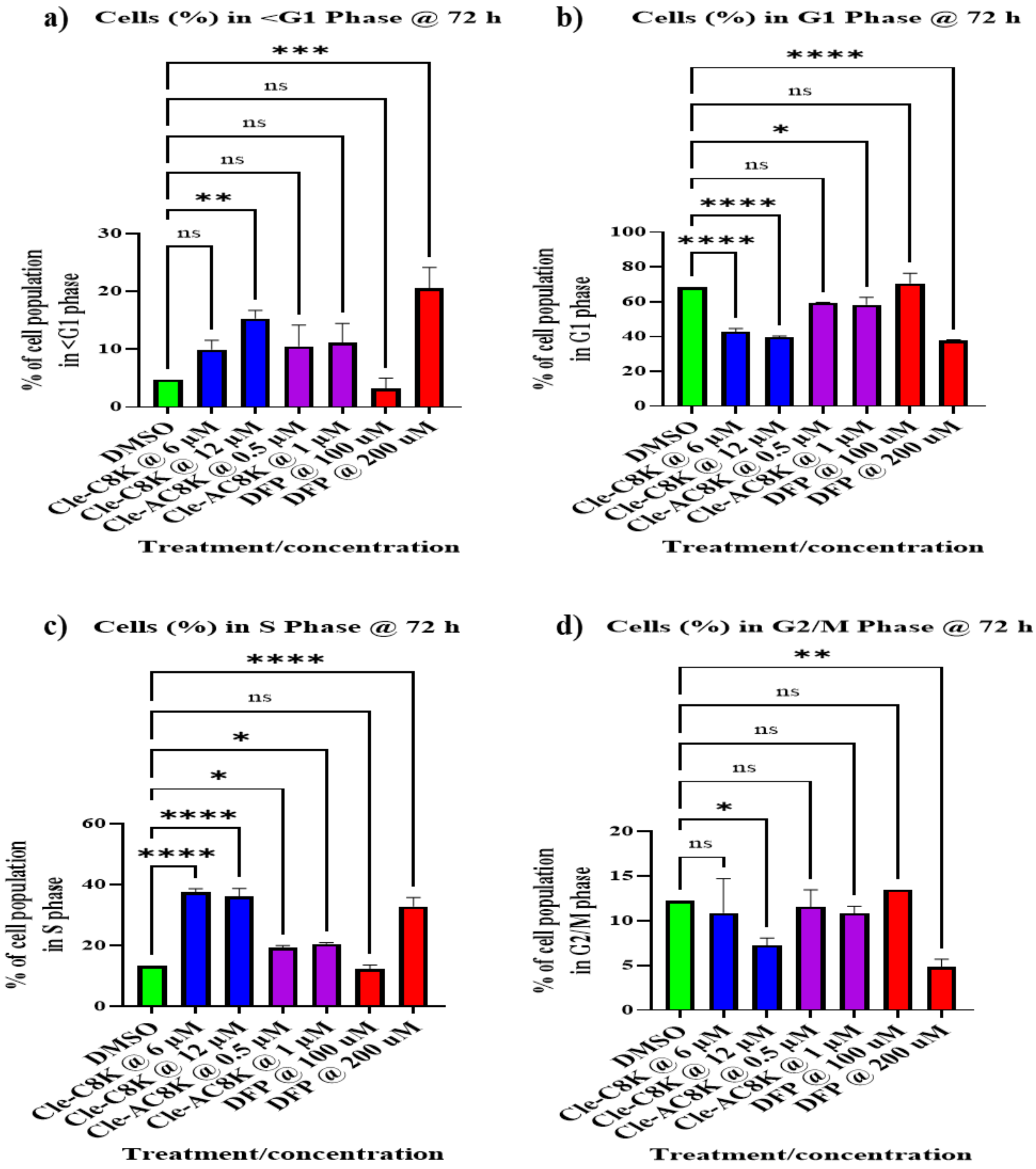

Figure S5. Effects of Cle-based KDMi on cell cycle progression in Hep-G2 cells after treatment for 72 h. Panels a-d show bar charts representing the distinct cell cycle phases and the attendant effects of various treatments on each of the phases. Cle-based KDMi induced apoptosis, and significantly increased cells in the S-phase. The quantifications are based on at least two independent experiments. (Bars show means plus standard deviations; *P < 0.05; **P < 0.003; ***P/****P < 0.0001).

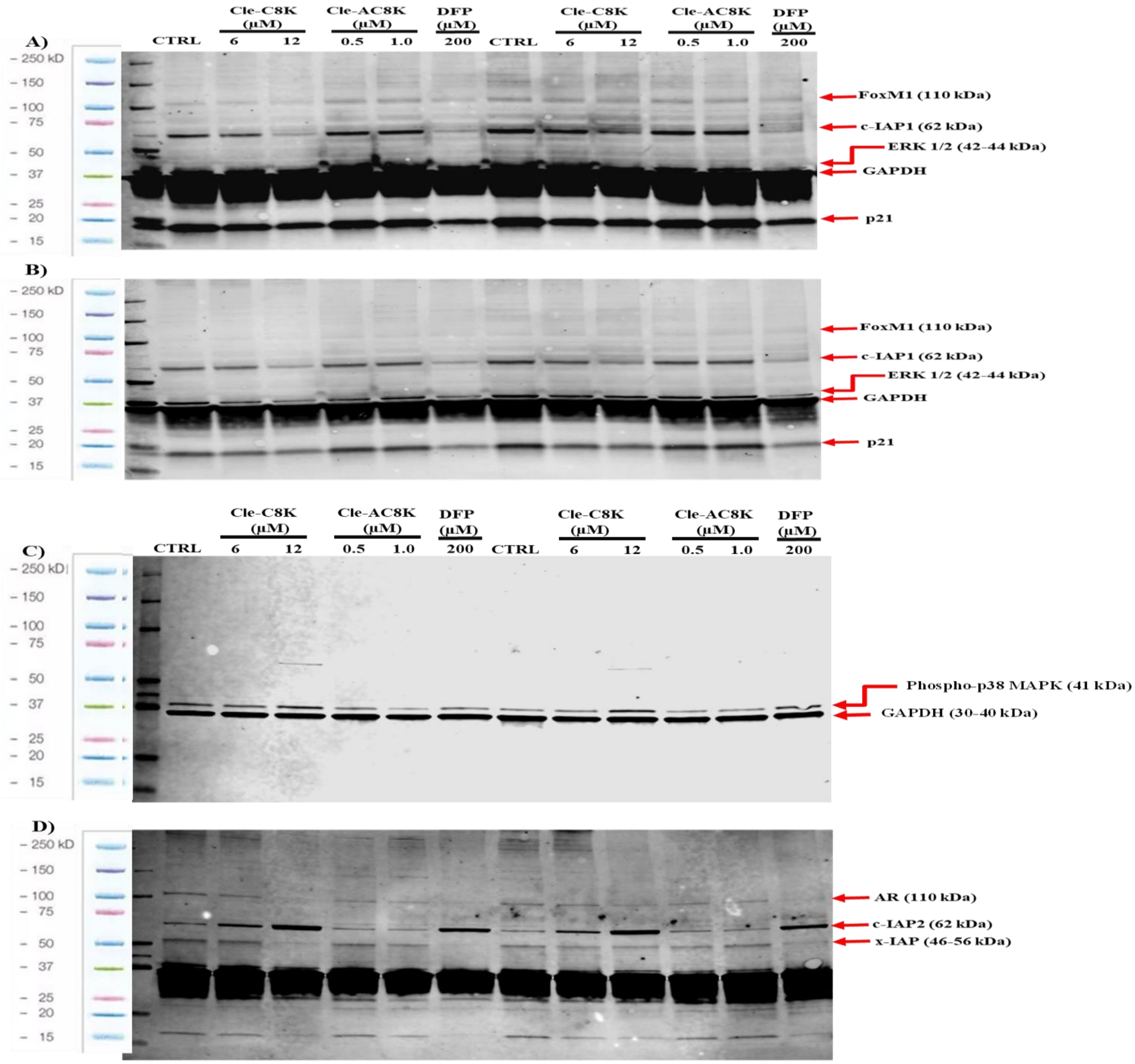

Figure S6: Representative full gels of Hep-G2 cells treated with Cle-based KDMi for 72 h, as indicated above.

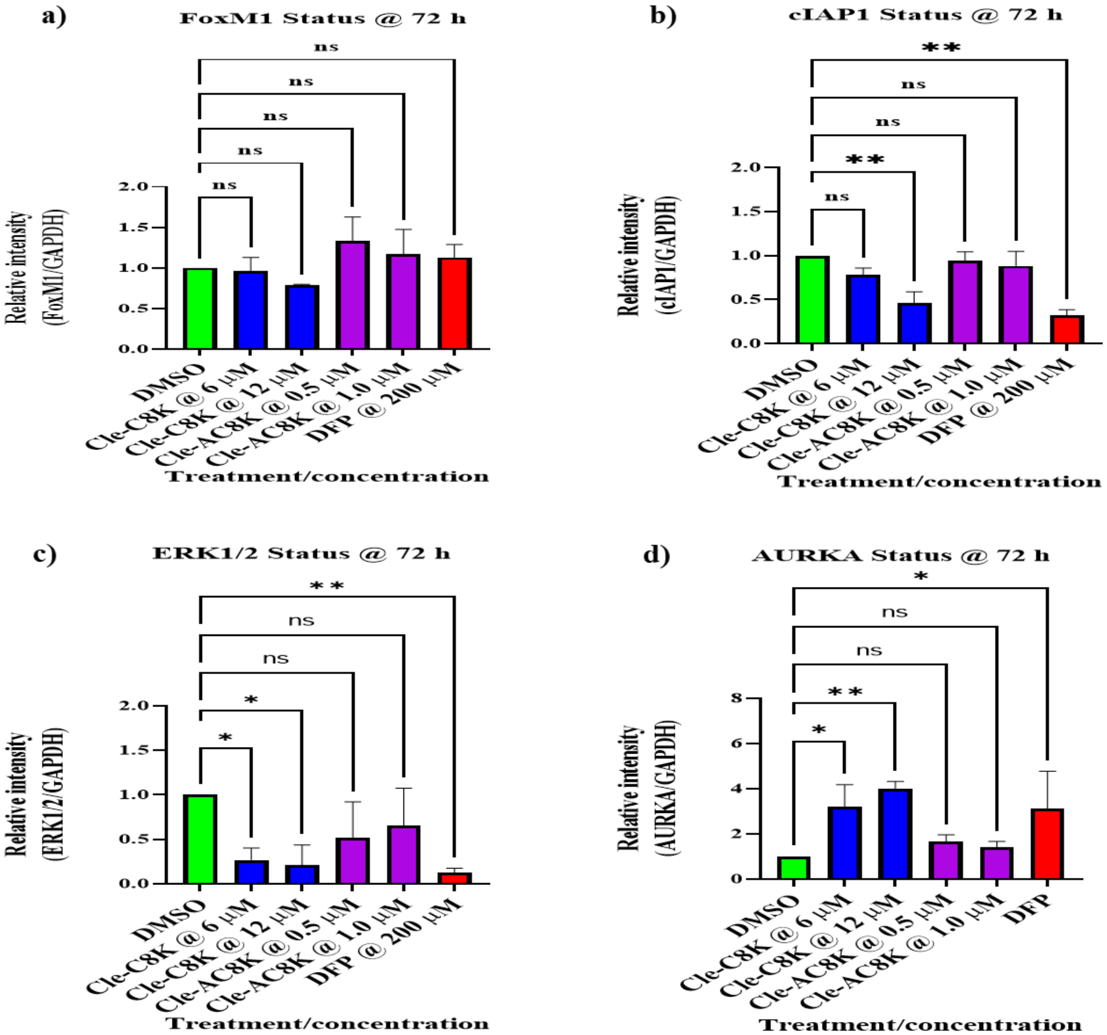
a)
FoxM1 Status @ 72 h
Relative intensity (FoxM1/GAPDH)
ns
2.0
1.5
1.0
0.5
0.0
DMSO
Cle-C8K @ 6 μM
Cle-C8K @ 12 μM
Cle-AC8K @ 0.5 μM
Cle-AC8K @ 1.0 μM
DFP @ 200 μM
Treatment/concentration
b)
cIAP1 Status @ 72 h
Relative intensity (cIAP1/GAPDH)
**
ns
DMSO
Cle-C8K @ 6 μM
Cle-C8K @ 12 μM
Cle-AC8K @ 0.5 μM
Cle-AC8K @ 1.0 μM
DFP @ 200 μM
Treatment/concentration
c)
ERK1/2 Status @ 72 h
Relative intensity (ERK1/2/GAPDH)
**
ns
*
DMSO
Cle-C8K @ 6 μM
Cle-C8K @ 12 μM
Cle-AC8K @ 0.5 μM
Cle-AC8K @ 1.0 μM
DFP @ 200 μM
Treatment/concentration
d)
AURKA Status @ 72 h
Relative intensity (AURKA/GAPDH)
*
ns
**
8
6
4
2
0
DMSO
Cle-C8K @ 6 μM
Cle-C8K @ 12 μM
Cle-AC8K @ 0.5 μM
Cle-AC8K @ 1.0 μM
DFP
Treatment/concentration

e)

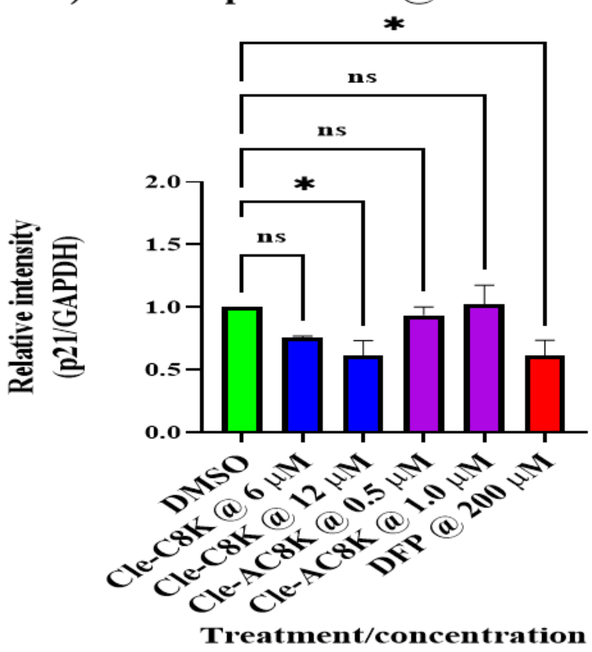
p21 Status @ 72 h
*
ns
ns
*
ns
2.0
1.5
1.0
0.5
0.0
Relative intensity
(p21/GAPDH)
DMSO
Cle-C8K @ 6 μM
Cle-C8K @ 12 μM
Cle-AC8K @ 0.5 μM
Cle-AC8K @ 1.0 μM
DFP @ 200 μM
Treatment/concentration

f)

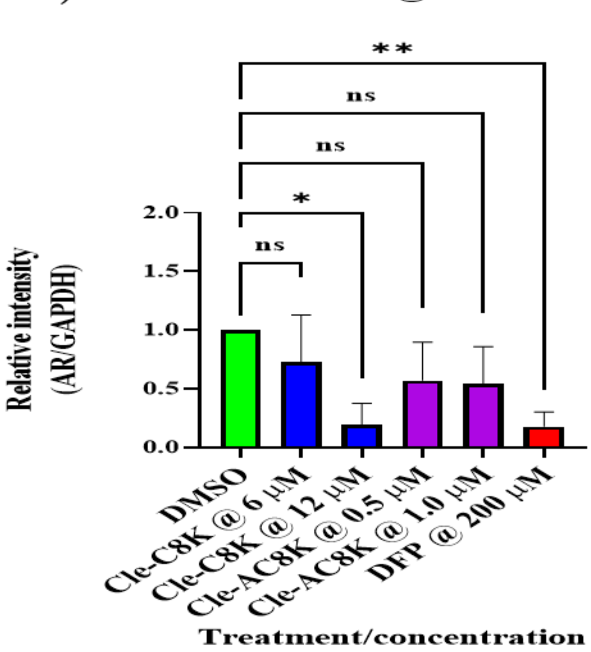
AR Status @ 72 h
**
ns
ns
*
ns
2.0
1.5
1.0
0.5
0.0
Relative intensity
(AR/GAPDH)
DMSO
Cle-C8K @ 6 μM
Cle-C8K @ 12 μM
Cle-AC8K @ 0.5 μM
Cle-AC8K @ 1.0 μM
DFP @ 200 μM
Treatment/concentration

g)

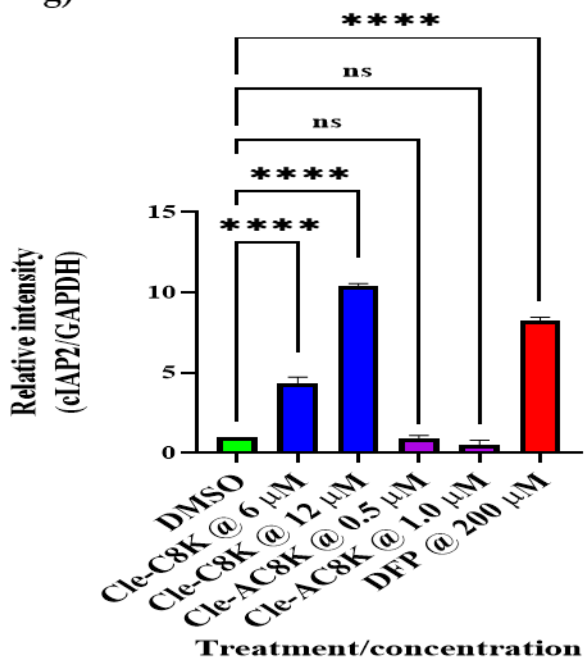
cIAP2 Status @ 72 h
****
ns
ns
****
****
15
10
5
0
Relative intensity
(cIAP2/GAPDH)
DMSO
Cle-C8K @ 6 μM
Cle-C8K @ 12 μM
Cle-AC8K @ 0.5 μM
Cle-AC8K @ 1.0 μM
DFP @ 200 μM
Treatment/concentration

h)

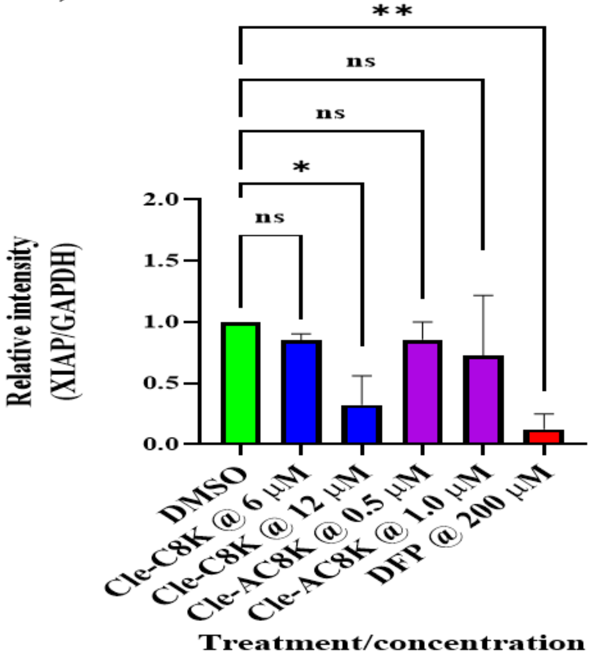
XIAP Status @ 72 h
**
ns
ns
*
ns
2.0
1.5
1.0
0.5
0.0
Relative intensity
(XIAP/GAPDH)
DMSO
Cle-C8K @ 6 μM
Cle-C8K @ 12 μM
Cle-AC8K @ 0.5 μM
Cle-AC8K @ 1.0 μM
DFP @ 200 μM
Treatment/concentration

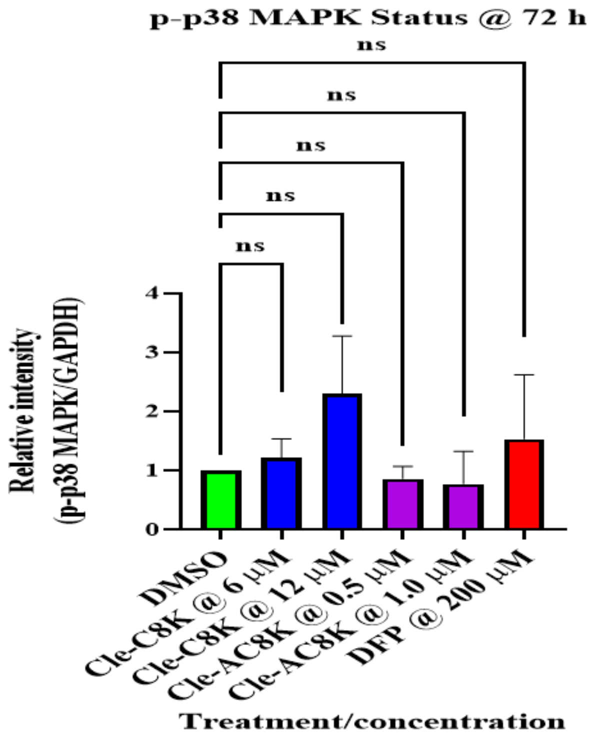

Figure S7. Densitometric quantifications (a - i) of Hep-G2 cells treated with Cle-based KDMi for 72 h, as indicated above. The quantifications are based on at least two independent experiments. (Bars show means plus standard deviations; *P < 0.05; **P < 0.01; ****P < 0.0001).

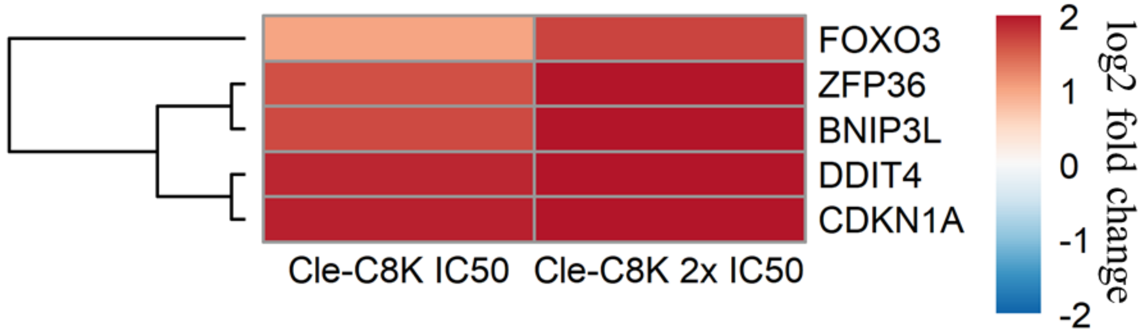

Figure S8. Effects of **Cle-C8K** in Huh7 cells on the expression of selected genes from the hallmark hypoxia gene set. Log2 fold change heatmap displaying upregulation of the selected genes by **Cle-C8K** at both 0.5µM (IC_{50}) and 1µM (2x IC_{50}), FOXO3 (log2 fold change = +1.0, +1.7), ZFP36 (log2 fold change +1.6, +2.1), BNIP3L (log2 fold change +1.7, +2.2), DDIT4 (log2 fold change +1.9, +2.4), and CDKN1A (p21) (log2 fold change +1.9, +2.4).

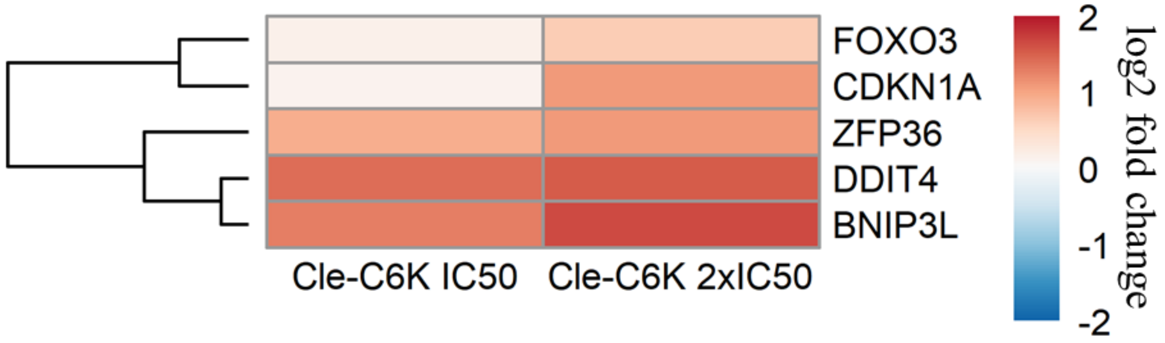

Figure S9. Effects of **Cle-C6K** in Huh7 cells on the expression of selected genes from the hallmark hypoxia gene set. Log2 fold change heatmap displaying upregulation of the selected genes by **Cle-C6K** at both 2.5µM (IC_{50}) and 5µM (2x IC_{50}), FOXO3 (log2 fold change +0.2, +0.6), CDKN1A (p21) (log2 fold change +0.1, +1.1), ZFP36 (log2 fold change +0.9, +1.1), DDIT4 (log2 fold change +1.4, +1.5), and BNIP3L (log2 fold change +1.3, +1.7).

a)

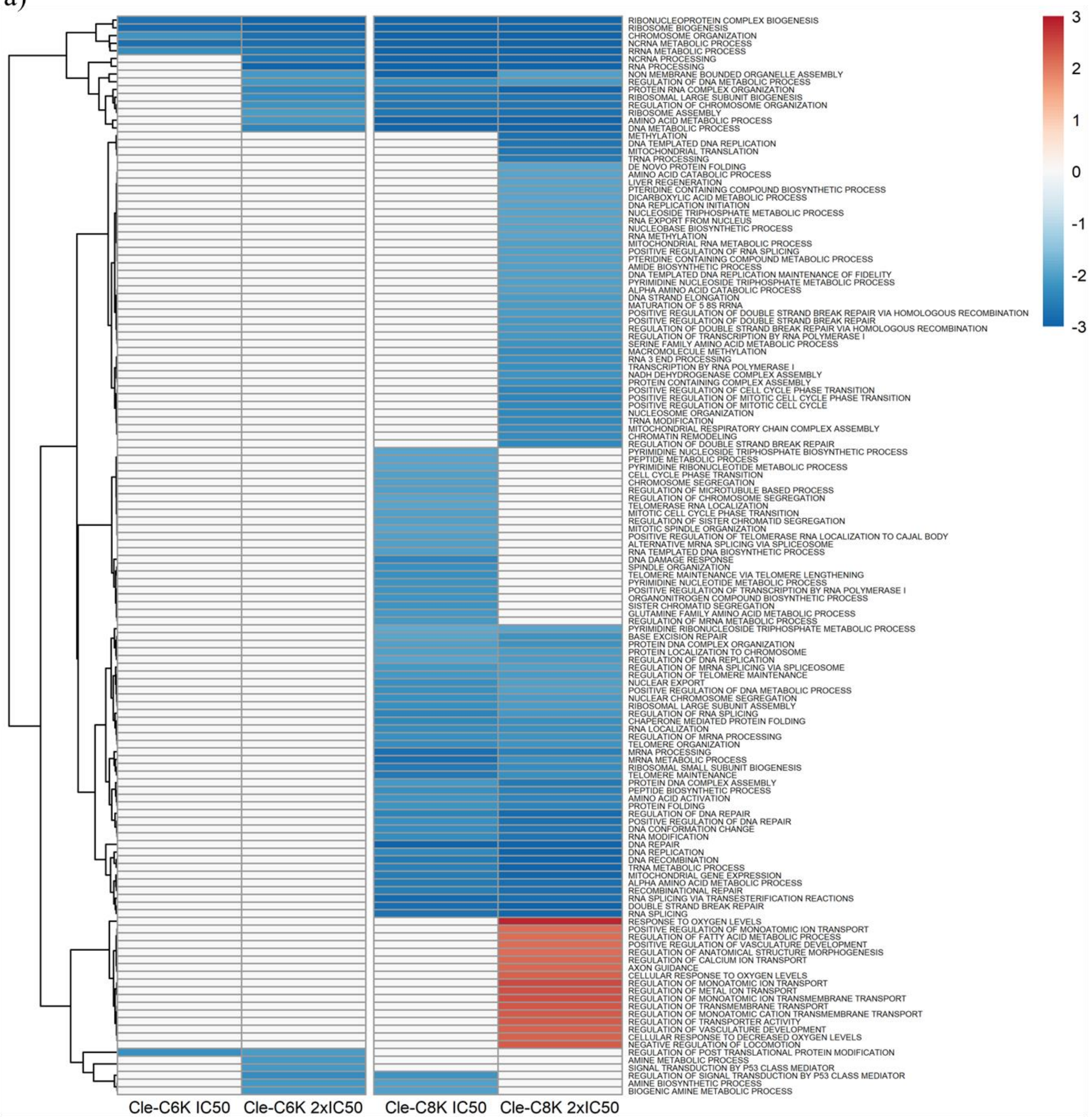

b)

GOBP Regulation of Post-Translational Protein Modification

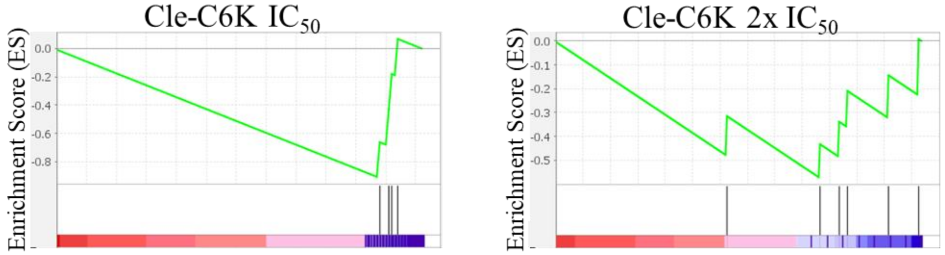

Figure S10. Gene Ontology Biological Processes (GOBP) Gene Set Enrichment Analysis (GSEA) in Huh7 cells treated with **Cle-C8K** and **Cle-C6K**. a) Heatmap of normalized enrichment scores (NES) of significantly enriched GOBP gene sets resulting from treatment with **Cle-C8K** at 0.5µM (IC_{50}) and 1µM (2x IC_{50}) and **Cle-C6K** at 2.5µM (~IC_{50}) and 5µM (~2 x IC_{50}) ($p < 0.01$, FDR < 0.05). b) Regulation of post-translational protein modification enrichment plot for **Cle-C6K** at 2.5µM (NES = -2.2) and 5µM (NES = -2.1).

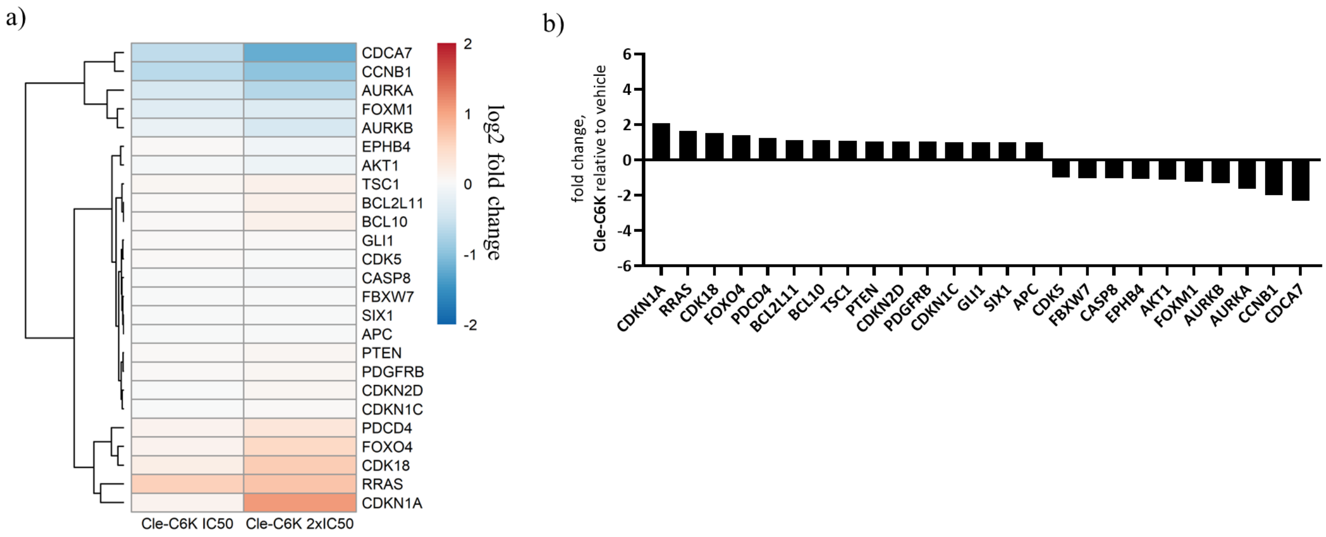

Figure S11. Effects of **Cle-C6K** on the expression of standard gene set of selected oncogenes, tumor suppressors, and cell cycle inhibitors. a) Log2 fold change heatmap of genes implicated in the KDM inhibition displaying upregulation of CDKN1A (p21) by **Cle-C6K** at 5 µM (log2 fold change = +1.1) and downregulation of CCNB1 (log2 fold change = -1.0, 5 µM). b) Fold change bar graph of **Cle-C6K** at 5 µM relative to DMSO.

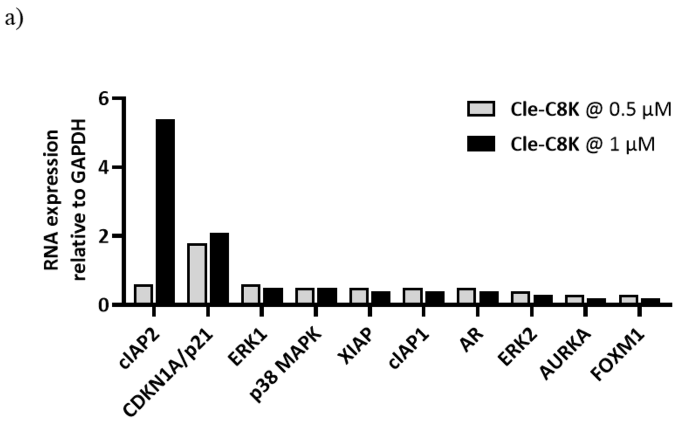

b)

GeneID	CleC8K IC50	CleC8K 2xIC50
cIAP2	2.0E-02	9.1E-04
CDKN1A/p21	1.3E-07	2.1E-10
ERK1	2.7E-02	3.4E-02
p38 MAPK	ns	ns
XIAP	ns	ns
cIAP1	ns	ns
AR	ns	ns
ERK2	ns	ns
AURKA	3.6E-06	2.5E-04
FOXM1	1.0E-03	8.9E-05

Figure S12. Effects of **Cle-C8K** on the expression of genes probed for in the Western blot in Figure 7. RNA relative expression level bar graph of the probed genes normalized to GAPDH

expression for **Cle-C8K** at 0.5μM (gray) and **Cle-C8K** at 1μM (black) displaying a 5.4-fold increase in cIAP2 expression (log2 fold change = +3.8), a 2.1-fold increase in CDKN1A/p21 expression (log2 fold change +2.4), and a decrease in XIAP, AR, ERK1, and ERK2 expression by **Cle-C8K** at 1μM. b) DESeq2 p values of gene expression levels relative to GAPDH expression. ns = not significant (p>0.05).

a)

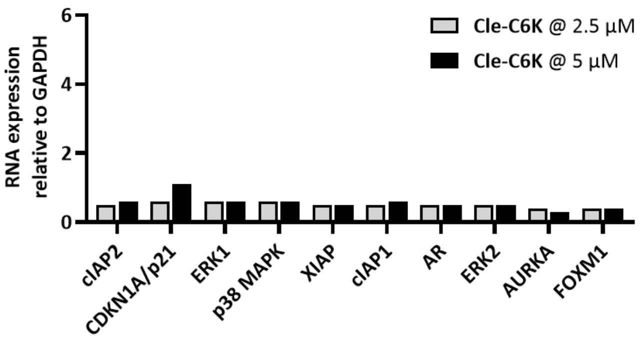

b)

GeneID	CleC6K IC50	CleC6K 2xIC50
cIAP2	ns	ns
CDKN1A/p21	ns	2.4E-03
ERK1	ns	ns
p38 MAPK	ns	ns
XIAP	ns	ns
cIAP1	ns	ns
AR	ns	ns
ERK2	ns	ns
AURKA	6.3E-03	1.7E-04
FOXM1	3.4E-02	4.1E-02

Figure S13. Effects of **Cle-C6K** on the expression of genes probed for in the Western blot in Figure 7. RNA relative expression level bar graph of the probed genes normalized to GAPDH expression for **Cle-C6K** at 2.5μM (gray) and **Cle-C6K** at 5μM (black) displaying a 1.1-fold increase in CDKN1A/p21 expression (log2 fold change = +1.1) by **Cle-C6K** at 5μM and a decrease in the expression of cIAP1, cIAP2, ERK1, ERK2, p38 MAPK, AURKA, and FOXM1. b) DESeq2 p values of gene expression levels relative to GAPDH expression. ns = not significant (p>0.05).

a)

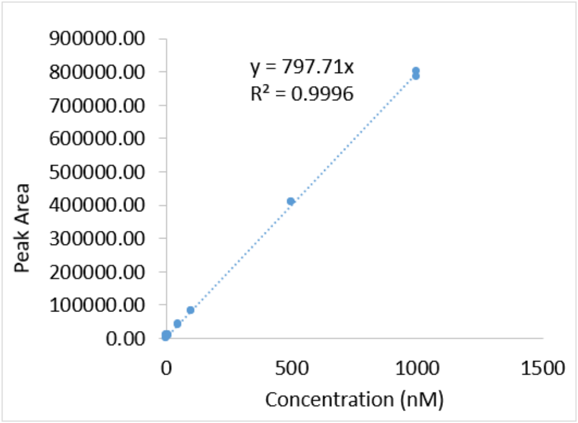

b)

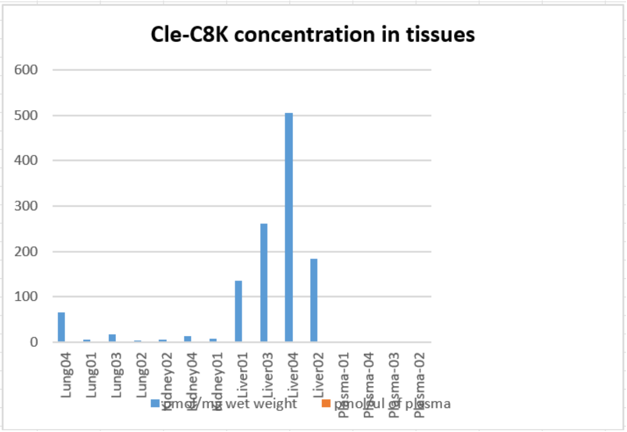

c)

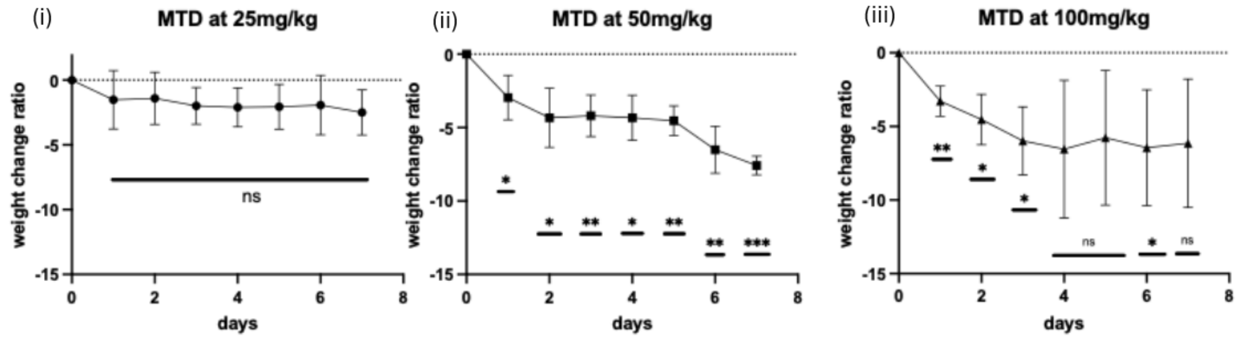

Figure S14. a) Calibration plot for the **Cle-C8K** standard. b) Concentrations (pmol/mg weight) of **Cle-C8K** at 50 mg/kg dosage in the lung, kidney, liver, and plasma of each C57BL/6 mouse (n = 4). c) Statistical analysis of the MTD data, p-value >0.1234 = ns; <0.0332 = *; <0.0021= **; <0.0002 = ***. Large variation in the 100 mg/kg cohort is due to one outlier mouse.

NMR Spectra

^{1}H (700 MHz, $CDCl_3$) and ^{13}C (175 MHz, $CDCl_3$) NMR spectrum of compound 2

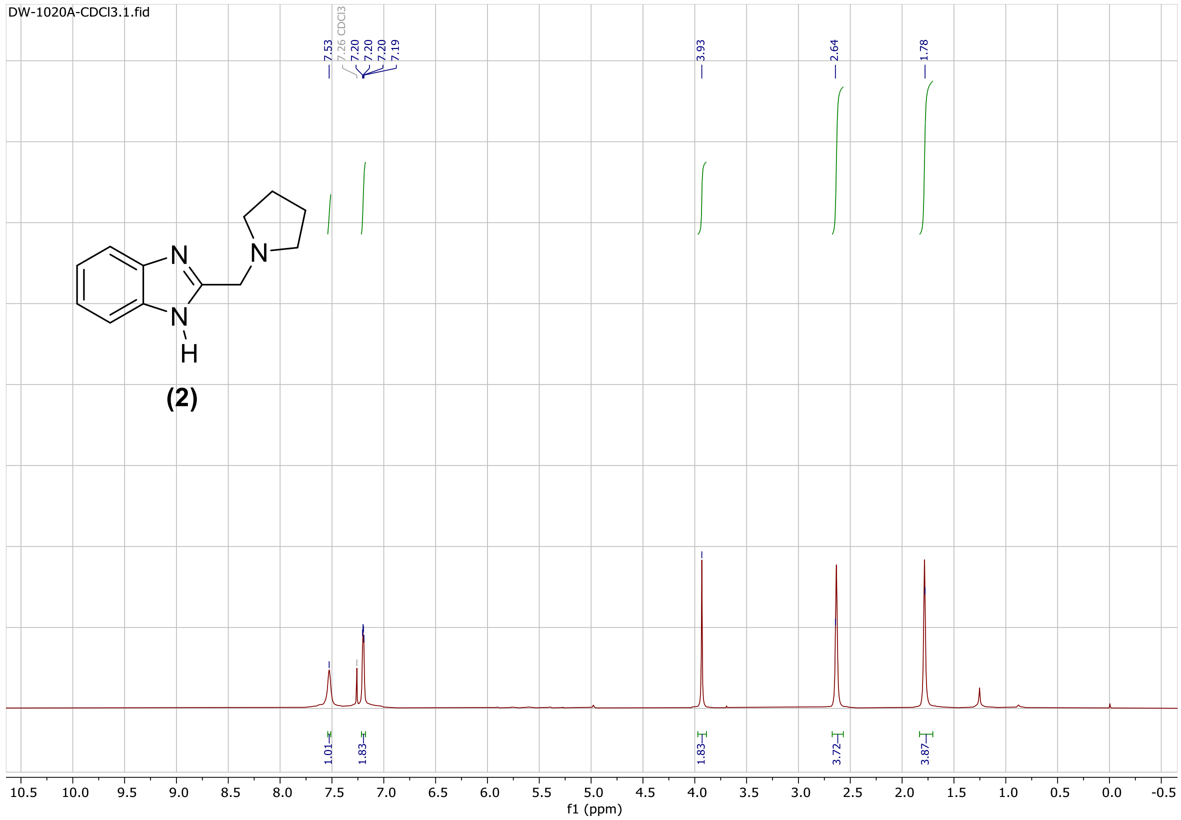

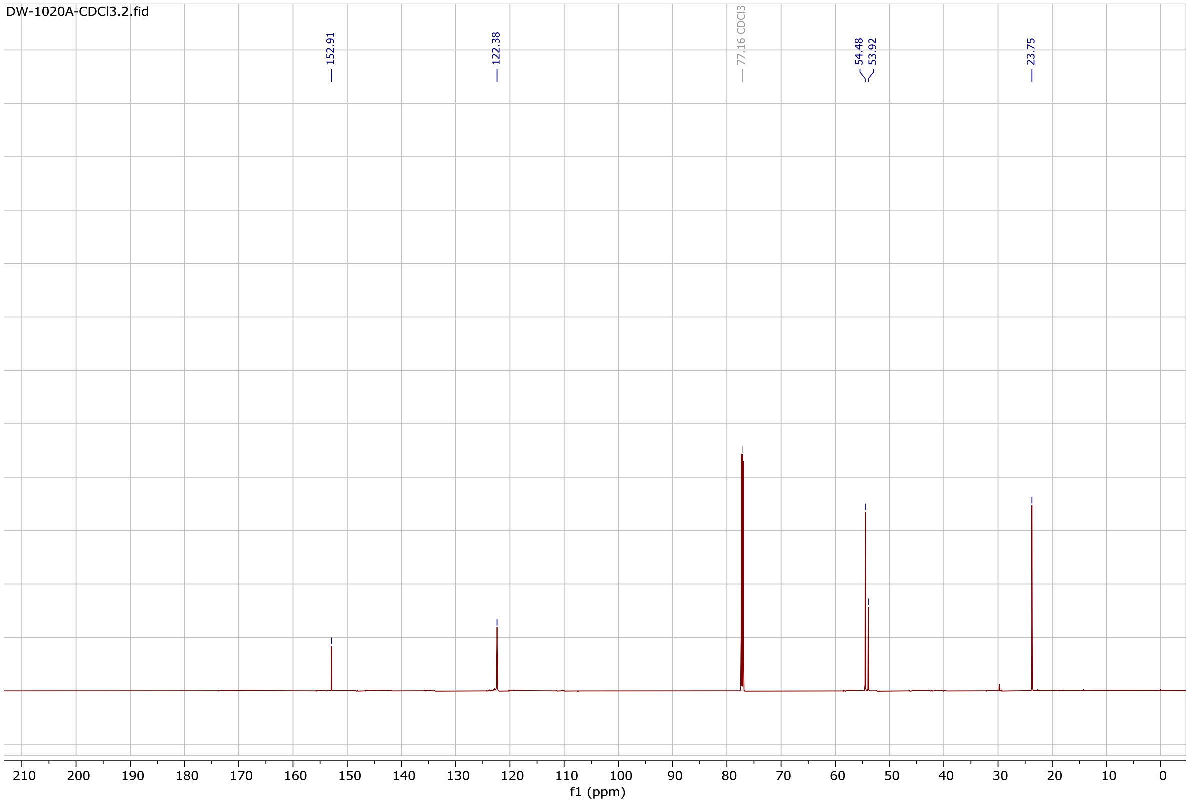

^{1}H (700 MHz, $CDCl_3$) and ^{13}C (175 MHz, $CDCl_3$) NMR spectrum of compound 3

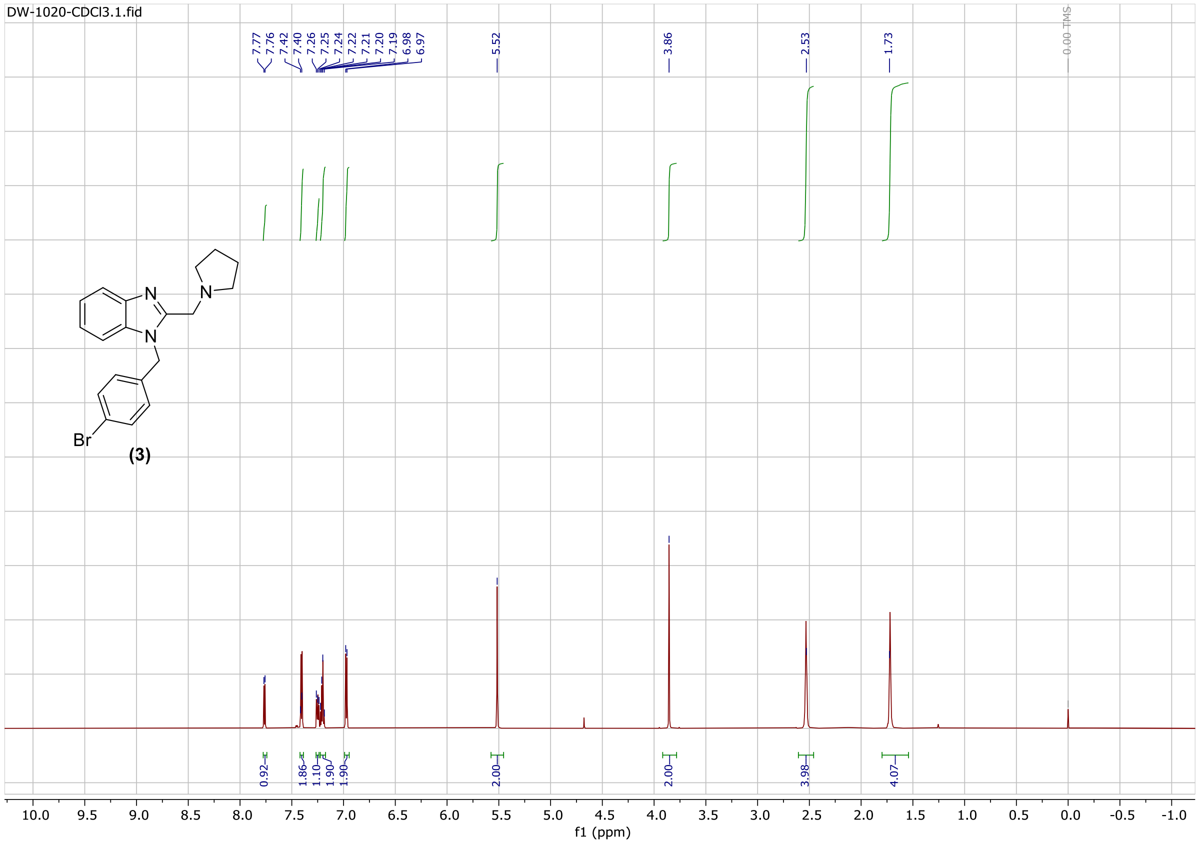

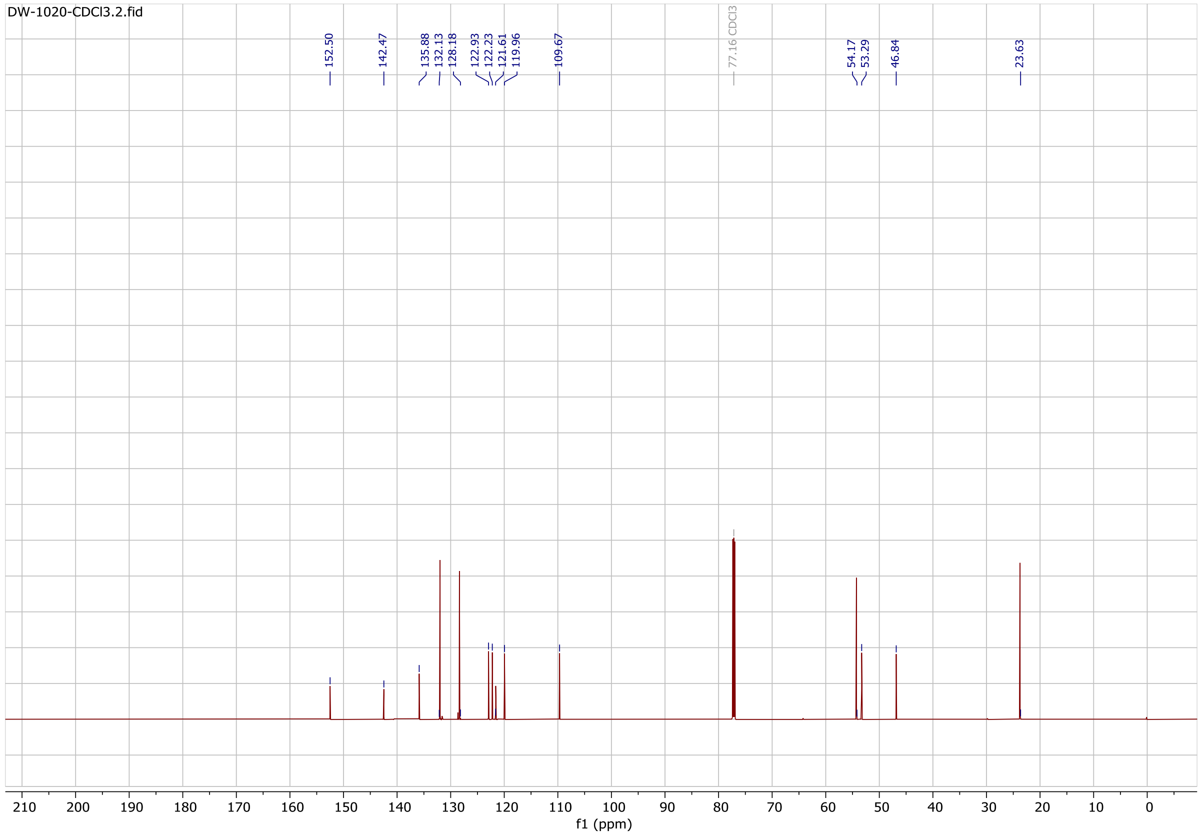

^{1}H (700 MHz, $CDCl_3$) and ^{13}C (175 MHz, $CDCl_3$) NMR spectrum of compound Cle-Alk (5)

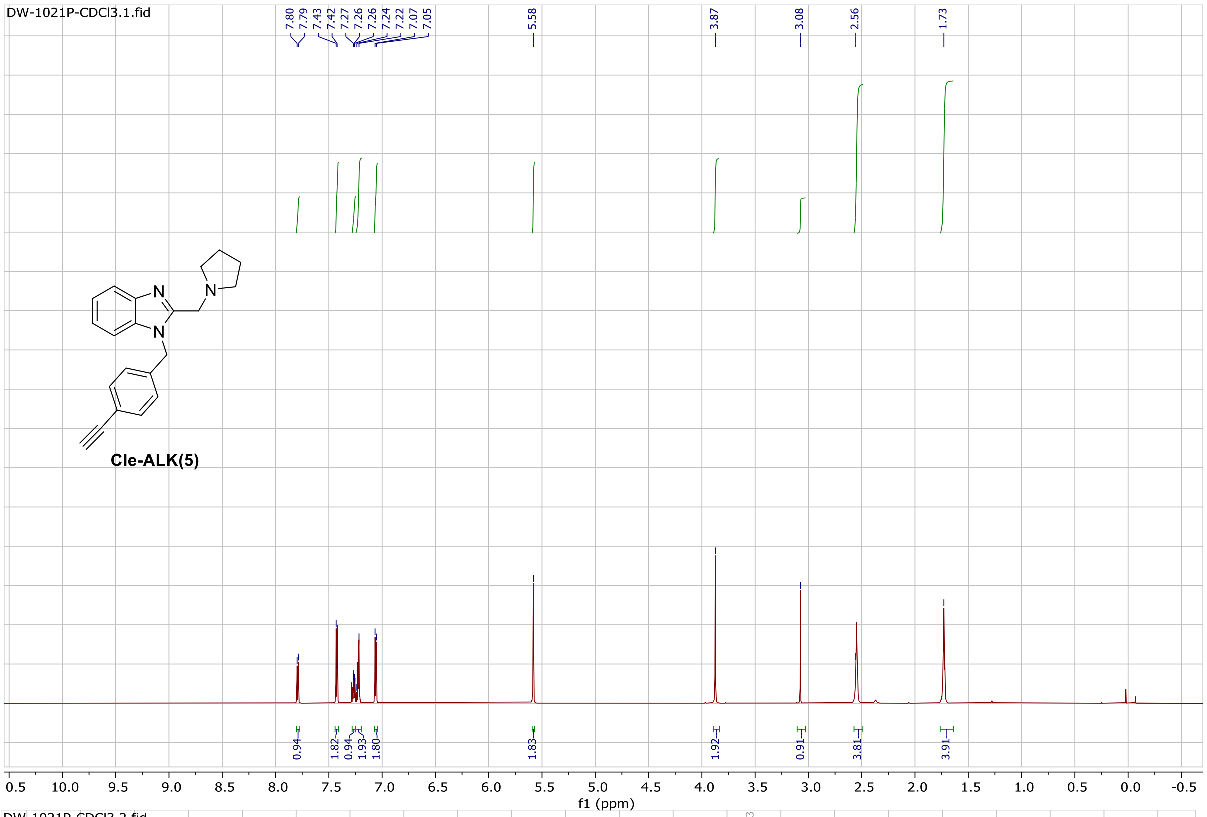

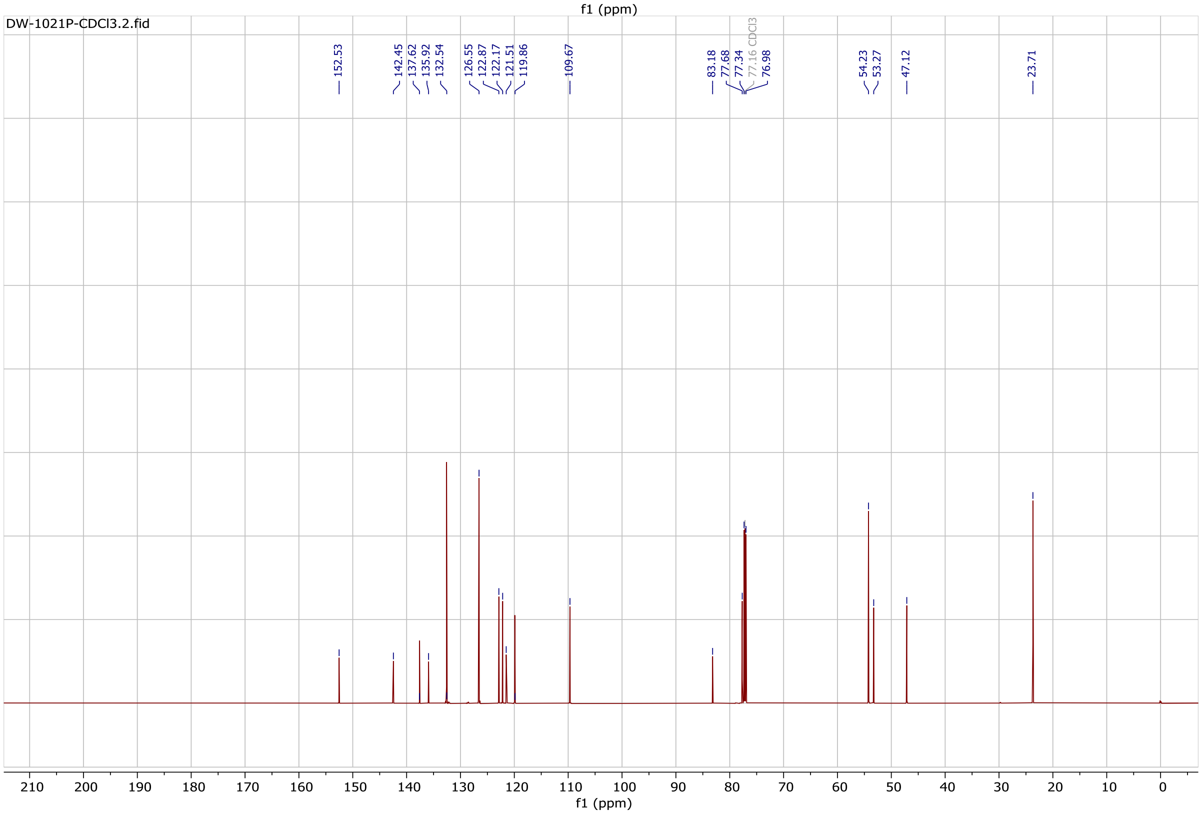

^{1}H (500 MHz, CD_3OD) and ^{13}C (175 MHz, CD_3OD) NMR spectrum of compound Cle-C6

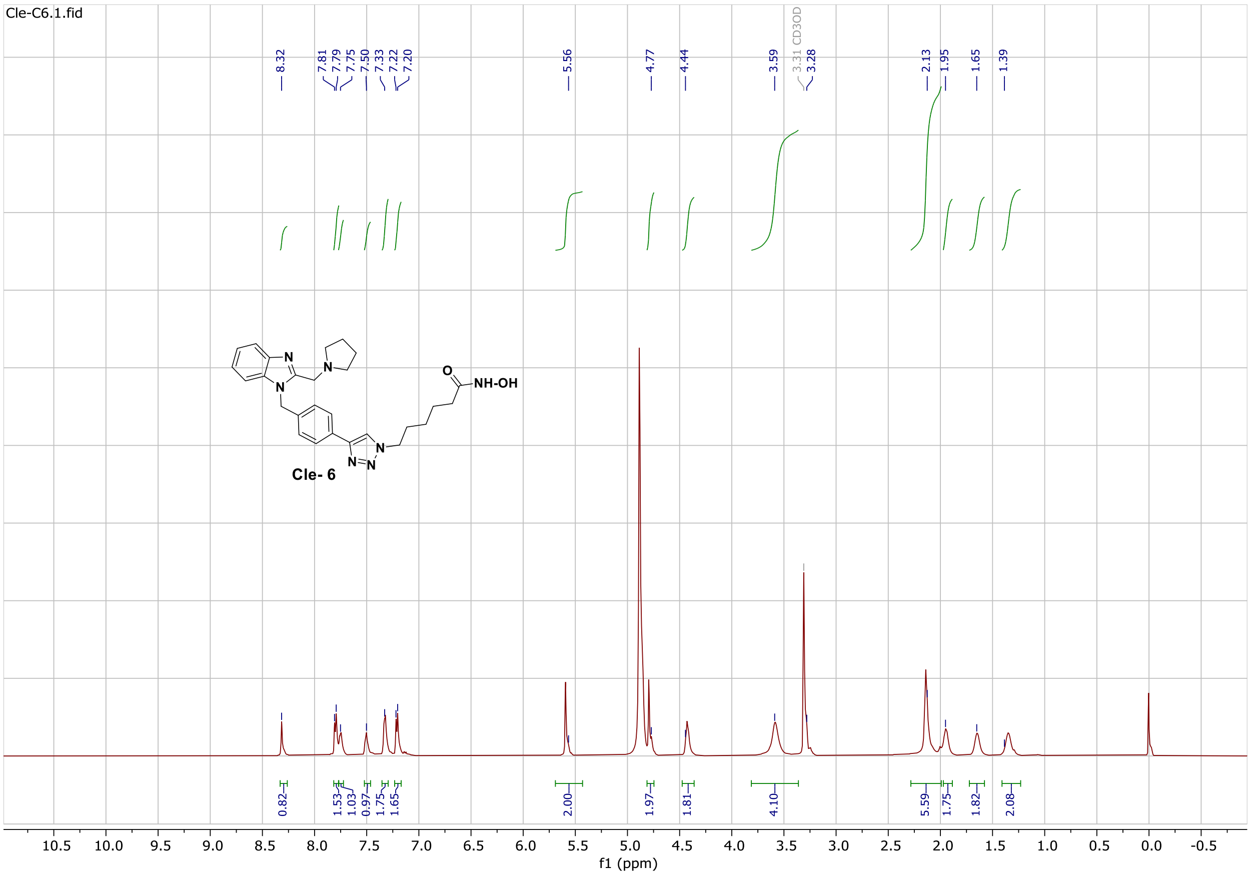

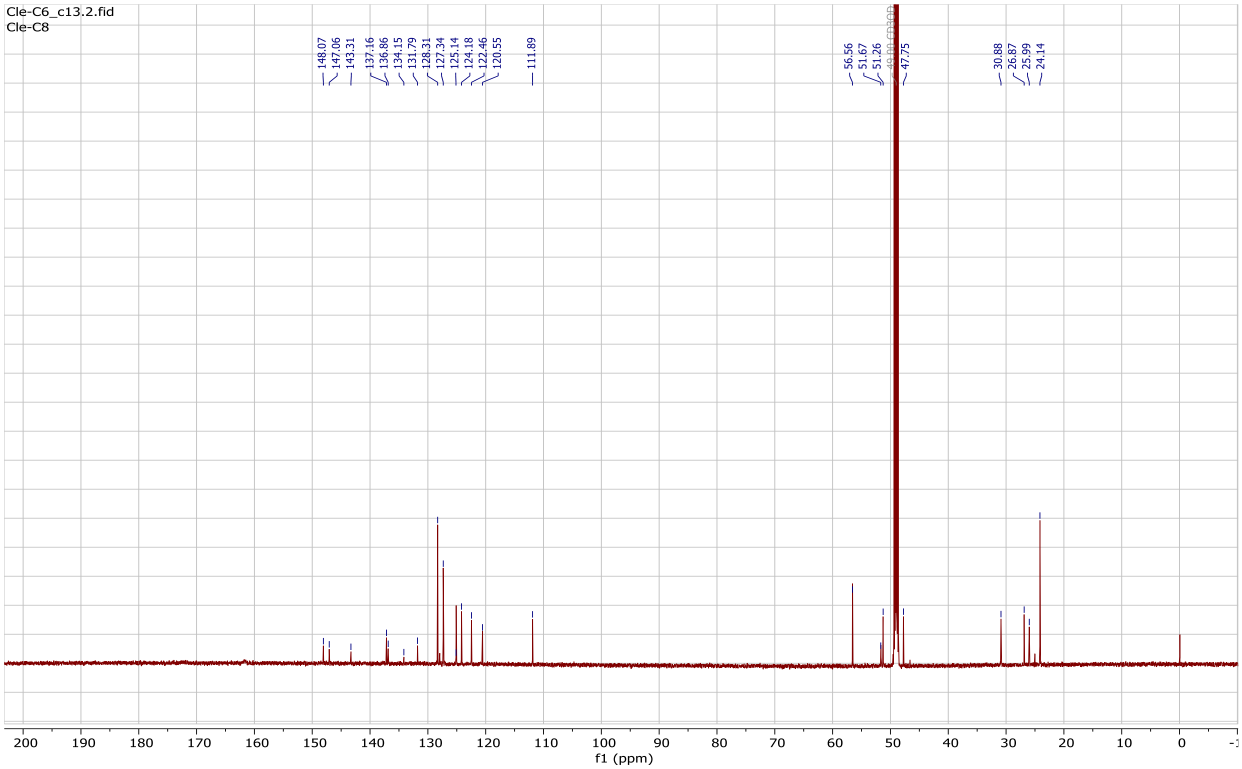

^{1}H (700 MHz, $CDCl_3$) and ^{13}C (175 MHz, $CDCl_3$) NMR spectrum of compound Cle-C7

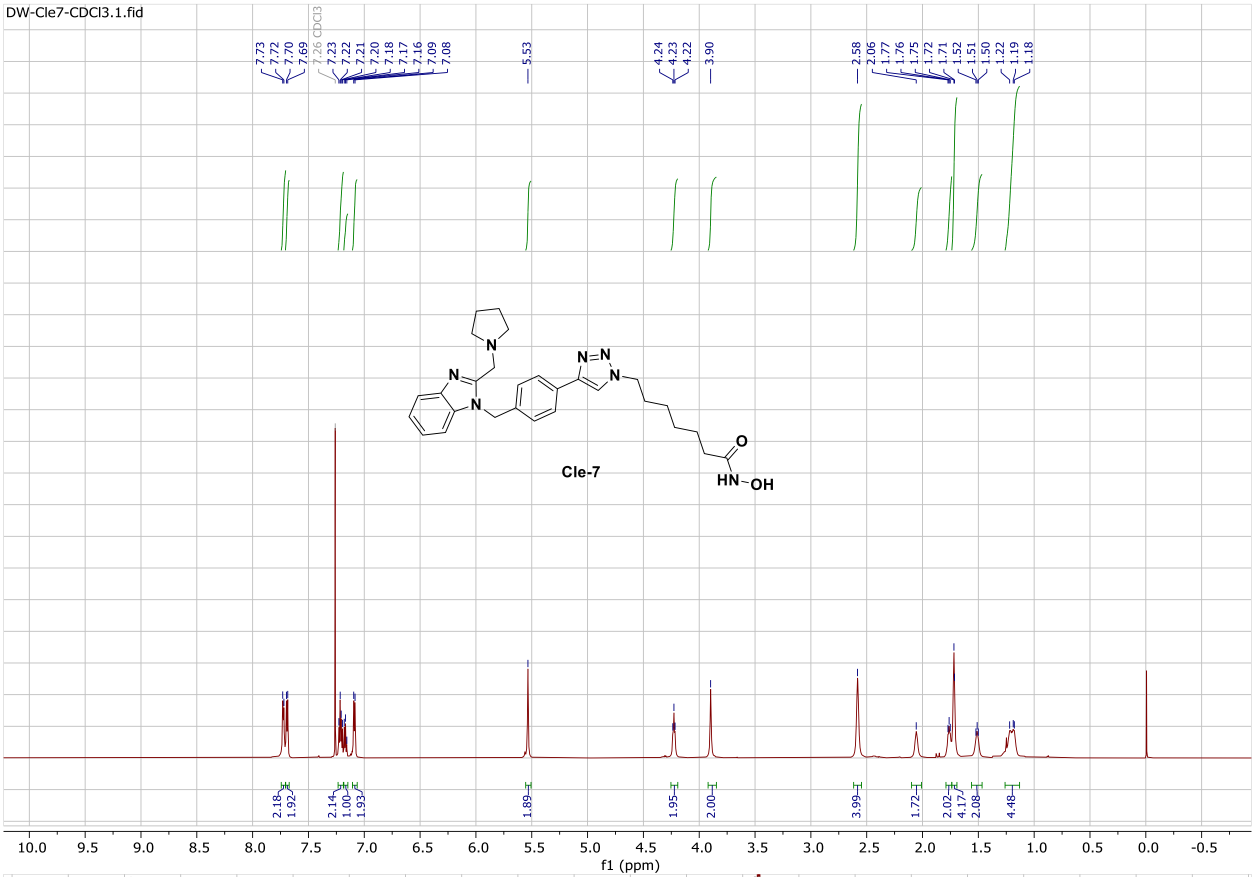

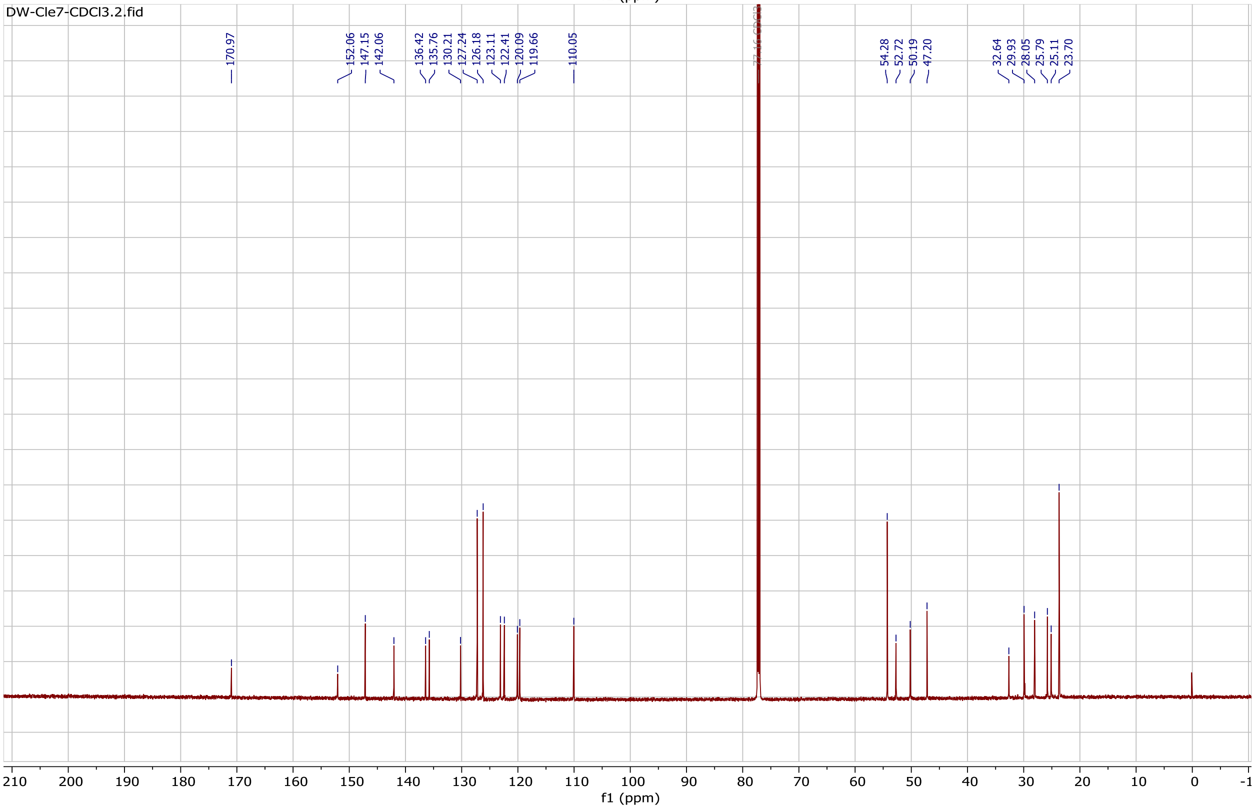

^{1}H (500 MHz, CD_3OD) and ^{13}C (175 MHz, CD_3OD) NMR spectrum of compound Cle-C8

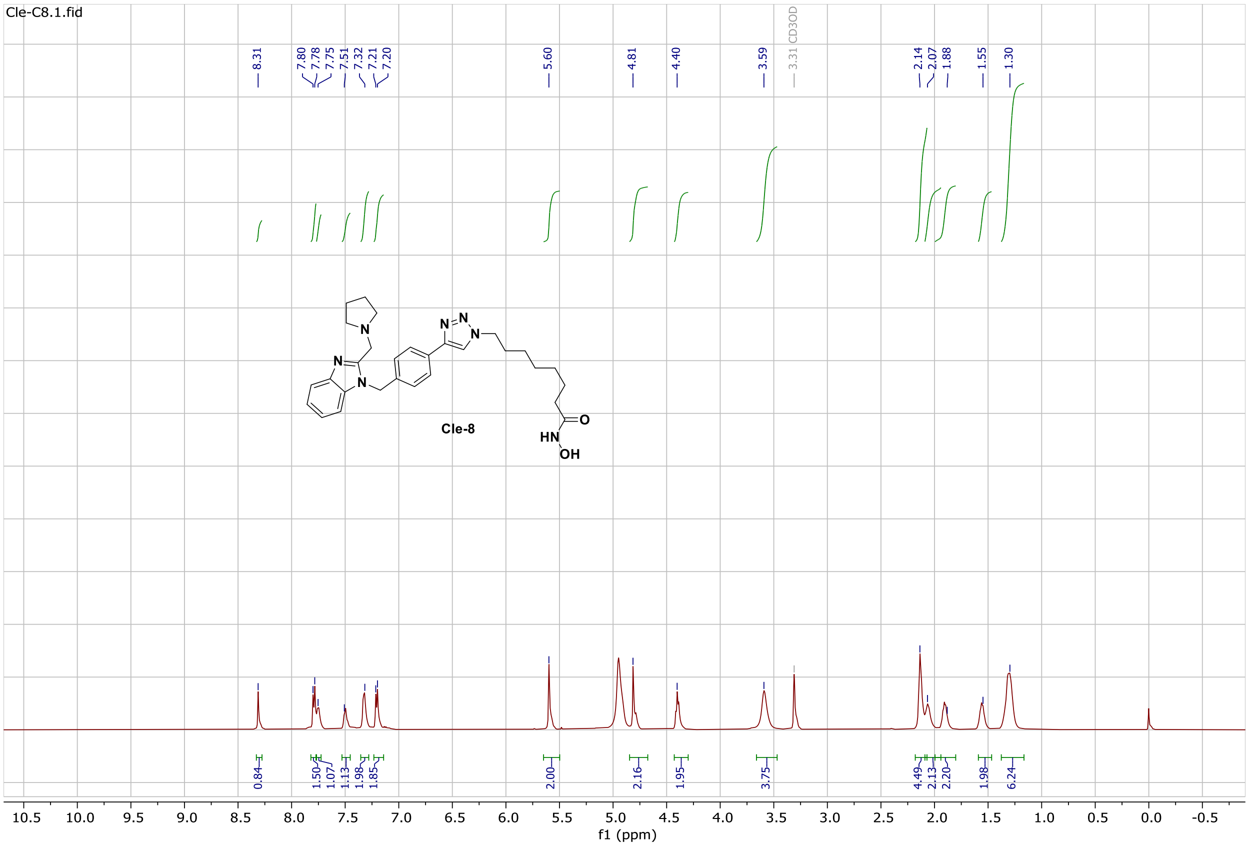

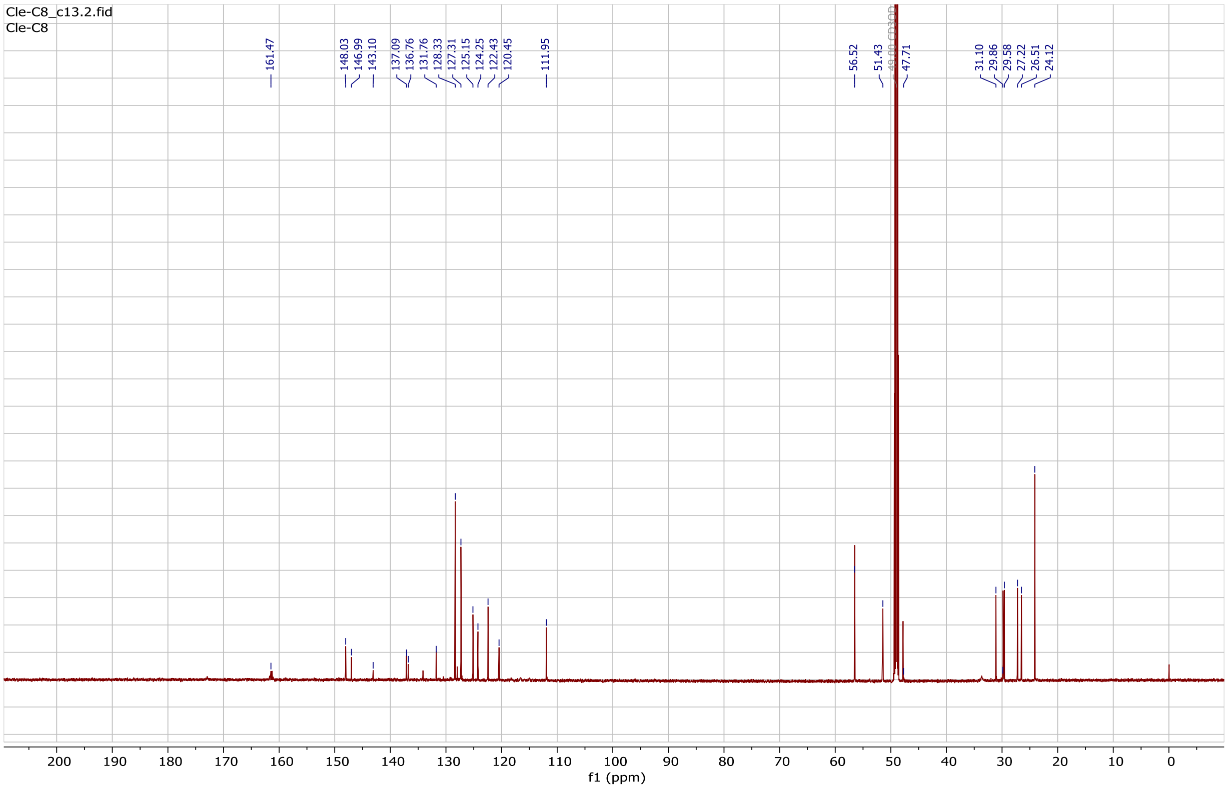

^{1}H (700 MHz, CD_3OD) and ^{13}C (175 MHz, CD_3OD) NMR spectrum of compound Cle-PH

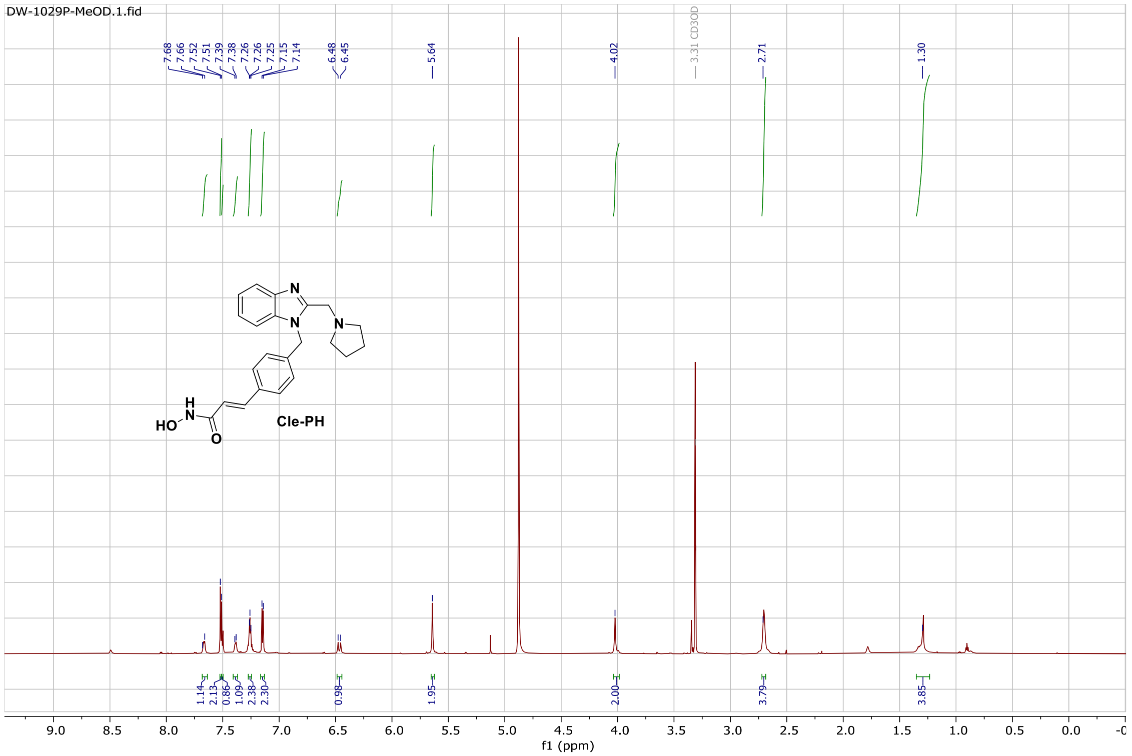

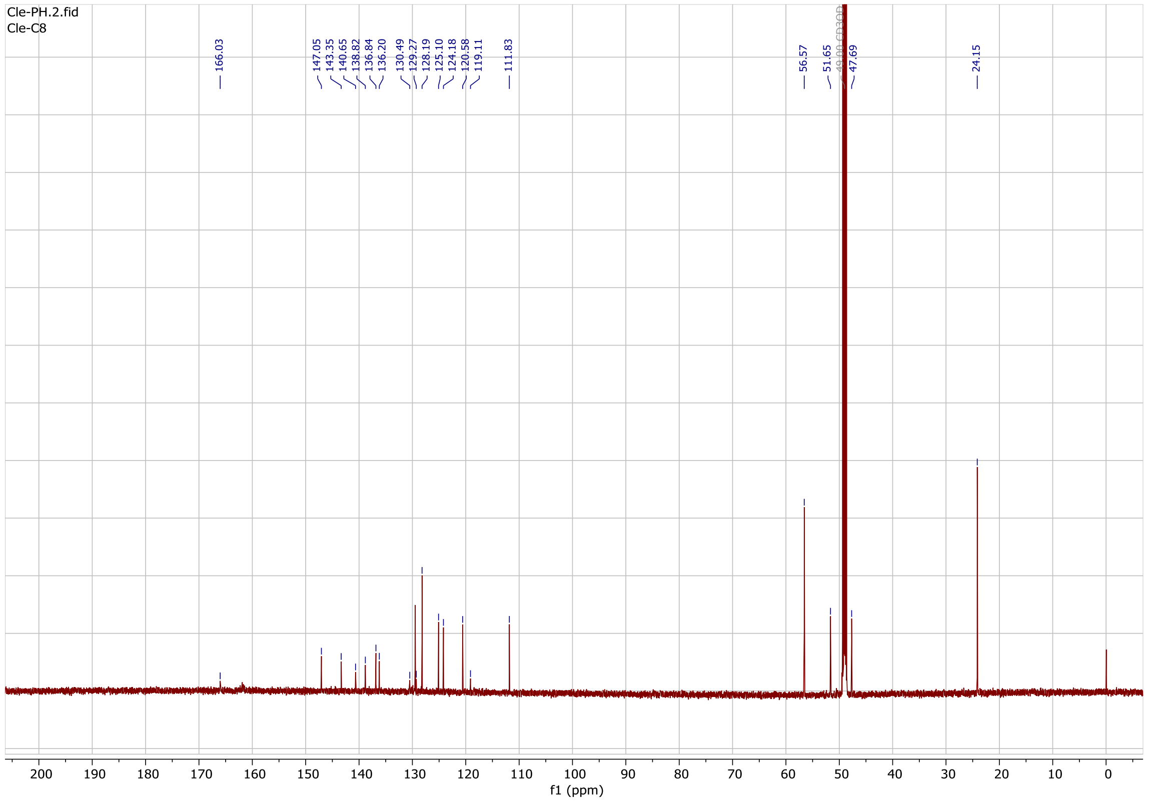

^{1}H (500 MHz, CD_3OD) and ^{13}C (175 MHz, CD_3OD) NMR spectrum of compound Cle-PPH

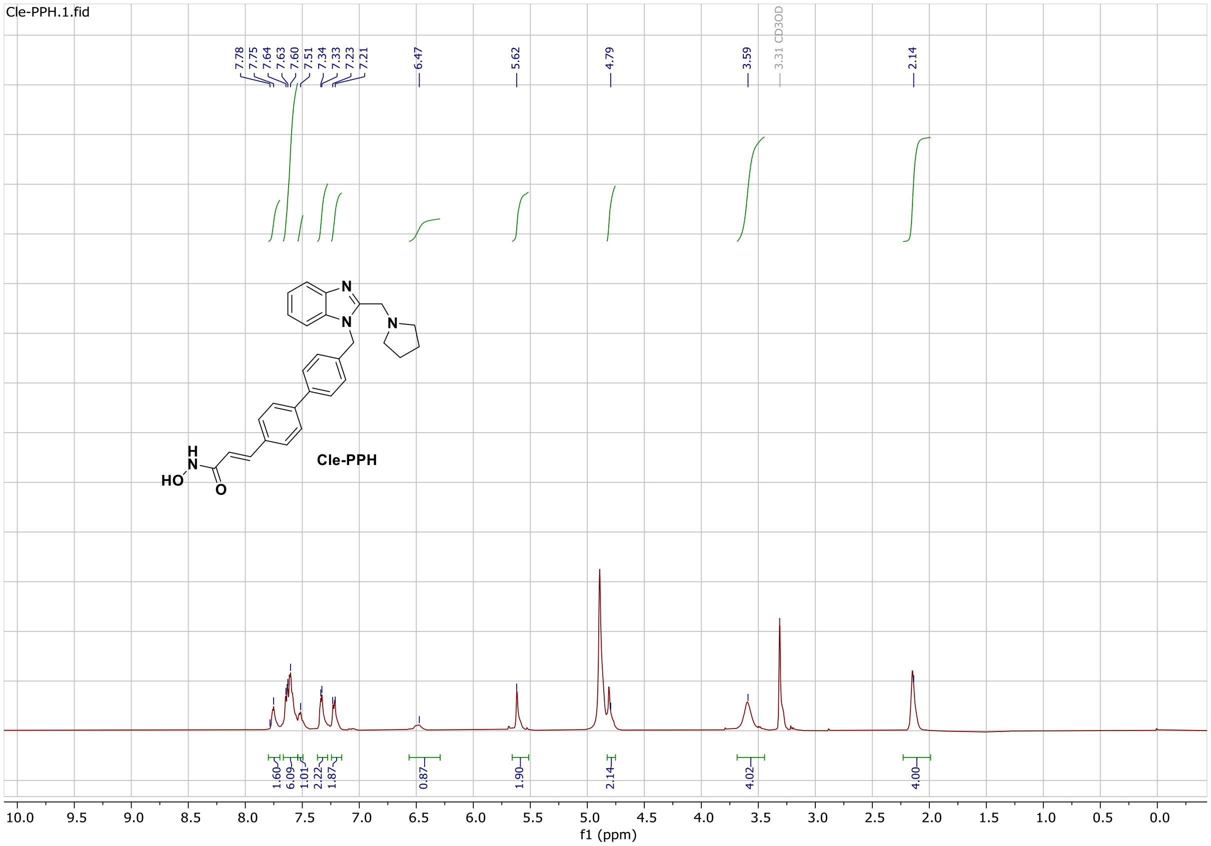

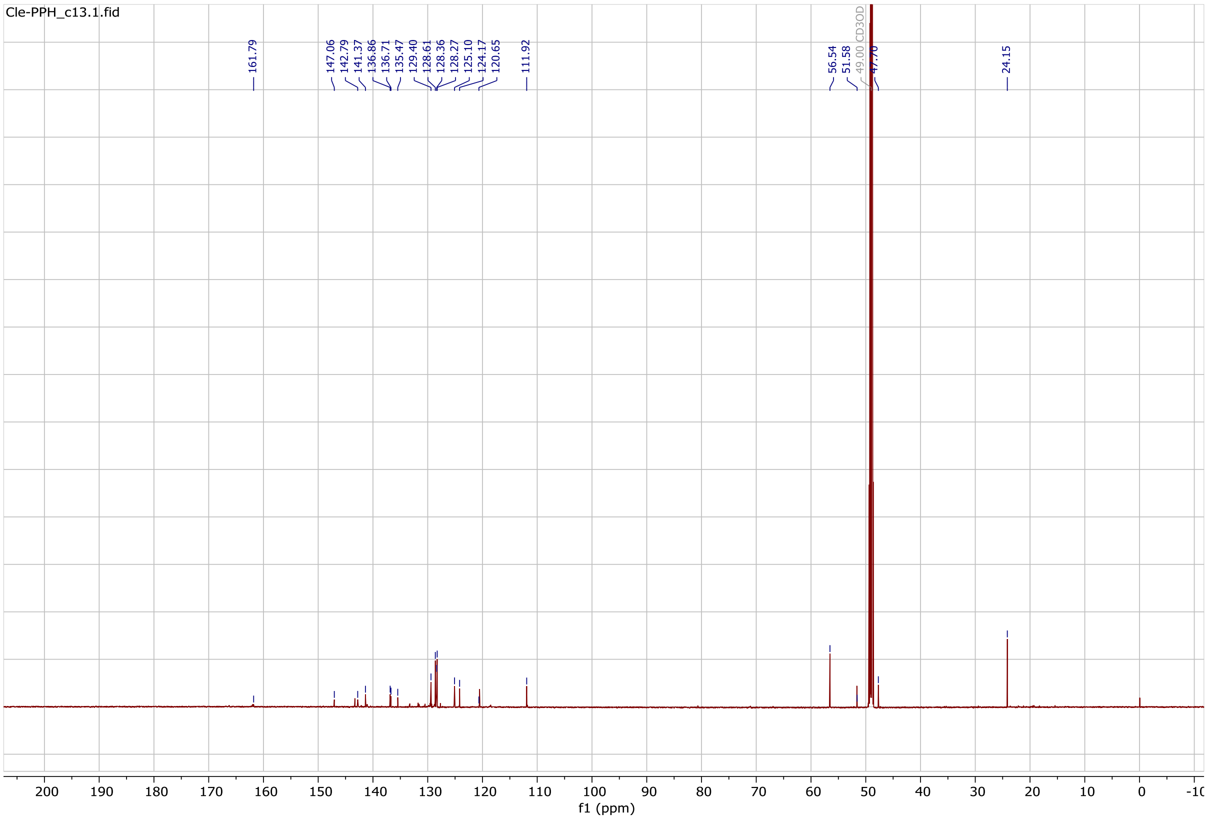

^{1}H (700 MHz, CD_3OD) and ^{13}C (175 MHz, CD_3OD) NMR spectrum of Compound Cle-C4K

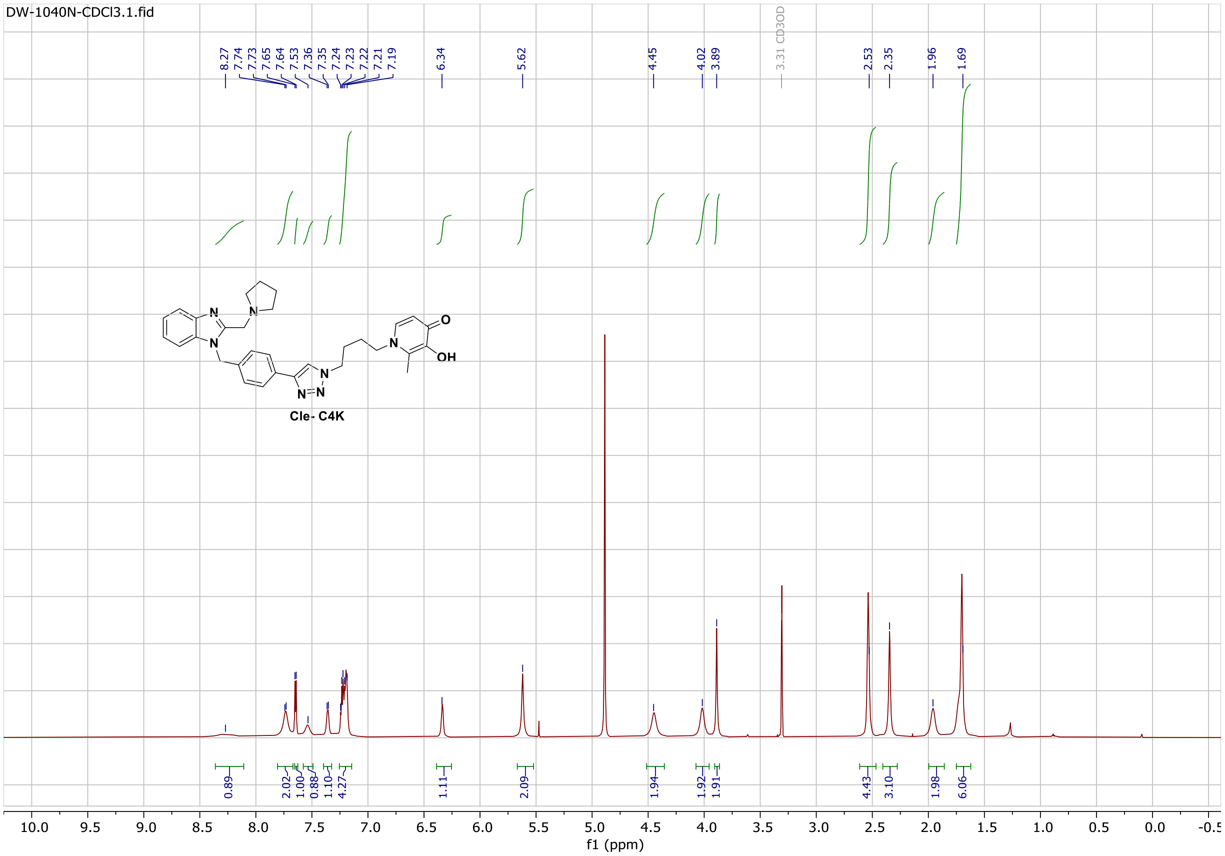

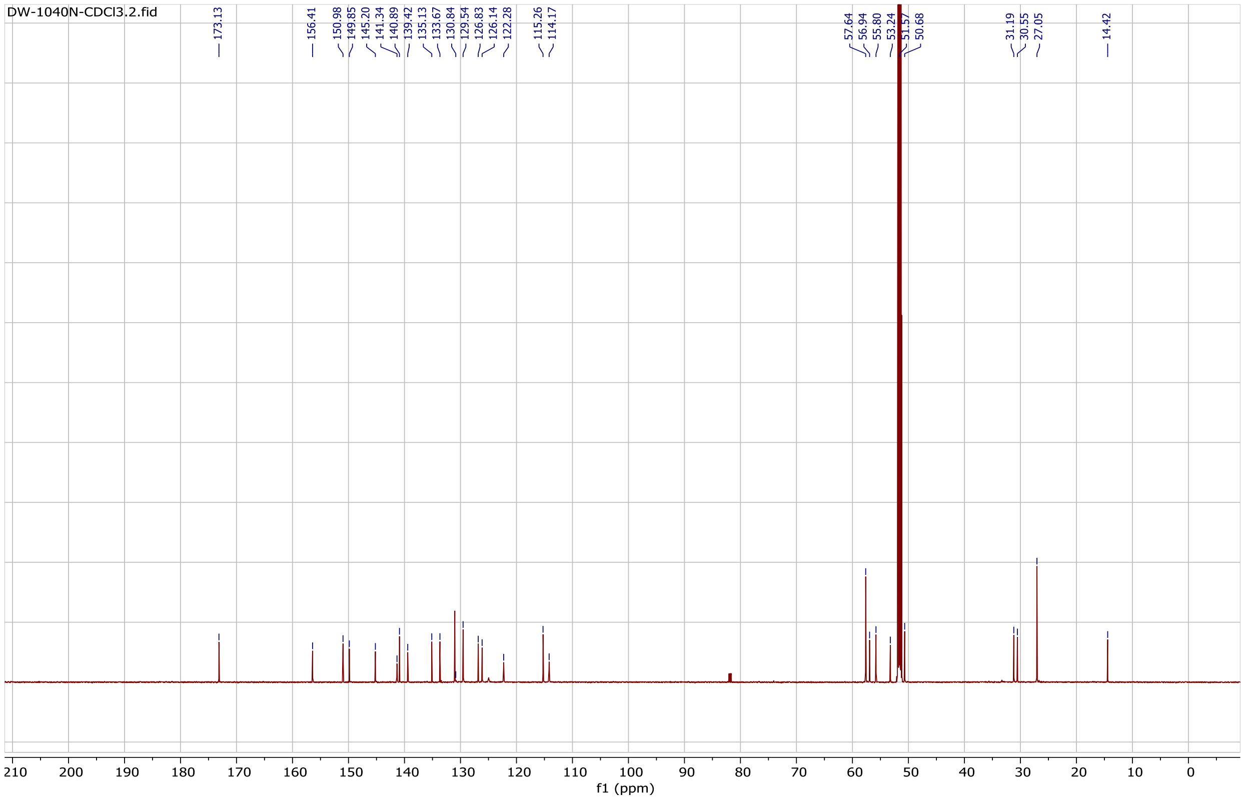

^{1}H (700 MHz, CD_3OD) and ^{13}C (175 MHz, CD_3OD) NMR spectrum of Compound Cle-C5K

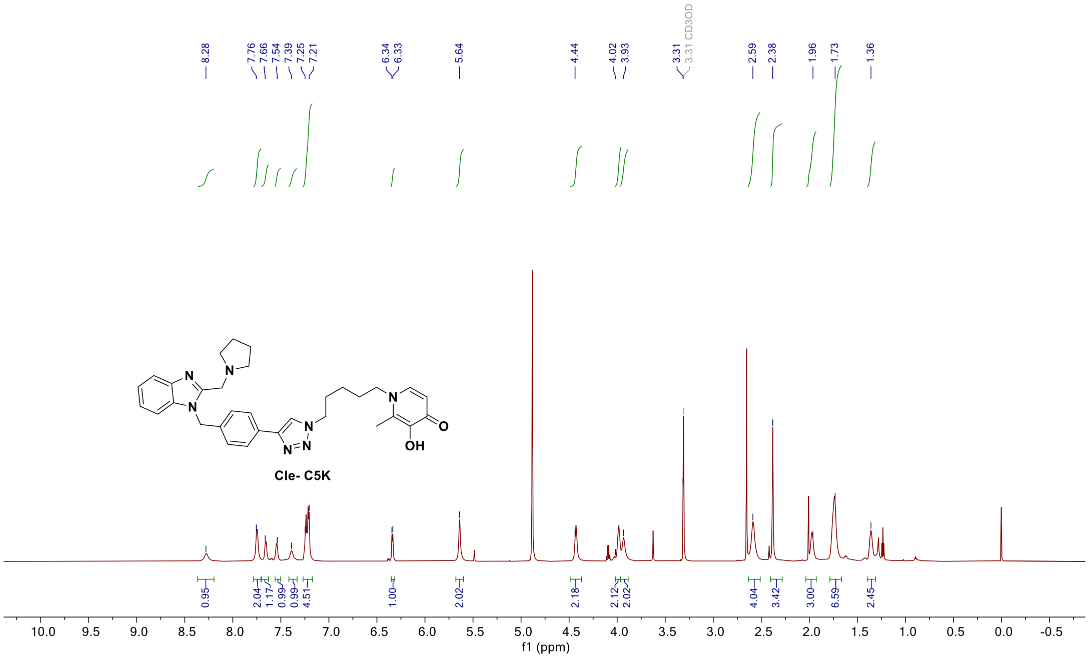

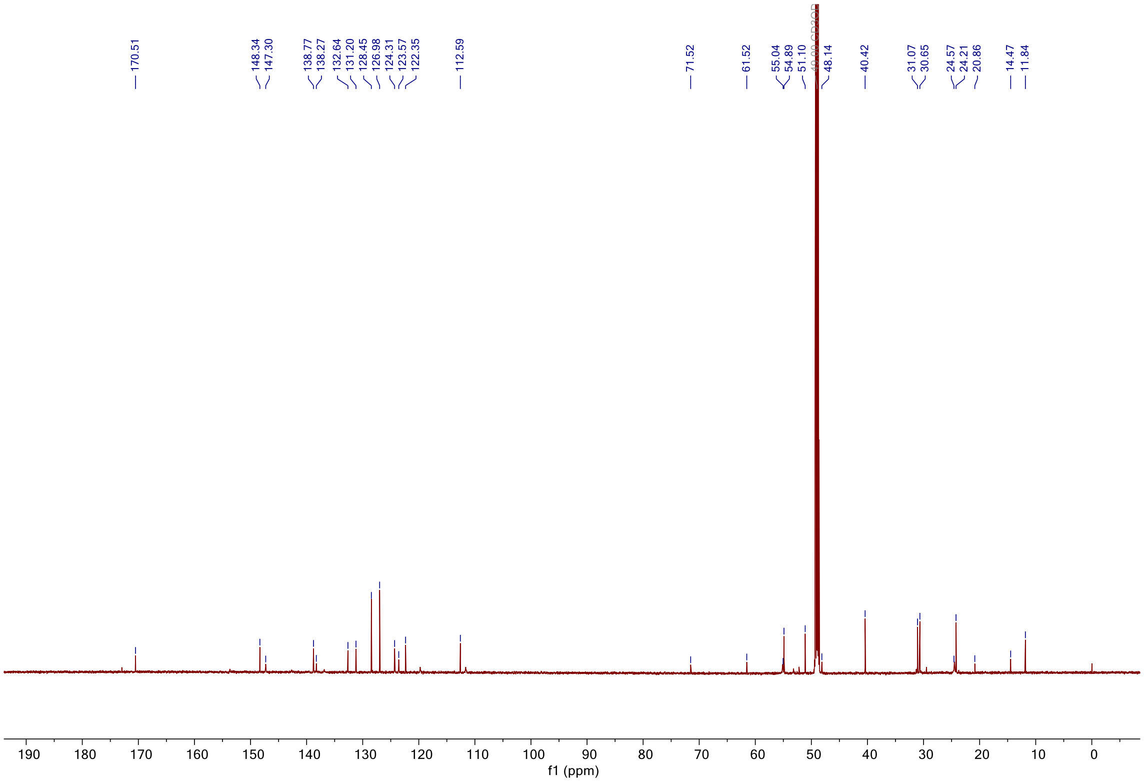

^{1}H (700 MHz, CD_3OD) and ^{13}C (175 MHz, CD_3OD) NMR spectrum of Compound Cle-C6K

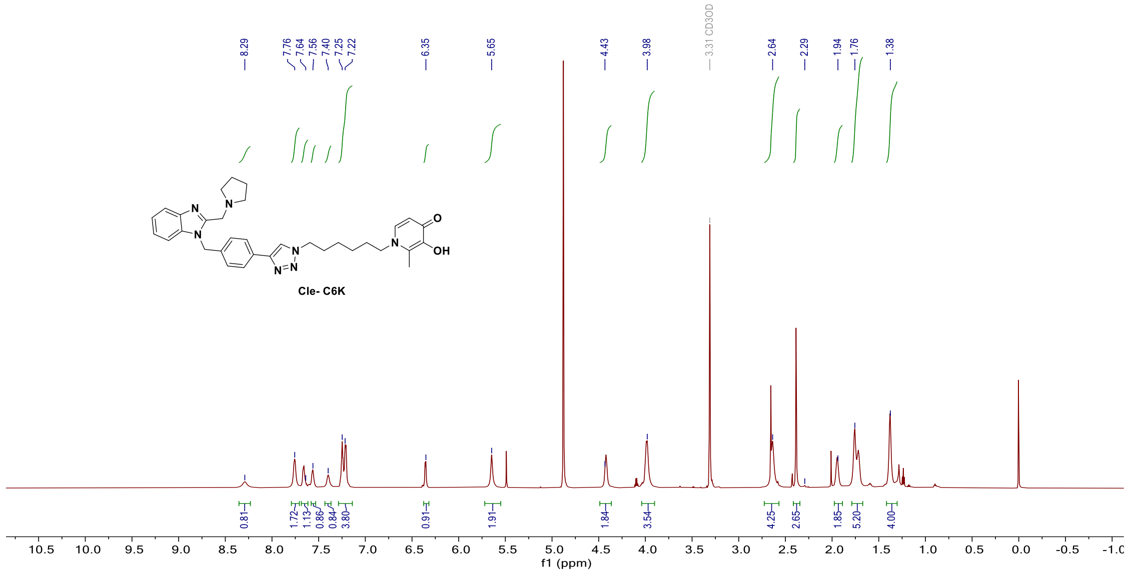

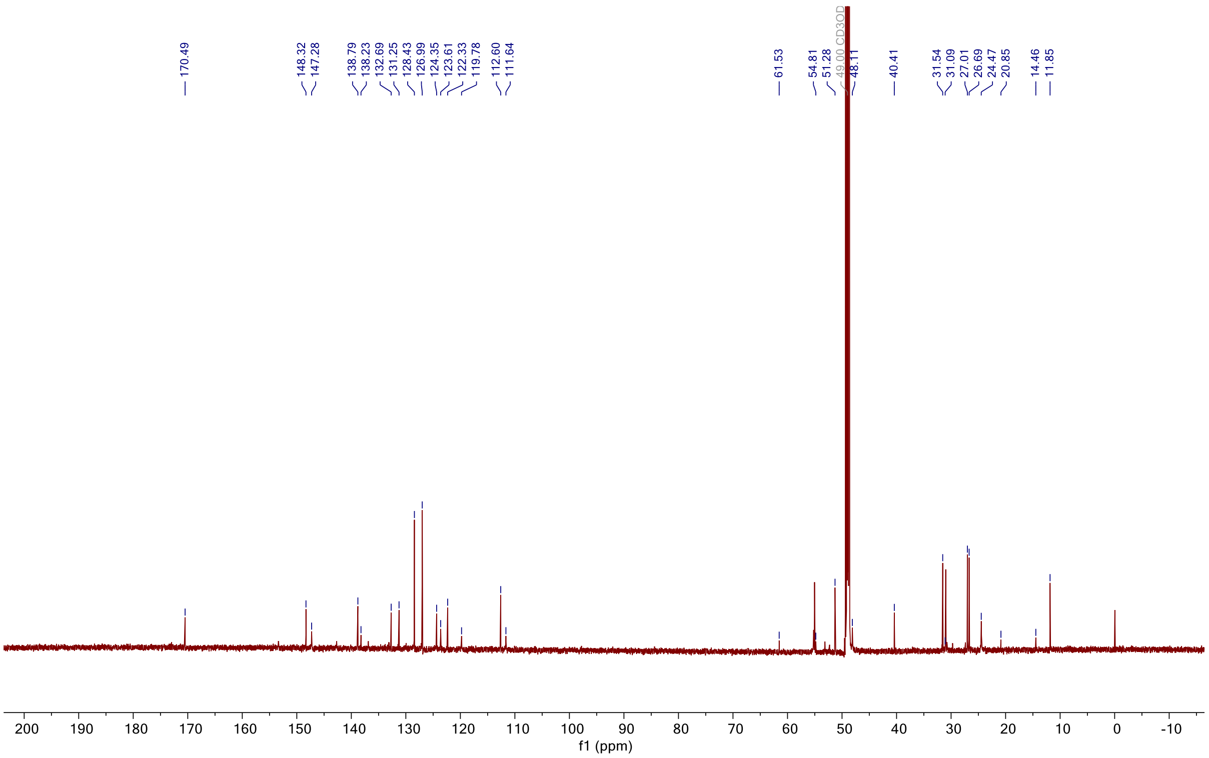

^{1}H (700 MHz, $CDCl_3$) and ^{13}C (175 MHz, $CDCl_3$) NMR spectrum of Compound Cle-C7K

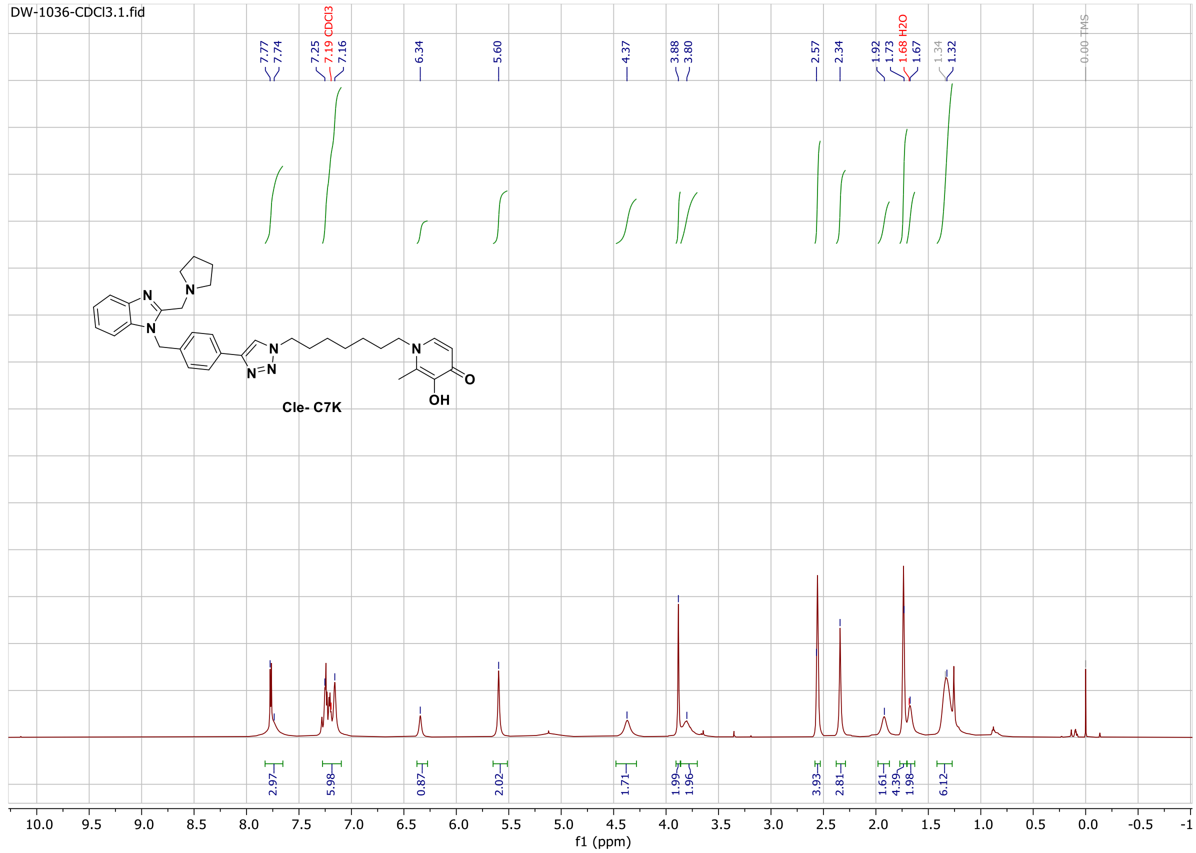

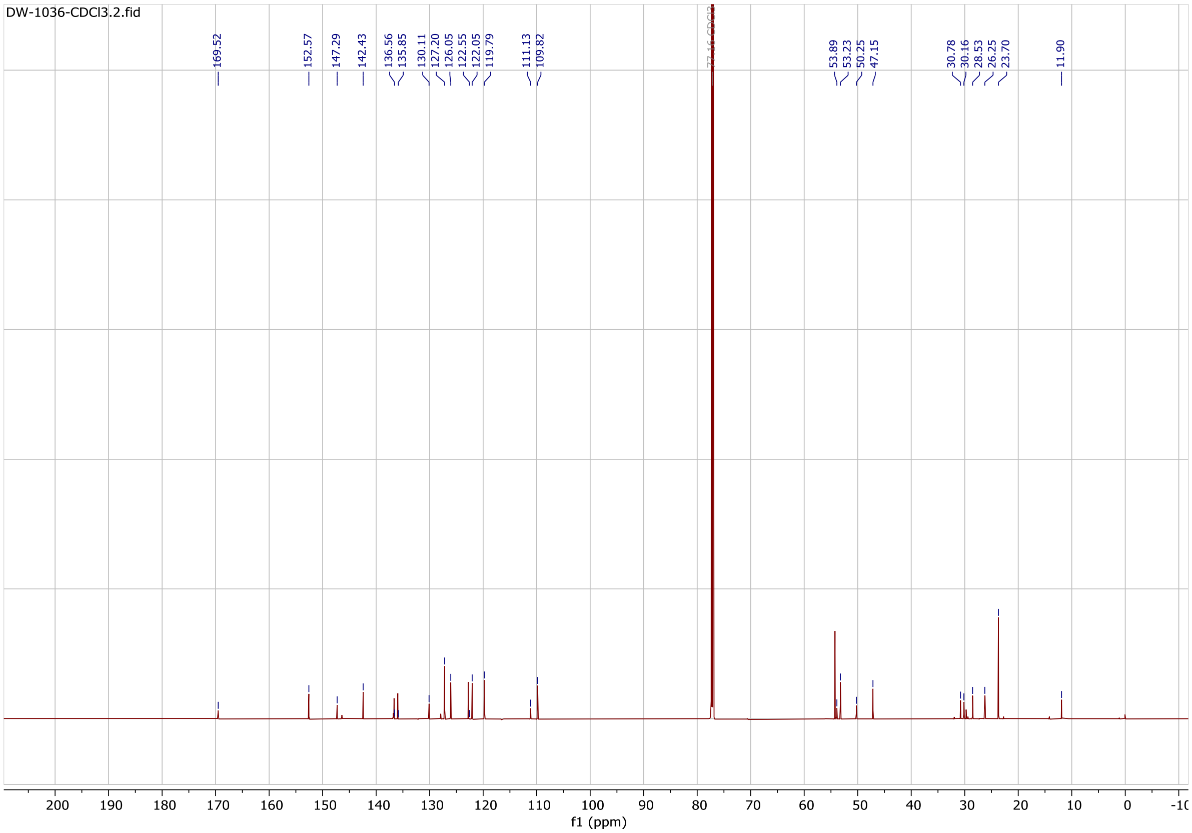

^{1}H (700 MHz, CD_3OD) and ^{13}C (175 MHz, CD_3OD) NMR spectrum of Compound Cle-C8K

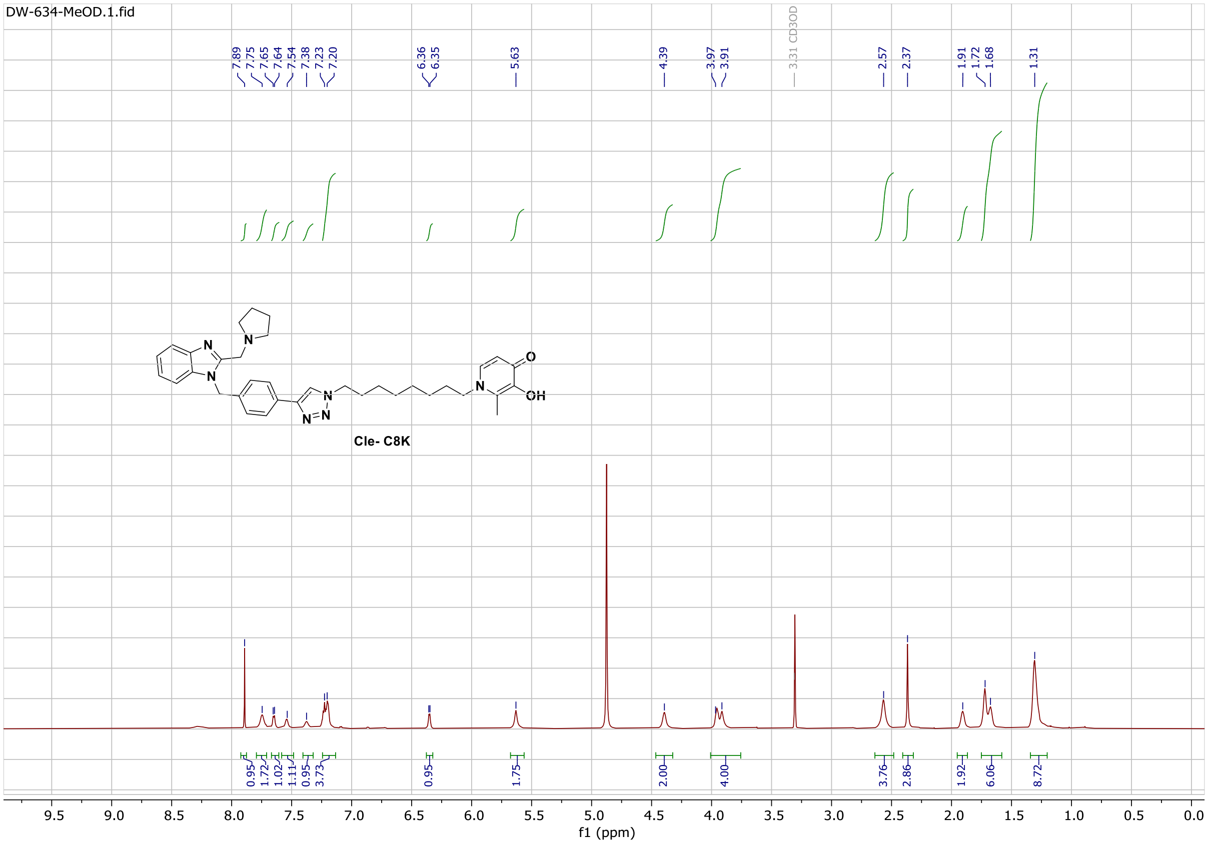

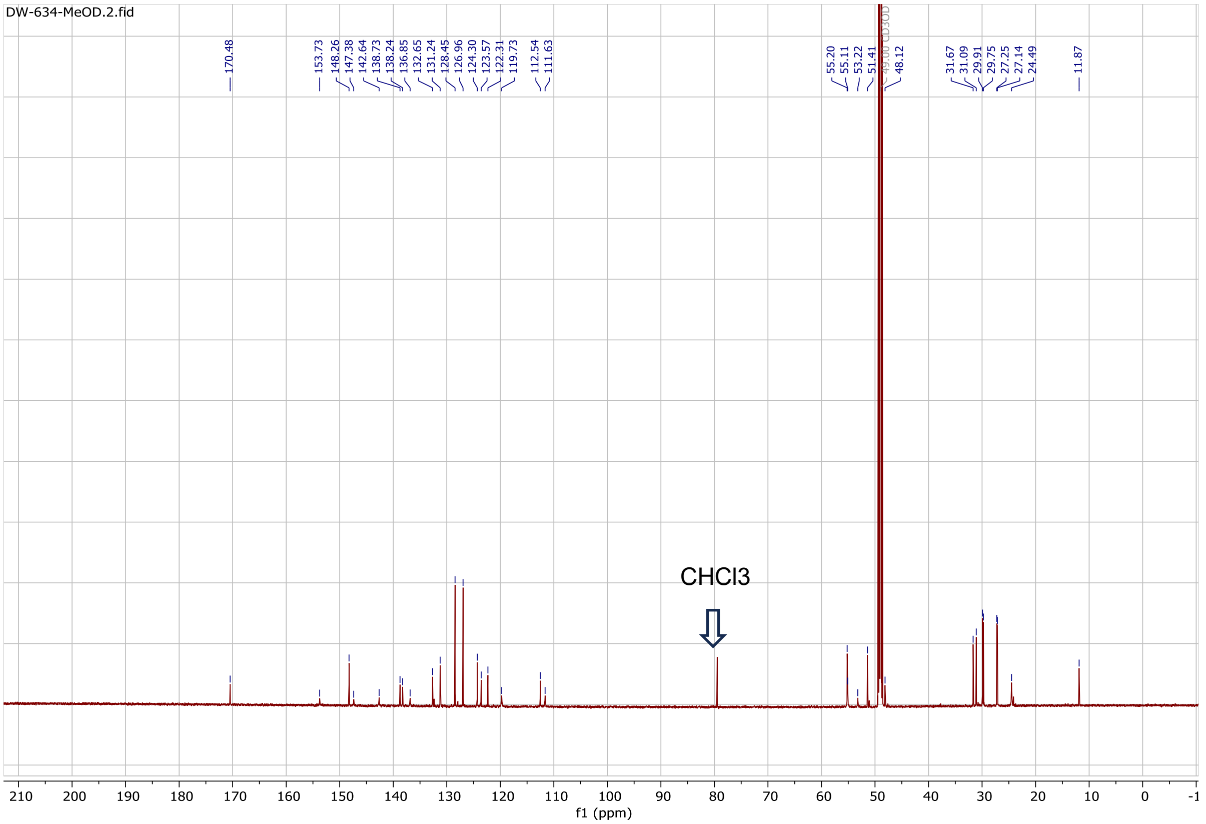

^{1}H (700 MHz, CD_3OD) and ^{13}C (175 MHz, CD_3OD) NMR spectrum of Compound Cle-AC-6K

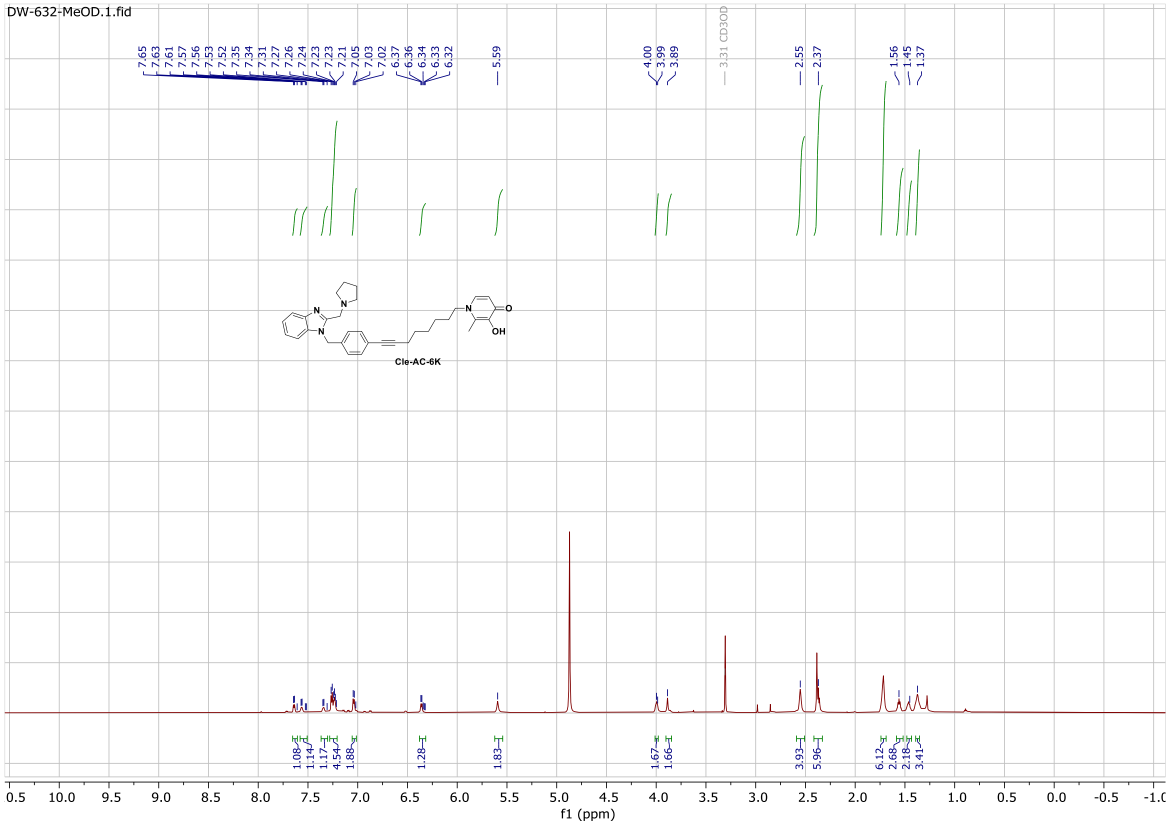

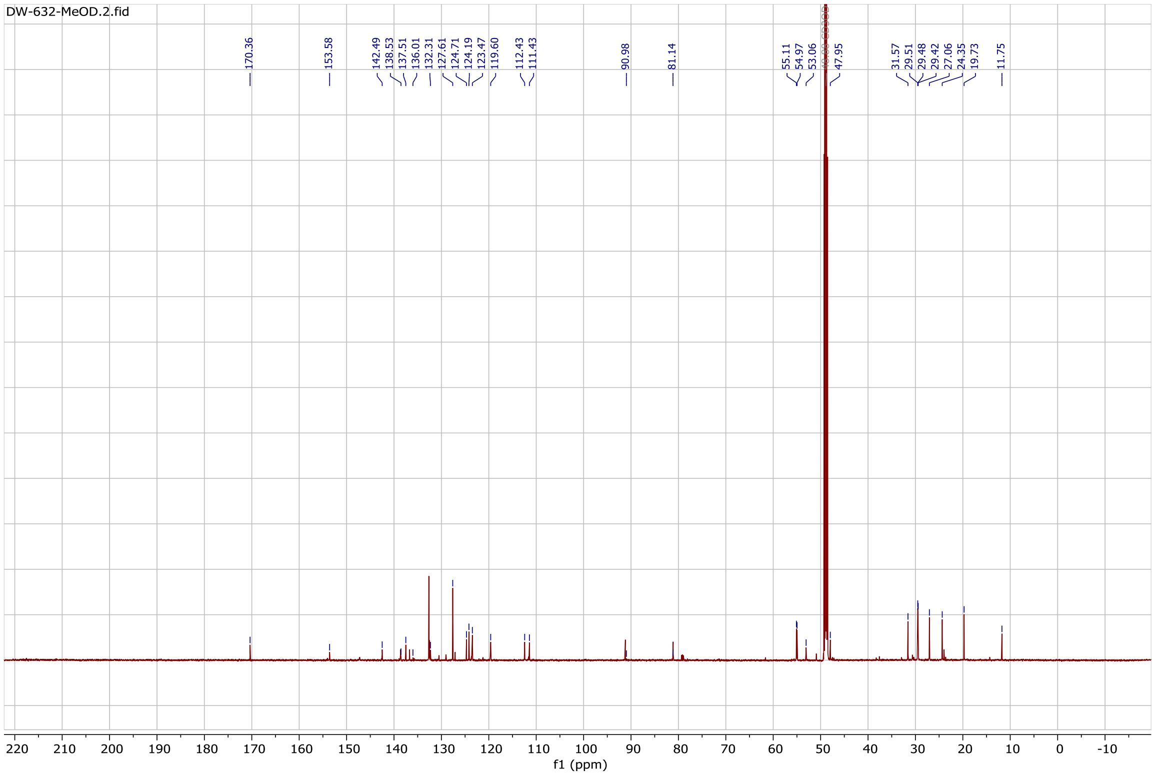

^{1}H (700 MHz, $CDCl_3$) and ^{13}C (175 MHz, $CDCl_3$) NMR spectrum of Compound Cle-AC-7K

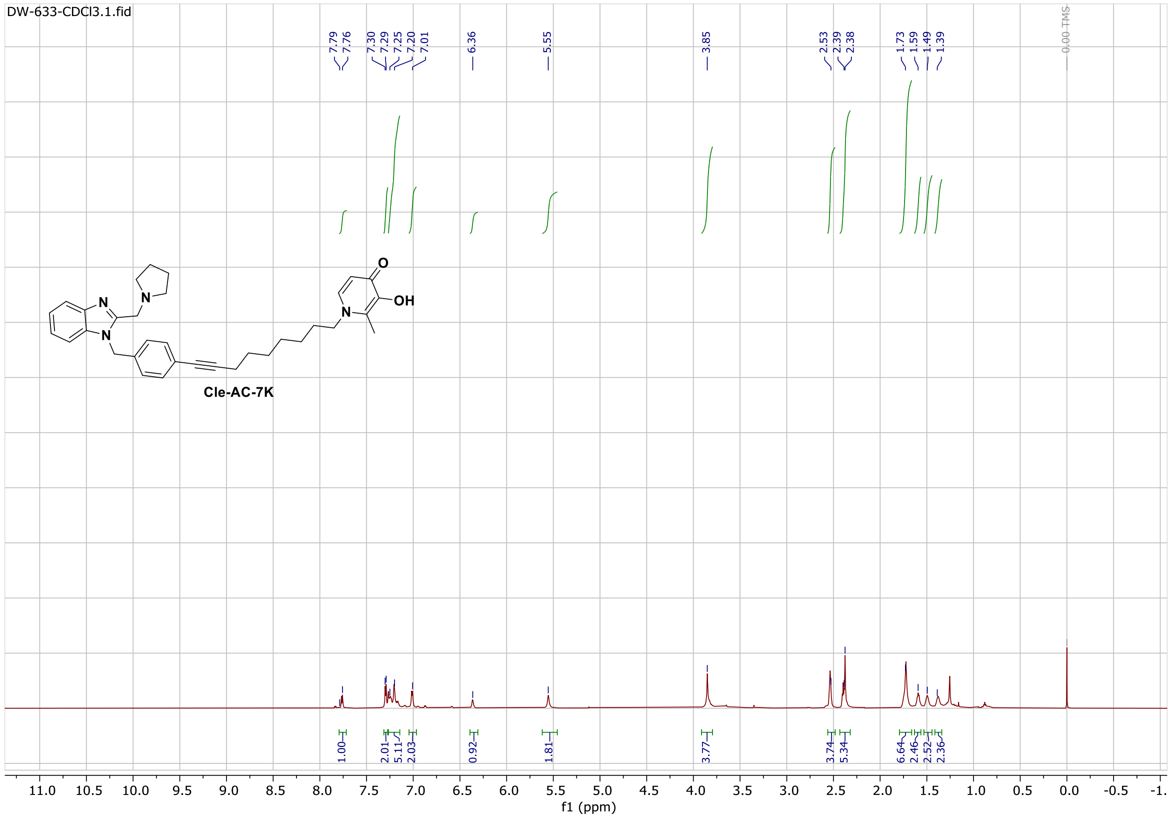

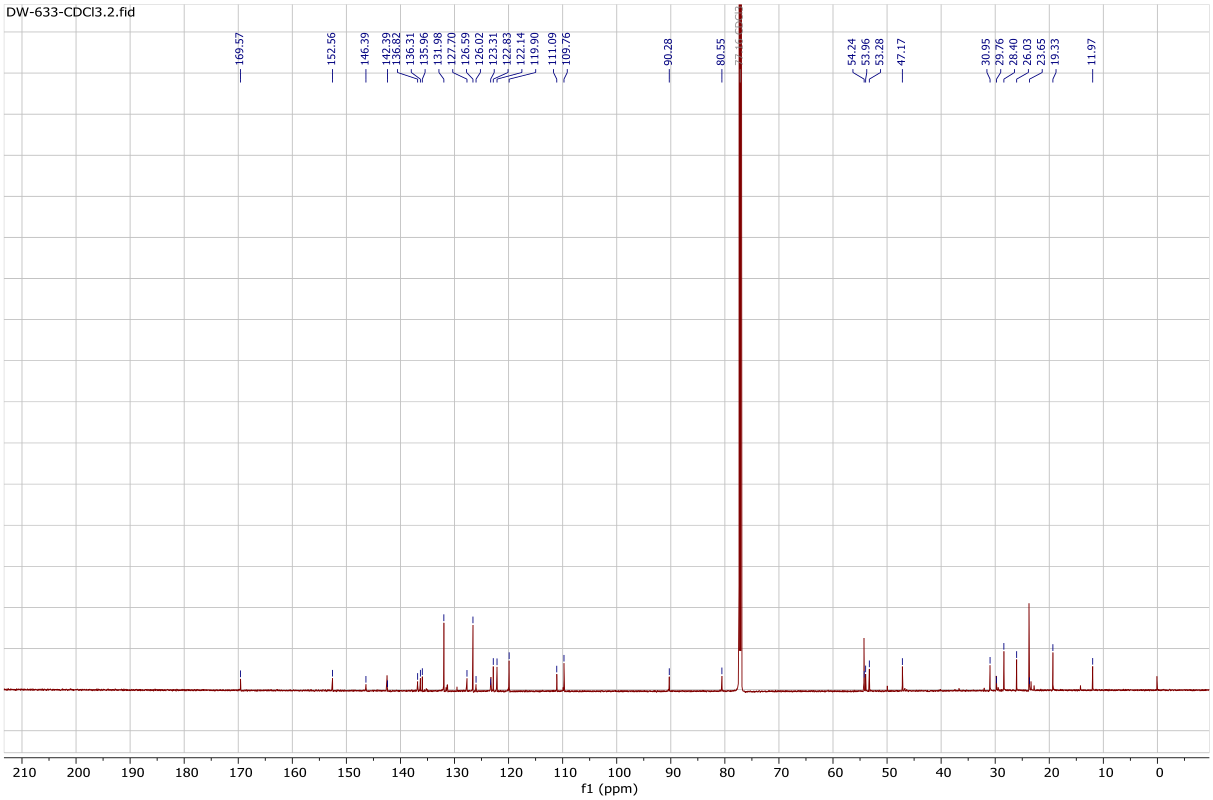

^{1}H (700 MHz, $CDCl_3$) and ^{13}C (175 MHz, $CDCl_3$) NMR spectrum of Compound Cle-AC-8K

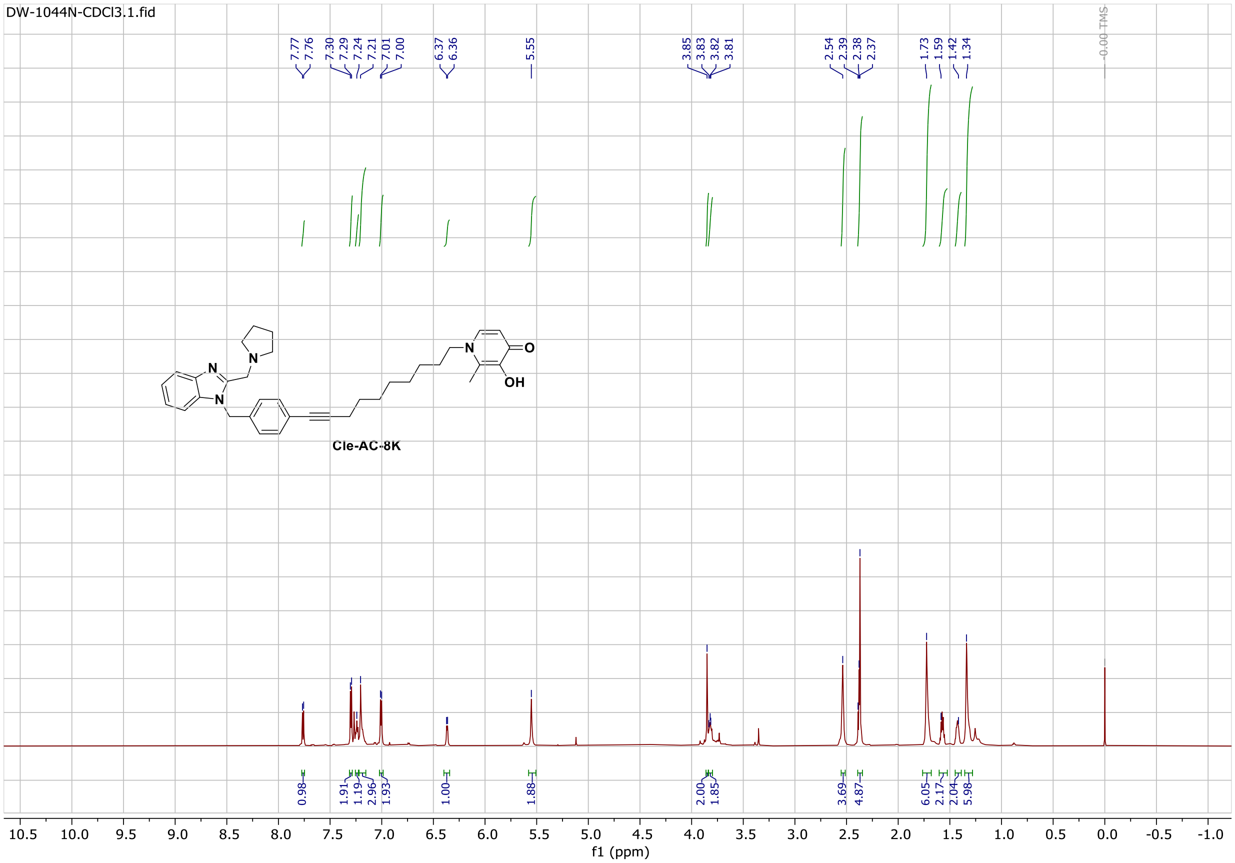

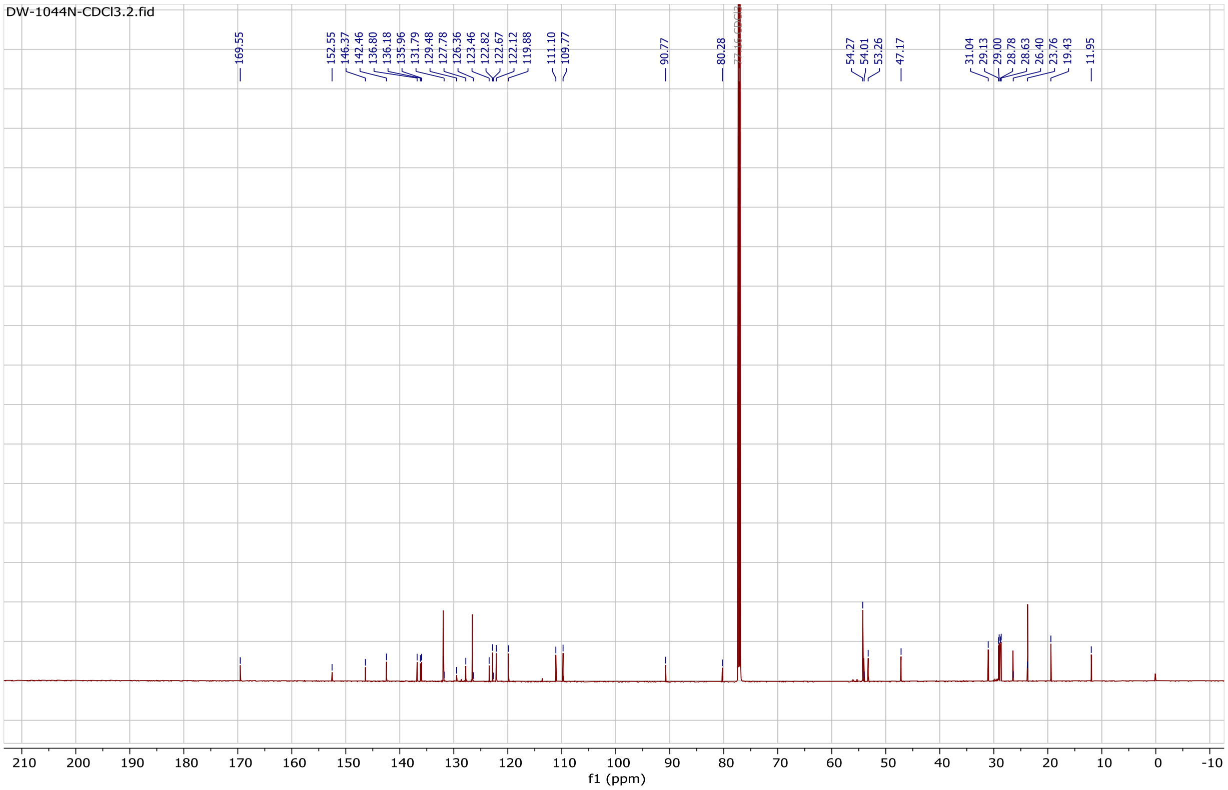

1.HPLC Purity and UV peak at 254 nm of compound Cle-C6

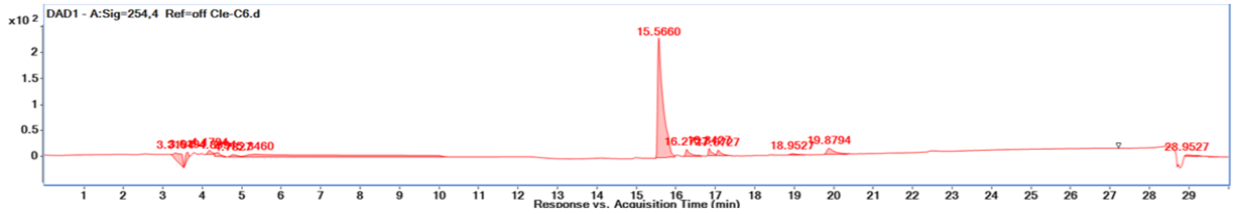

2.HPLC Purity and UV peak at 254 nm of compound Cle-C7

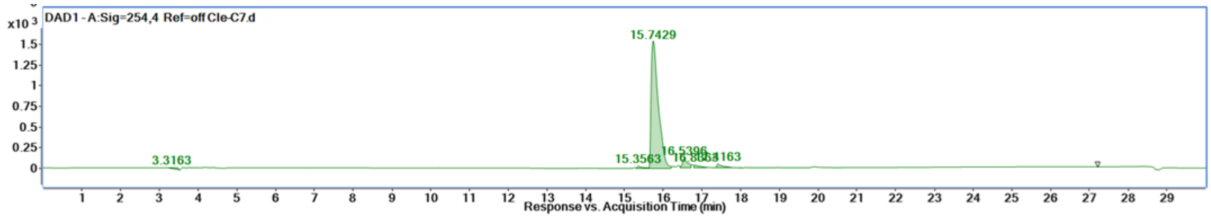

3.HPLC Purity and UV peak at 254 nm of compound Cle-C8

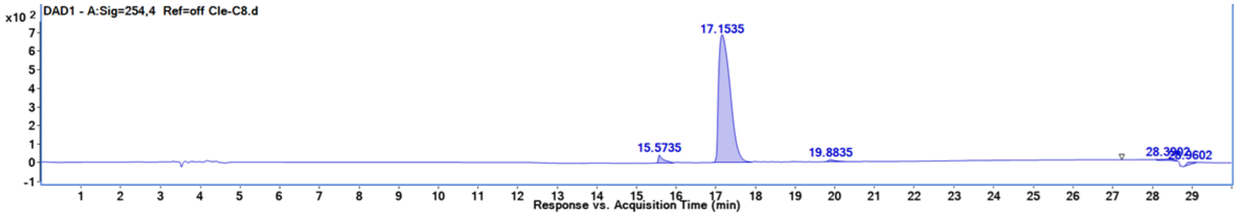

4. HPLC Purity and UV peak at 254 nm of compound Cle-PH

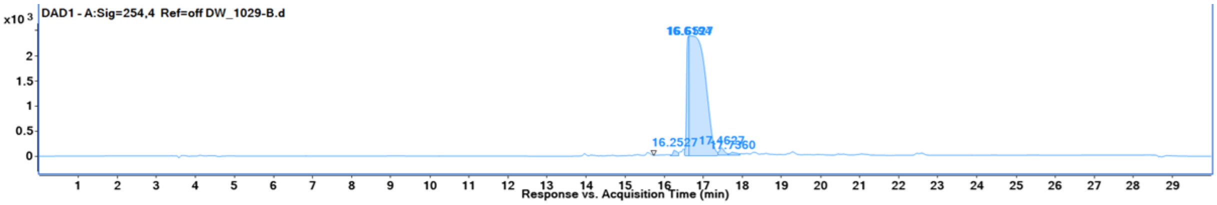

5. HPLC Purity and UV peak at 254 nm of compound Cle-PPH

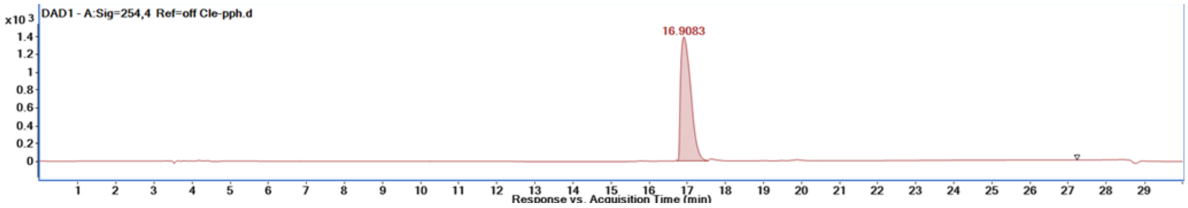

6. HPLC Purity and UV peak at 254 nm of compound Cle-C4K

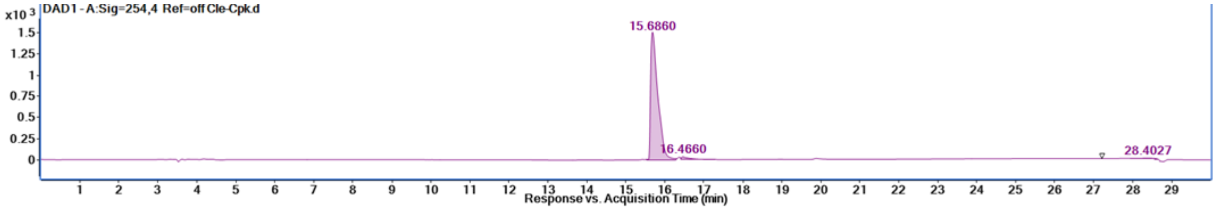

7. HPLC Purity and UV peak at 254 nm of compound Cle-C5K

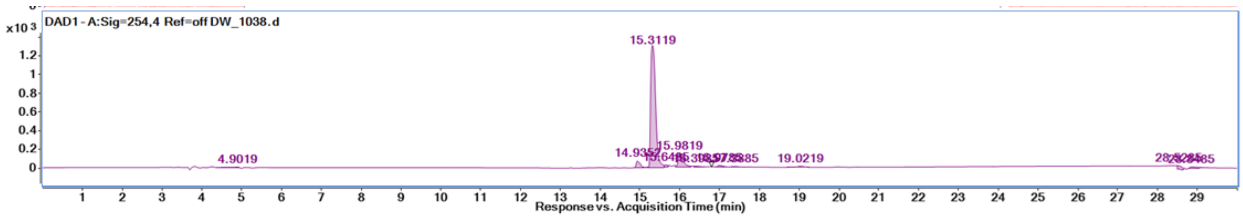

8. HPLC Purity and UV peak at 254 nm of compound Cle-C6K

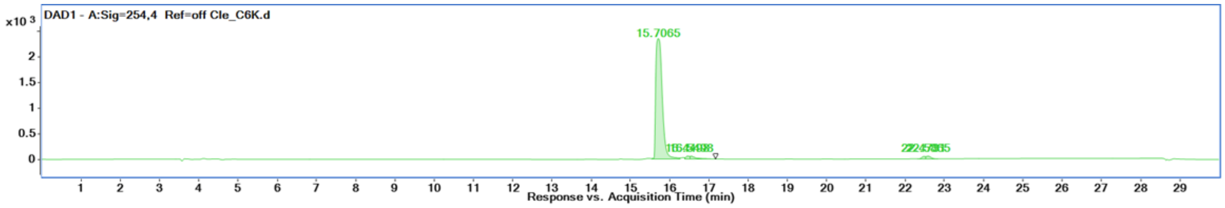

9. HPLC Purity and UV peak at 254 nm of compound Cle-C7K

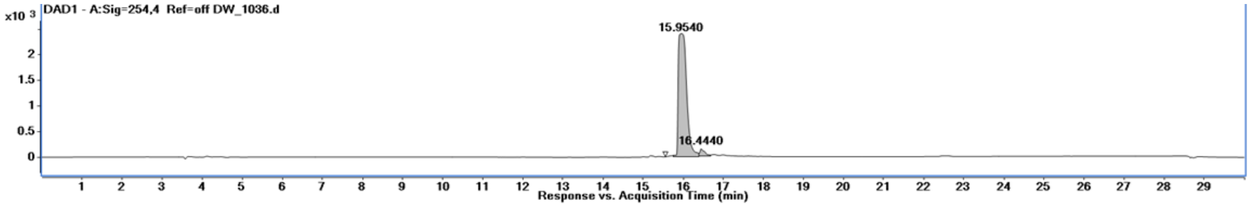

10. HPLC Purity and UV peak at 254 nm of compound Cle-C8K

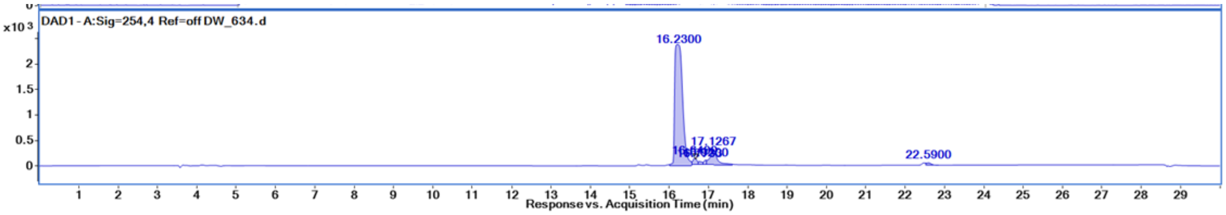

11. HPLC Purity and UV peak at 254 nm of compound Cle-AC-6K

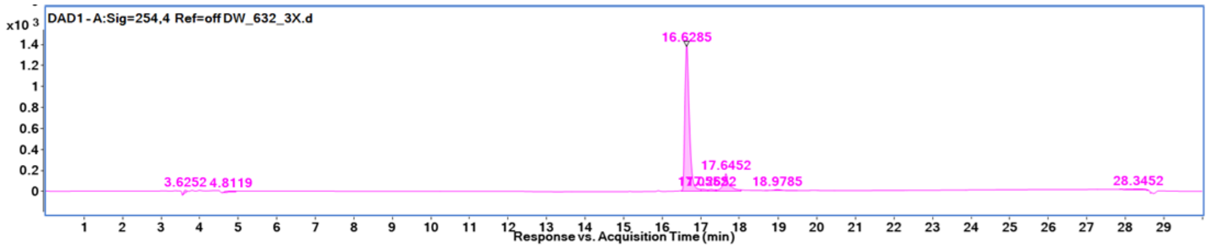

12. HPLC Purity and UV peak at 254 nm of compound Cle-AC-7K

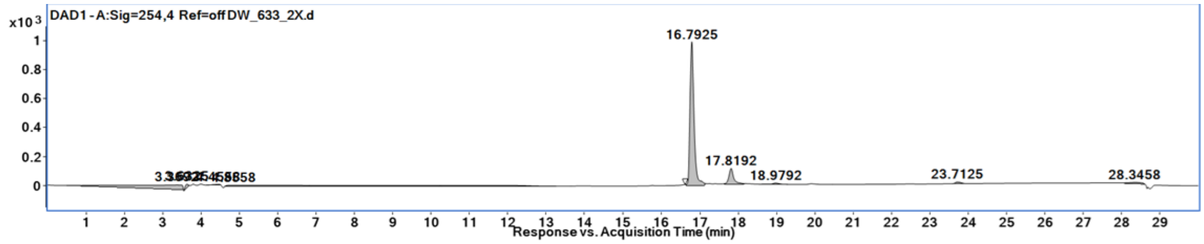

13. HPLC Purity and UV peak at 254 nm of compound Cle-AC-8K

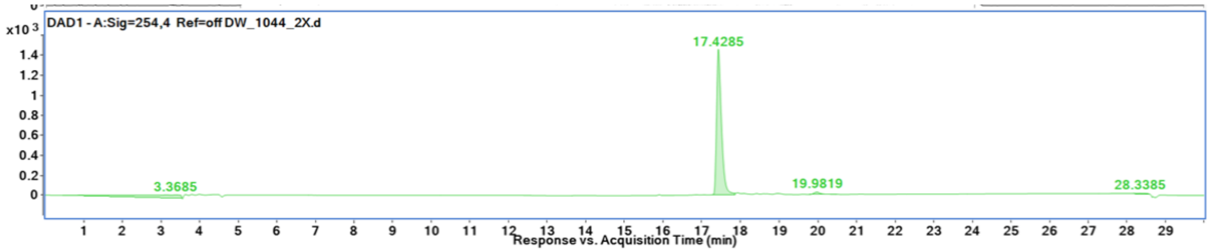

Supplement: Supplementary file 1 [file jm5c02018_si_001.pdf]
